# Integrated metagenomics and metabolomics analyses revealed biomarkers in β-casein A2A2-type cows

**DOI:** 10.3389/fvets.2024.1438717

**Published:** 2024-10-01

**Authors:** Jinyan Zhao, Chuanchuan Wang, Jiahuan Hu, Ruoshuang Ma, Baojun Yu, Wei Zhao, Hua Wang, Yaling Gu, Juan Zhang

**Affiliations:** ^1^Key Laboratory of Molecular Cell Breeding for Ruminants, Yinchuan, China; ^2^Ningxia University College of Animal Science and Technology, Yinchuan, China

**Keywords:** Holstein dairy cows, beta-casein, A2A2, milk fat percentage, metabolomics, metagenomics

## Abstract

In Holstein cows, β-casein, one of the most critical proteins in milk, exists in two main genotypes, A1 and A2. Herein, 45 Holstein cows [categorized into three groups based on β-casein A1A1, A1A2, and A2A2 genotypes (*N* = 15)] with the same feeding management and litter size were enrolled to explore differences in rumen microflora and metabolites across various β-casein genotypes. Rumen fluids were collected for metagenomics and metabolomics analyses. Metabolomics and weighted gene co-expression network analysis (WGCNA) revealed that arachidonic acid (AA), adrenic acid (AdA), glycocholic acid (GCA), and taurocholic acid (TCA) were significantly and positively correlated with milk fat % in dairy cows (*p* < 0.05). Furthermore, macro-genomics and Spearman’s correlation analysis revealed significant positive correlations (*p* < 0.05) between the characteristic flora (*g_Acetobacter*, *g_Pseudoxanthomonas*, *g_Streptococcus*, and *g_Pediococcus*) and the five characteristic metabolites in the rumen of A2A2 dairy cows. Moreover, functional enrichment analysis revealed more genes enriched to the TRP channel’s inflammatory mediator-regulated pathway and the mTOR signaling pathway in A2A2 genotyped cows. Additionally, the regulatory effects of AA on bovine mammary epithelial cells (BMECs) were examined using CCK-8, EdU, and qRT-PCR assays, revealing that AA promoted triglyceride (TG) synthesis and upregulated the milk fat marker genes including *SREBF1*, *ACSS2*, *AGPAT6*, and *FASN*. Overall, we identified characteristic microorganisms and metabolites in A2A2 Holstein cows and established that AA could be a biomarker for higher milk fat %.

## Background

Milk, one of the body’s primary sources of nutrients, is rich in lactose, triglycerides (TGs), proteins, minerals, and vitamins (1). Milk proteins are classified based on their solubility into casein (~80%), whey proteins (~14%), and fat globule membrane proteins (~6%), with casein-soluble proteins being the most abundant and further classified into four categories: α1-casein, α2-casein, β-casein, and κ-casein (2). The structure of β-casein depends on the dairy cow’s breed and genotype, with A1 and A2 as the two main isoforms (3). The disparity between the two isoforms stems from a mutation at position 67, which induces a transformation of the amino acid from histidine (in A1) to proline (in A2), and this is attributed to genetic predetermination (4, 5). Cows with the β-casein A2A2 genotype produce the popular “A2 milk” (3). Additionally, A2A2 genotyped cows have a higher milk fat % than their A1A1 and A1A2 counterparts (6). Furthermore, during digestion and metabolism, A1 β-casein produces β-casein-7, which has been linked with Gastrointestinal (GI) issues and lactose intolerance disorders in humans (7, 8). It is also noteworthy that A1 β-casein possesses pro-inflammatory properties that can synergize, negatively affecting GI, endocrine, neurological, and cardiovascular systems. On the other hand, A2 milk (9, 10), which is free of A1 β-casein, has beneficial effects on human health and is easier to digest in lactose-intolerant individuals (11), making it a feasible alternative solution for individuals with pertinent GI disorders (12).

In ruminants, the rumen is the primary organ responsible for converting plant feeds into nutrients and energy (13, 14). According to research, microorganism derivatives, diet composition, and host metabolism influence rumen metabolite concentrations and colony structure, with all three factors collectively shaping the mechanisms underlying microbiota-host interactions (15). Rumen microbes have been established to be crucially involved in ruminant productivity and health (16). For instance, cows with mastitis exhibited significant alterations in inflammation-associated microbial communities and metabolite abundance in their rumen (17). Additionally, Zhang et al. (18) employed rumen fluid metabolomics to identify potential milk production biomarkers in high- and low-yielding cows. Biohydrogenation-linked rumen microbial populations were also associated with individual milk fat % in dairy cows (19). Although scholars both at home and abroad have extensively assessed A2 β-casein genotypes in cows, they mostly used milk or genetic tests (3, 20). Furthermore, to the best of our knowledge, no studies have characterized biomarkers and their roles in the rumen of A2-type β-casein dairy cows. Consequently, we explored the rumen microbiomes and metabolomes of different genotyped cows and examined the roles of characteristic metabolites and microorganisms in A2A2 genotyped cows using an integrated approach involving weighted gene co-expression network analysis (WGCNA) and Spearman correlation analysis.

Herein, 45 Holstein cows of three different β-casein genotypes [A1A1, A1A2, and A2A2 (*N* = 15)] from the Ningxia Nongken Helanshan dairy farm were included. Their rumen fluids were analyzed using metagenomics and metabolomics techniques. Metabolite clustering analysis and association analysis of characteristic metabolites with their characteristic microorganisms were performed using the WGCNA-Spearman integrated approach to further elucidate the contribution of metabolites to milk fat synthesis. Additionally, the regulatory role of AA on BMECs was explored using CCK-8, EdU, and qRT-PCR assays. Our analysis of the differences between rumen flora composition and metabolic pathways in cows of different β-casein genotypes could provide an essential reference for subsequent studies on the molecular genetic mechanisms of the characteristics of Holstein cows with the A2 pure genotype.

## Materials and methods

### Animals and experimental design

Holstein cow rumen fluids were collected from the Ningxia Nongken Helanshan dairy farm. The experimental cows were fed the same balanced total mixed ration (TMR) diet (Supplementary Table S1). Notably, the cows were previously typed using the competitive allele-specific PCR (KASP), and three genotypes were obtained: A1A1, A1A2, and A2A2 (21). For each genotype, 15 Holstein cows were selected in good condition and in their first lactation, in which the milk fat and protein content were similar across the three groups (Supplementary Table S2).

### Sample collection

Two hours after the morning feeding, the rumen contents were collected using a rumen fluid collection tube. Specifically, after inserting the rumen fluid collection tube, rumen vesicle contents were aspirated and collected under negative pressure. To avoid contamination with saliva, the first 150 mL of the collected rumen contents were discarded. Subsequently, 100 mL rumen content was collected and filtered using four sterile gauze layers, portioned, quickly frozen in liquid nitrogen, and stored in a −80°C refrigerator, awaiting further use. The Institutional Animal Care Committee of Ningxia University approved our experimental protocol (Approval Number: NXU-2024-065).

### Microbiota analysis

First, total DNA was extracted from the rumen fluid and purified using a DNA extraction kit (TruSeq Nano DNA LT Sample Preparation Kit, Illumina, United States), following the manufacturer’s instructions. Subsequently, DNA concentration and quality were assessed using 1.0% agarose gel electrophoresis and a NanoDrop spectrophotometer. Following that, purified and tested DNA samples underwent fragmentation and end repair using the Covaris S220 before attaching the Y-junctions to the sample ends. We then performed PCR amplification to recover the target fragments and create a library. Subsequently, the libraries were sequenced on the Illumina HiSeq 2000 platform at the Shanghai Ouyi Biomedical Technology Co., Ltd. Following that, the genes were filtered and quality-controlled using Trimmomatic (v0.36) and Bowtie2 (v2.2.9) before splicing the sequences using MEGAHIT (v1.1.2) software. The spliced contigs’ open reading frames (ORFs) were predicted using Prodigal (v2.6.3) software. Finally, clustering was performed using CDHIT (v4). After predicting the ORF of the spliced contig, we constructed the non-redundant gene set of the predicted genes using CDHIT (v4.5.7) software.

The obtained set of non-redundant genes was compared to the GeneBank non-redundant (NR) database of nucleic acid sequences^1^ using DIAMOND (v0.9.7) software. The sequences with an *e*-value ≤l × 10^−5^ were considered meaningful for obtaining species annotation information. Differences in α-diversity indices, including Shannon, Simpson, and ACE, were examined to detect the median, dispersion, maximum, minimum, and outliers of species diversity, yielding insights into rumen microbial diversity. The rumen fluid characteristic microorganisms of dairy cows across the three genotypes were screened using the Linear discriminant analysis Effect Size (LEfSe) approach based on the LDA >2 and *p* < 0.05 criteria. Finally, the predicted genes were integrated with the Kyoto Encyclopedia of Genes and Genomes (KEGG) database^2^ to obtain the gene function annotation information.

### Untargeted metabolomics

First, the stored sample was thawed slowly on ice before obtaining 1 mL from the SPE solid-phase column and precisely adding 3 mL methanol. After blow drying, the sample was further dried through nitrogen blowing using a nitrogen blowing instrument before adding 300 μL methanol-water mixture (V:V = 4:1, containing L-2-chlorophenylalanine, 4 μg/mL) to redissolve it. Subsequently, the sample was vortexed for 1 min, sonicated for 10 min in an ice-water bath, and incubated at −40°C for 30 min. The sample was then centrifuged at 12,000 rpm for 10 min at 4°C before aspirating 150 μL of the supernatant using a syringe, which was filtered through a 0.22 μm organic-phase pinhole membrane, transferred to a liquid chromatography-mass spectrometry (LC-MS) injection vial, and stored at −80°C, awaiting LC-MS analysis.

Metabolite detection was performed using a liquid-mass spectrometry platform comprising an ACQUITY UPLC I-Class plus ultrahigh-performance liquid chromatography-tandem system and a QE plus high-resolution mass spectrometer. The LC-MS instrument was equipped with a preset ACQUITY UPLC HSS T3 chromatography column (100 mm × 2.1 mm, 1.8 μm), operated at a flow rate of 0.35 mL/min and a temperature of 45°C. Mobile phase A consisted of water and 0.1% formic acid, whereas mobile phase B comprised 100% acetonitrile. Supplementary Table S3 shows the elution process of the mobile phases. Each sample (2 μL) was injected into an autosampler set at 4°C. The spray voltages for the positive and negative modes were set at 3.8 kV and 3.0 kV, respectively. Other parameters were the same for both the positive and negative modes (capillary temperature = 320°C; aux gas heater temperature = 350°C). The raw peaks were extracted, analyzed, and quantified using the LECO-Fiehn Rtx5 database, and normalization analyses were performed (22). To obtain precise qualitative and relative quantitative results, the peaks were compared to those in various databases such as mzCloud,^3^ mzVault, and MassList. Statistical analyses were performed using R v3.43, Python v276, and Cent vOS66 software.

MetaX, a metabolomics data processing software, was used to perform principal component analysis (PCA) and partial least squares discriminant analysis (PLS-DA). Statistical significance (*p*-value) was evaluated using one-way analysis of variance, and marker metabolite screening was aided by the variable importance for the projection (VIP) of the (O)PLS-DA model variables. Metabolites with VIP >1, *p* < 0.05, FC ≥2, or FC ≤0.5 were considered differential expressed. Annotation and metabolite pathway analysis was performed using metabolites obtained from the KEGG (see text footnote 2), HMDB,^4^ and LIPID MAPS^5^ databases.

### Metabolite co-expression module construction

To obtain precise qualitative and relative quantitative results, the peaks were compared to those in databases such as mzCloud (see text footnote 3), mzVault, and MassList. Statistical analyses were performed using R v3.43, Python v276, and CentOS66 software (23). For network construction, we used the soft threshold power (β) based on an *R*-value of 0.96. The smallest module comprised 35 genes (minimum module size = 35), and the merged module had a height of 0.25. Correlations between modules and cow milk fat % were determined to identify modules that affect milk fat, and metabolomics was used to enrich for metabolites within those modules.

### Statistical analysis

Univariate ANOVA (*t*-test) was used to assess statistical significance (*p*-value), with *p* < 0.05 and *p* < 0.01 indicating significant and highly significant differences, respectively. GraphPad Prism 8 was used to plot histograms. Dominant rumen flora (*R* > 0.6, *p* < 0.05) were correlated with milk fat-related metabolites using Spearman correlation analysis, and all significant correlation networks were visualized using Cytoscape (3.8.2). Receiver operating characteristic (ROC) curves were plotted, and the corresponding area under the curve (AUC) values were computed using the ROCR software package (24).

### Cell culture

Mammary epithelial cell lines from dairy cows were cultured and frozen in the preliminary phase of this experiment. Specifically, BMECs were grown in a DMEM/F12 growth medium supplemented with 10% fetal bovine serum (FBS) (Cell Max, Beijing, China) in a 5% CO_2_ and 37°C incubator. Passaging and culture treatments were performed at ~70–80% cell density.

### AA master mix configuration

To prepare AA mother liquor at a 10 mM concentration, 10 mg AA dry powder (Sigma, America) was first weighed and then transferred into a 5 mL centrifuge tube before adding 3.28 mL anhydrous ethanol to dissolve it at room temperature (RT). After thorough mixing, the solution was filtered to remove bacteria, dispensed into 200 μL centrifuge tubes, and stored at −20°C in a refrigerator in the dark for spare use. The experimental group received AA at final concentrations of 1, 5, and 10 μM, whereas the control group (NC) received anhydrous ethanol.

### CCK-8 cell viability and cell proliferation EdU assays

First, cells were inoculated into 96-well plates, and the optimal concentrations from the AA treatment and experimental groups were selected for the EdU assay. Following the instructions in the EdU assay kit (Beyotime, Shanghai, China), the 2xEdU working solution was prepared in equal volumes and added into petri dishes after seeding cells in optimal growth conditions into 6-well plates. The cells were then observed under an inverted fluorescence microscope DMi8 (Leica, Germany) and counted using ImageJ software.

### TG content determination

The treated cells were tested for TG content using the cell-specific high-fat sample TG enzymatic assay kit (E1025, Prilosec, Beijing, China). Based on the reagent instructions, the lysed supernatant was added to the prepared working solution, and the reaction was conducted at 37°C for 15 min. Each tube’s optical density (OD) value was detected at 550 nm, and the TG content was adjusted based on protein concentration per mg.

### RT-qPCR-related gene expression detection

Total RNA was extracted using TRizol reagent (Invitrogen, Thermo Fisher, United States) and then reverse-transcribed into complementary DNA (cDNA) using Prime Script RT Reagent Kit (Takara, Dalian, China). Following the manufacturer’s instructions, SYBR Premix Ex Taq^™^ II (Takara, Dalian, China) was used to extract RNA from the cells for RT-qPCR on the Bio-Rad CFX96 Touch^™^ Real-Time PCR Detection System (Bio-Rad, Hercules, CA, United States). The primers used were designed using the Primer Premier 5.0 system (Supplementary Table S4). The 2^−ΔΔCt^ technique was used to analyze the relative mRNA expression in different treatment groups. Gene expression was normalized to *GAPDH*, and all results were subjected to ANOVA using SAS software (version 9.2, SAS Institute, Cary, NC). Three replicates were set up for each gene, and results or differences with *p* < 0.05 and *p* < 0.01 were considered significant and highly significant, respectively.

## Results

### Structural analysis of microbial communities

**Figure 1:**
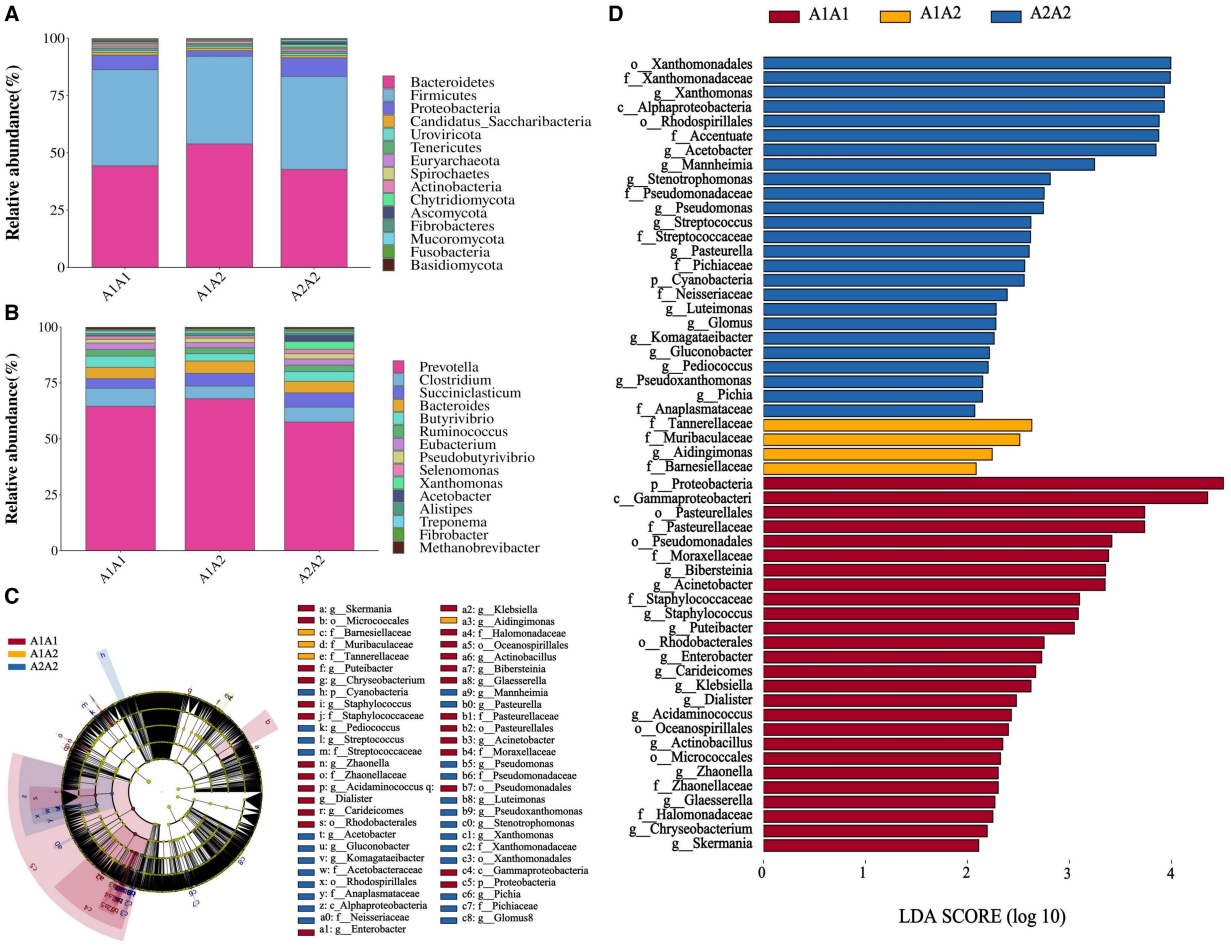
Structural analysis of rumen microbial communities. **(A)** The relative abundance of the 15 most abundant bacteria at the phylum level. **(B)** The relative abundance of the 15 most abundant bacteria at the genus level. **(C)** Branch diagram of LEfSe analysis of the three groups. **(D)** Histogram showing the distribution of LDA values among the three groups; higher LDA scores indicate greater importance of the bacteria.

Herein, rumen fluid samples from 45 Holstein cows were subjected to macro genome sequencing. According to the results, the samples’ clean reads and contig N50 statistics were distributed in the 10.01–16.63 G and 299–475 bp ranges, respectively, and the number of ORFs in the constructed gene catalog (non-redundant genes) after de-redundancy was 15,361,808 (Supplementary Table S5). Furthermore, sample size significance was determined using core-pan gene dilution curve analysis. According to the results, the number of subjects selected for the study (*n* = 45) was sufficient (Supplementary Figure S1A). A comparison of specific genes across the three groups (A1A1, A1A2, and A2A2) revealed that their proportions were 2.23, 3.29, and 2.58%, respectively (Supplementary Figure S1B). Additionally, we determined the rumen flora α diversity indices based on species abundance (Supplementary Table S6). According to the results, the three groups showed no significant differences in the three α diversity indices (Shannon, Simpson, and ACE) (*p* > 0.05). Conversely, PCA revealed microbial β diversity differences in the rumen fluid of the dairy cows with different genotypes (Supplementary Figure S1C). Furthermore, Analysis of Similarities (ANOSIM) revealed that the differences among the three groups were significantly greater than those within each group, indicating meaningful subgroup distinctions (*R* = 0.078, *p* < 0.05; Supplementary Figure S1D). The top 15 most abundant phyla and genera among the 45 cows were plotted using a species relative abundance bar chart (Figures 1A,B). The dominant phylum- and genus-level microorganisms were *p_Bacteroidetes* and *p_Firmicutes* and *g_Prevotella* and *g_Clostridium*, respectively. The potential biomarkers in the rumen of the three different dairy cow genotypes were further examined through LEfSe analysis. According to the results, 55 characterized microorganisms were enriched in A2A2 cows (Supplementary Table S7), of which the key biomarker genera were *g_Stenotrophomonas*, *g_Fusobacterium*, *g_Mannheimia*, *g_Acetobacter*, *g_Xanthomonas*, *g_Pichia*, *g_Pseudoxanthomonas*, *g_Pediococcus*, *g_Gluconobacter*, *g_Komagataeibacter*, *g_Glomus*, *g_Luteimonas*, *g_Pasteurella*, and *g_Streptococcus* (Figures 1C,D).

### Analysis of marker bacteria in the rumen of dairy cows across the three different genotypes

**Figure 2:**
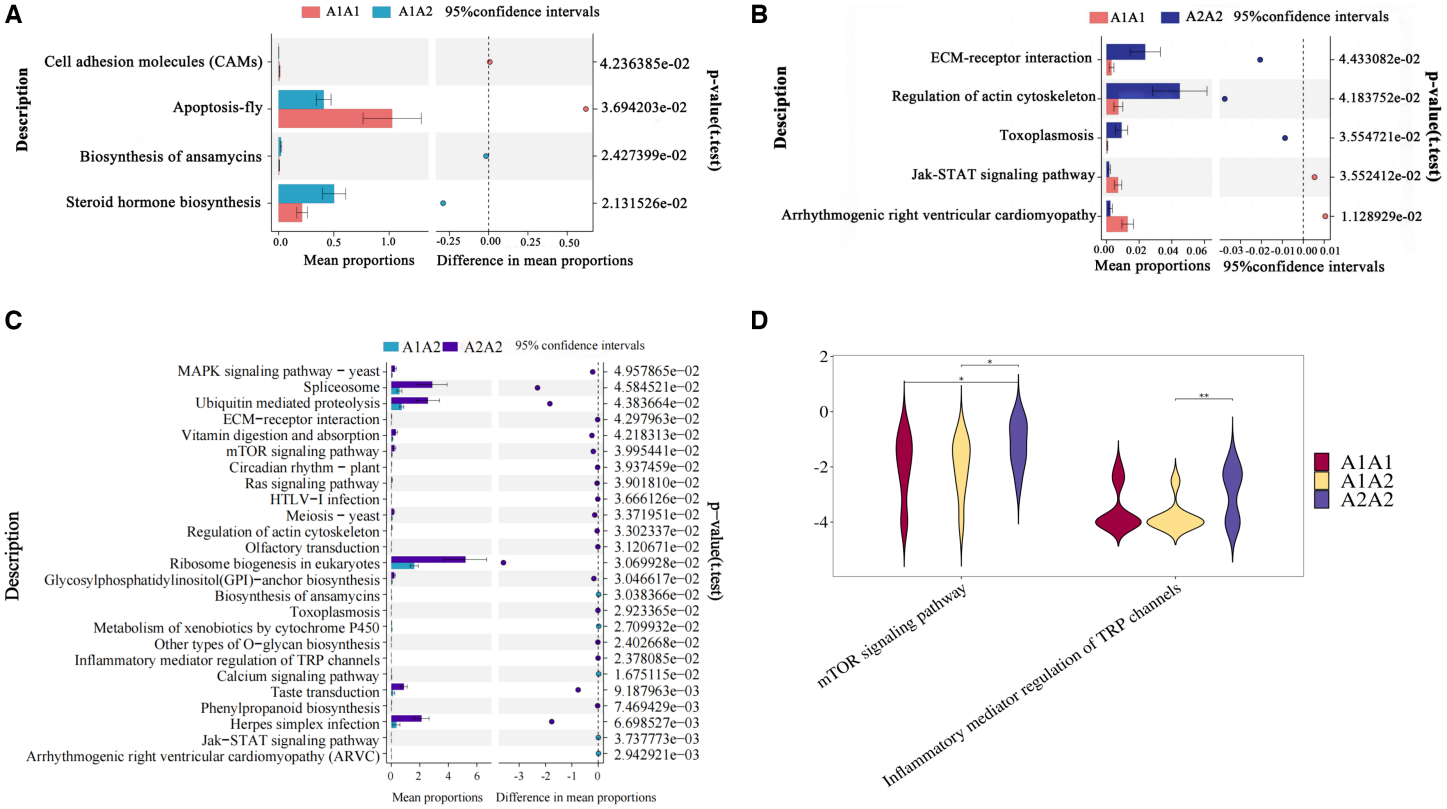
Functional analysis of rumen microorganisms in dairy cows. **(A–C)** The proportion of abundance of different KEGG functional entries between groups at the functional level, with the proportion of differences within the 95% confidence interval shown in the middle, and the rightmost value is the *p*-value, with *p* < 0.05 indicating a significant difference. **(D)** Violin plot of the key KEGG functional entries in cows of the A2A2 genotype.

Differential KEGG functional enrichment through STAMP analysis revealed that arrhythmogenic right ventricular cardiomyopathy (ARVC) and jak-STAT signaling pathway, among others, were the functions enriched in A1A1 and A1A2 genotyped cows. On the other hand, TRP channels’ inflammatory mediator regulation and the mTOR signaling pathway, among others, were the functions enriched in A2A2 genotyped cows (Figure 2).

### Analysis of metabolomics results

**Figure 3:**
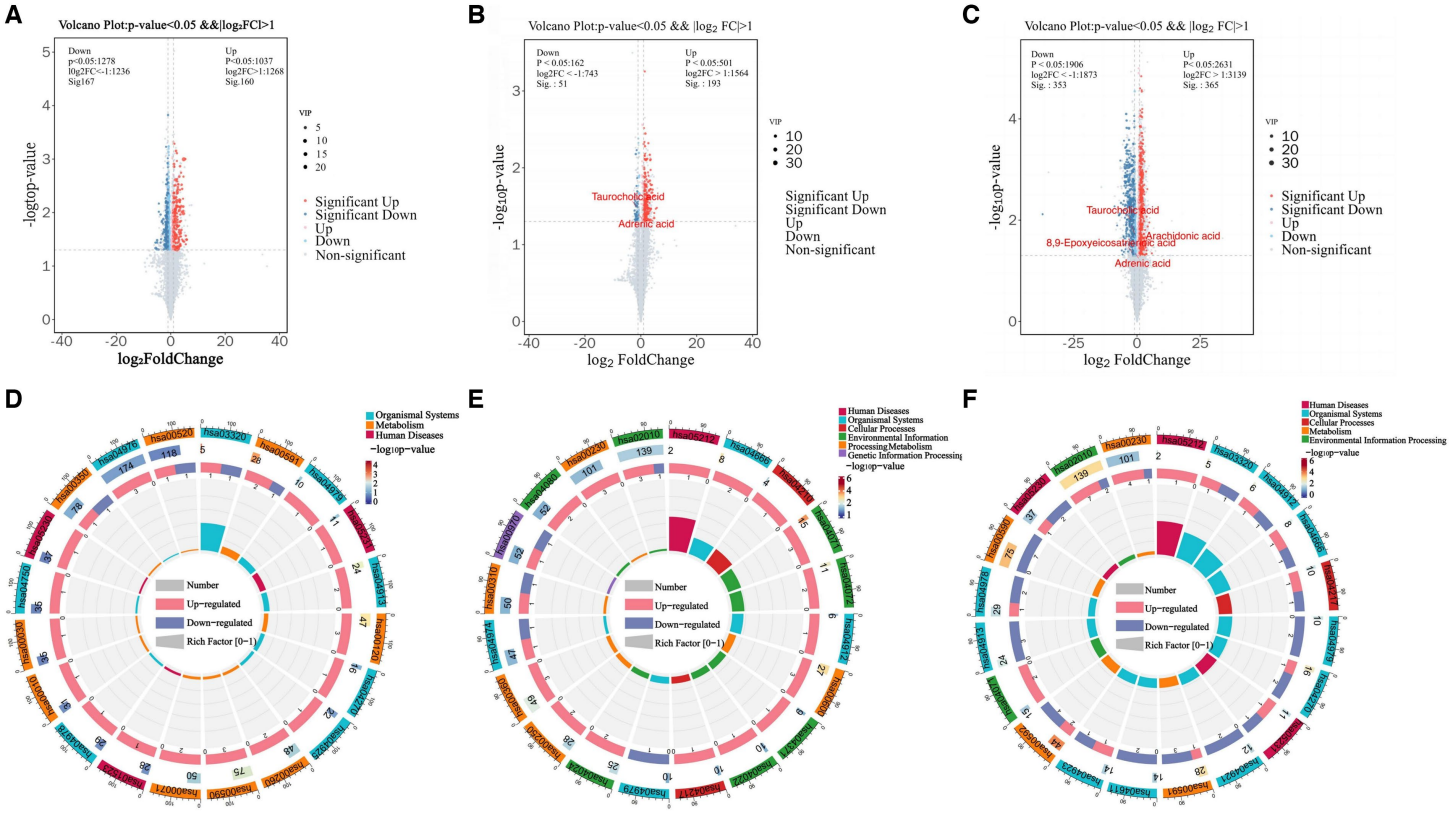
Rumen metabolome analysis. **(A)** Volcano plots of differential metabolites between A1A1 and A1A2 groups, **(B)** A1A1 and A2A2, **(C)** A1A2 and A2A2. **(D)** The circle plots of KEGG enrichment analysis between groups A1A1 and A1A2, **(E)** A1A1 and A2A2, **(F)** A1A2 and A2A2. There are 4 circles from the outside to the inside: the first circle: the classification of enrichment, the outside of the circle is the scale of the number of metabolites, different colors represent different classifications; the second circle: the number of the classification in the background metabolism and the *p*-value. The more metabolites the longer the bar is, the smaller the value the redder the color is, and the bigger the value the bluer the color is; the third circle: the bar of the proportion of the metabolism in the up and down-regulated metabolism, light red represents the proportion of the metabolism in the up-regulated metabolism, light blue represents the proportion of metabolism in the down-regulated metabolism; the specific values are shown below; and the specific values are presented below. Proportions, specific values are shown below; fourth circle: RichFactor values for each category, each cell of the background auxiliary line represents 0.2.

Differentially expressed metabolites (DEMs) in the rumens of Holstein cows across the three different genotypes were detected using UPLC-MS metabolomics technology. A total ion chromatogram (TIC) overlap plot was obtained via superimposition of the mass spectra of the QC samples from the positive and negative ion detection modes on the TIC data (Supplementary Figures S2A,B), revealing that the peaks’ response intensities and retention times overlapped and had stable baseline values. These findings confirmed the reliability of the experimental data. According to the PCA and PLS-DA results, metabolites in the three dairy cow genotypes showed inter- and intra-group disparities (Supplementary Figures S2C,D). Furthermore, the OPLS-DA score plots of metabolites showed significant differences across the rumen metabolite groups of the three dairy cow genotypes, and the permutation test revealed that all OPLS-DA models were reliable and did not overfit (Supplementary Figures S3A–F). Venn diagrams of DEMs and differential metabolic pathways among the three groups showed that the two comparison groups overlapped significantly (Supplementary Figures S2E,F). Statistical analysis of mass spectrometry-identified metabolites revealed 691 DEMs in the rumen fluid of the A1A1 and A1A2 dairy cow groups (Figure 3A and Supplementary Table S8) and 283 DEMs in the rumen fluid of the A1A1 and A2A2 dairy cow groups (Figure 3B and Supplementary Table S9). Furthermore, the analysis of the rumen fluid of the A1A2 and A2A2 dairy cow groups revealed 1,025 DEMs (Figure 3C and Supplementary Table S10). The top 50 DEMs were then analyzed for their respective enrichments (Supplementary Figures S4A–C). The substances enriched in the A2A2 group were arachidonic acid (AA), adrenic acid (AdA), taurocholic acid (TCA), and glycocholic acid (GCA). To better understand differential metabolite enrichment across the three groups, the top 20 metabolic pathways of differential metabolites were identified (Figures 3D–F), including those related to cholesterol metabolism, vascular smooth muscle contraction, ovarian steroidogenesis, primary bile acid biosynthesis, the GnRH signaling pathway, AA metabolism, Fc gamma R-mediated phagocytosis, and necroptosis.

### WGCNA

**Figure 4:**
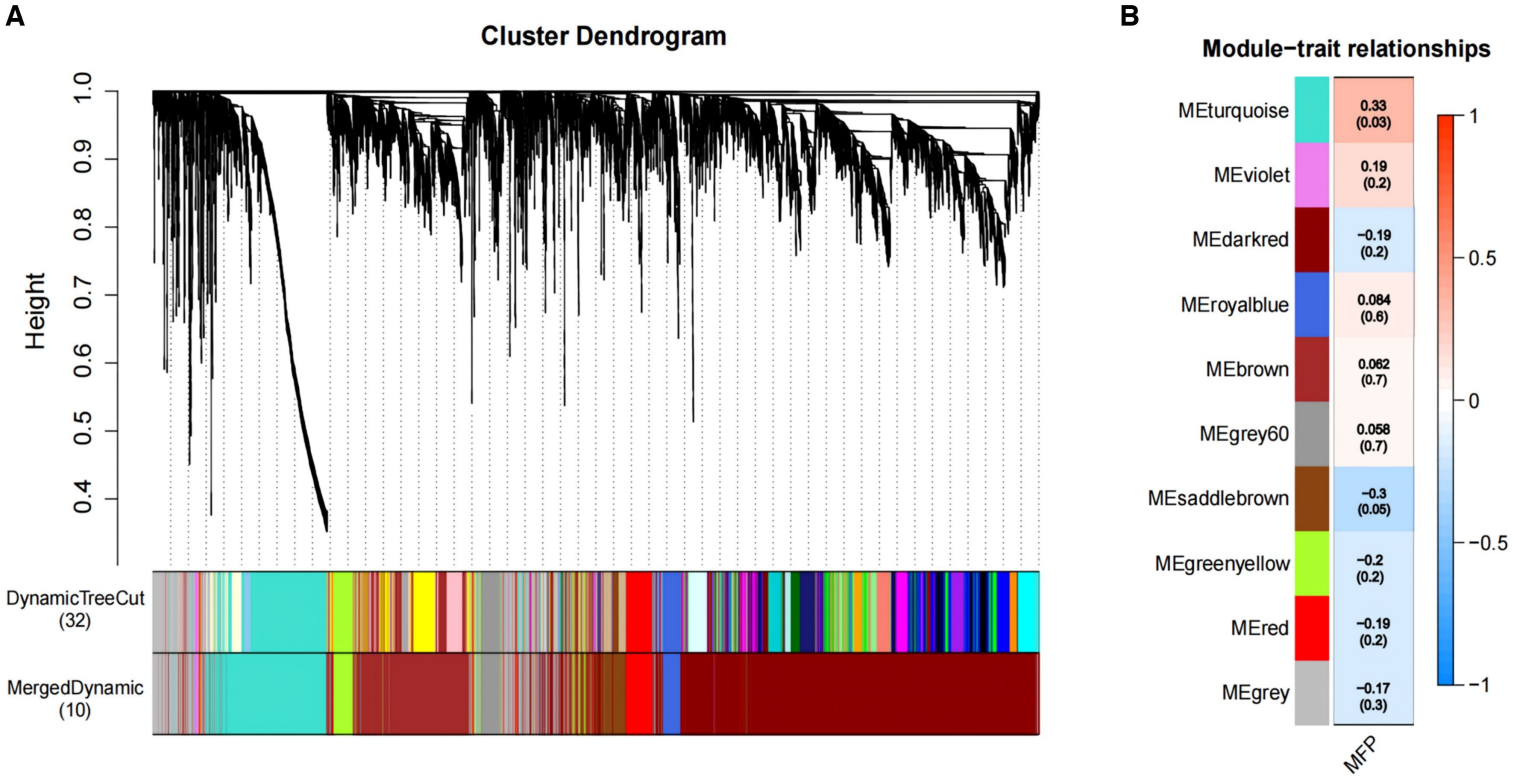
Association of metabolites with MFP based on WGCNA. **(A)** The clustering dendrogram of the average network adjacency for the identification of metabolite co-expression modules. **(B)** Heatmap of the correlation of module trait genes with MFP. Each row corresponds to a trait module, and the each column represents a trait. The plot is color-coded by correlation according to the color legend, and each module contains the corresponding correlation and *p*-value. MFP, milk fat percentage.

We constructed co-expression networks between the identified metabolites and milk fat % using WGCNA to better understand the relationship between metabolites and milk fat % in Holstein cows. Ten co-expression modules were identified after merging modules with similar characteristics (Figure 4A). Furthermore, the results showed that MEturquoise correlated positively with milk fat content (Figure 4B) and 1,308 metabolites from this module were selected for further analysis.

### Metabolic pathway analysis

**Figure 5:**
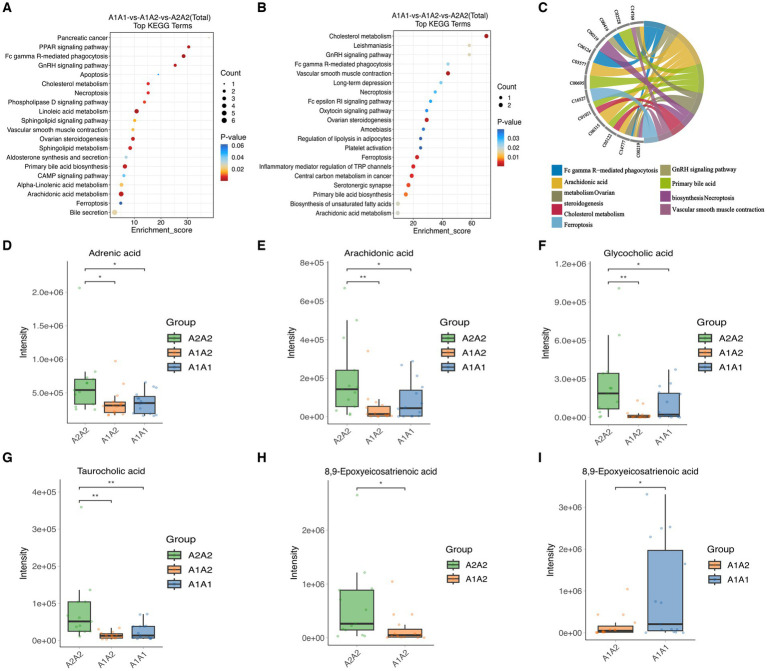
Differential metabolites and differential metabolic pathway analysis. **(A)** Top 20 differential metabolic pathways enriched in differential metabolites among the three groups (MEturquoise). **(B)** Top 20 differential metabolic pathways enriched in differential metabolites among the three groups; color gradient and circle size indicate the significance of pathways sorted by *p*-value (red: higher *p*-value, blue: lower *p*-value) and pathway impact scores (the larger the circle, the higher the impact score), respectively. **(C)** Nine co-enrichment pathways corresponding to metabolite chordograms. **(D–I)** Levels of adrenic acid, AA, glycocholic acid, 8,9-EET, and taurocholic acid in the rumen of dairy cows of three genotypes.

MEturquoise metabolites were significantly positively correlated with milk fat % (cor = 0.6, *p* = 7.7 × 10^−48^) (Supplementary Figure S5A). Notably, enrichment analysis of the 1,308 MEturquoise metabolites yielded 35 DEMs (VIP >1.00, *p* < 0.05), including AA, GCA, and TCA (Supplementary Figure S5C). All 35 DEMs, as well as those between the three groups, were subjected to metabolic pathway enrichment (661 metabolites, VIP >1, *p* < 0.05) (Figures 5A,B). According to the results, pathways such as cholesterol metabolism, vascular smooth muscle contraction, ovarian steroidogenesis, ferroptosis, primary bile acid biosynthesis, the GnRH signaling pathway, AA metabolism, Fc gamma R-mediated phagocytosis, and necroptosis were co-enriched (Supplementary Figure S5B). Furthermore, the metabolites included in the co-enrichment pathway were AA, AdA, GCA, TCA, and 8,9-Epoxyeicosatrienoic Acid (8,9-EET) (Supplementary Table S11). Specifically, AA, AdA, GCA, and TCA were significantly enriched in A2A2 cows, whereas 8,9-EET was significantly enriched in both A2A2 and A1A1 cows (*p* < 0.05) (Figures 5D–I).

### Correlation analysis of key metabolites in rumen milk fat with characteristic flora in A2A2 genotyped dairy cows

**Figure 6:**
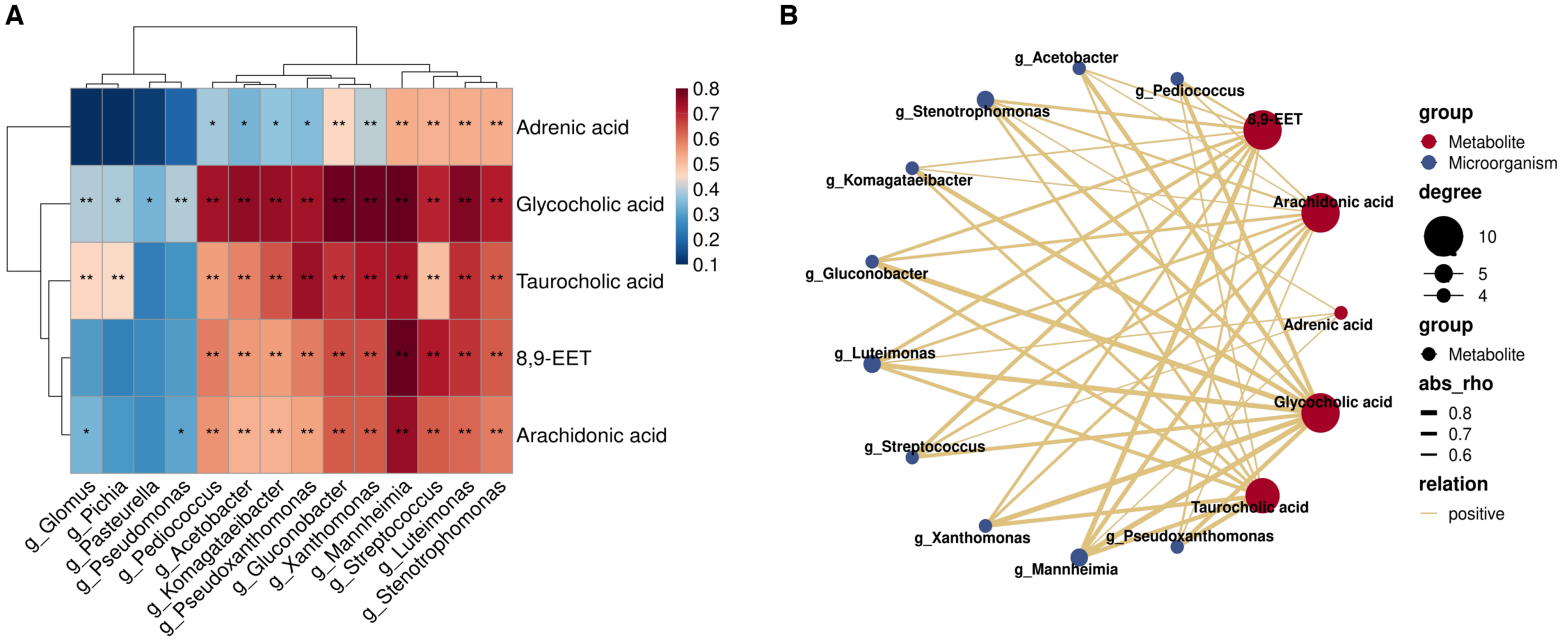
Correlation analysis of key flora and metabolites for differences among groups. **(A)** Heatmap of the Spearman correlation coefficient matrix between key rumen metabolites for milk fat synthesis and key microorganisms of group A2A2; ** represents a significant *p* < 0.01, * represents a significant *p* < 0.05. **(B)** A correlation network map; the colors of the lines represent positive and negative values of the correlation coefficients between the two (blue for negative correlation, red for positive correlation, only correlation with |*R*| > 0.6 and *p* < 0.01), and the thickness of the lines is directly proportional to the absolute value of the correlation coefficients; degree: centrality, the number of other nodes to which each node is connected, gradient according to the size of the centrality value.

Herein, we employed Spearman’s correlation analysis to examine the relationship between characteristic genera (LDA >2; *p* < 0.05) and key differential rumen metabolites of milk fat in the rumen fluid of A2A2 dairy cows in order to characterize the relationship between the rumen flora and key metabolites of milk fat (Figure 6A). According to the results, some of the genera and metabolites correlated significantly and strongly (|*R*| > 0.6, *p* < 0.01) (Figure 6B). Among them, *g_Acetobacter*, *g_Pseudoxanthomonas*, *g_Streptococcus*, *g_Pediococcus*, *g_Mannheimia*, *g_Stenotrophomonas*, *g_Komagataeibacter*, *g_Gluconobacter*, and *g_Luteimonas* correlated significantly positively with both primary bile acid biosynthesis (GCA and TCA) and AA metabolism (AA and 8,9-EET). It has been established that AA regulates milk lipid synthesis and secretion (25). In this regard, it is noteworthy that biomarker prediction using ROC curves to identify significantly enriched characteristic metabolites and flora in A2A2 cows revealed that the model had a good prognostic effect, with AUC values for AA of 0.713 in groups A1A1 vs. A1A2 and 0.846 in groups A1A2 vs. A1A2 (Supplementary Figure S6). We will focus more on AA in the subsequent sections.

### Effects of the candidate marker metabolite AA on BMEC proliferation

**Figure 7:**
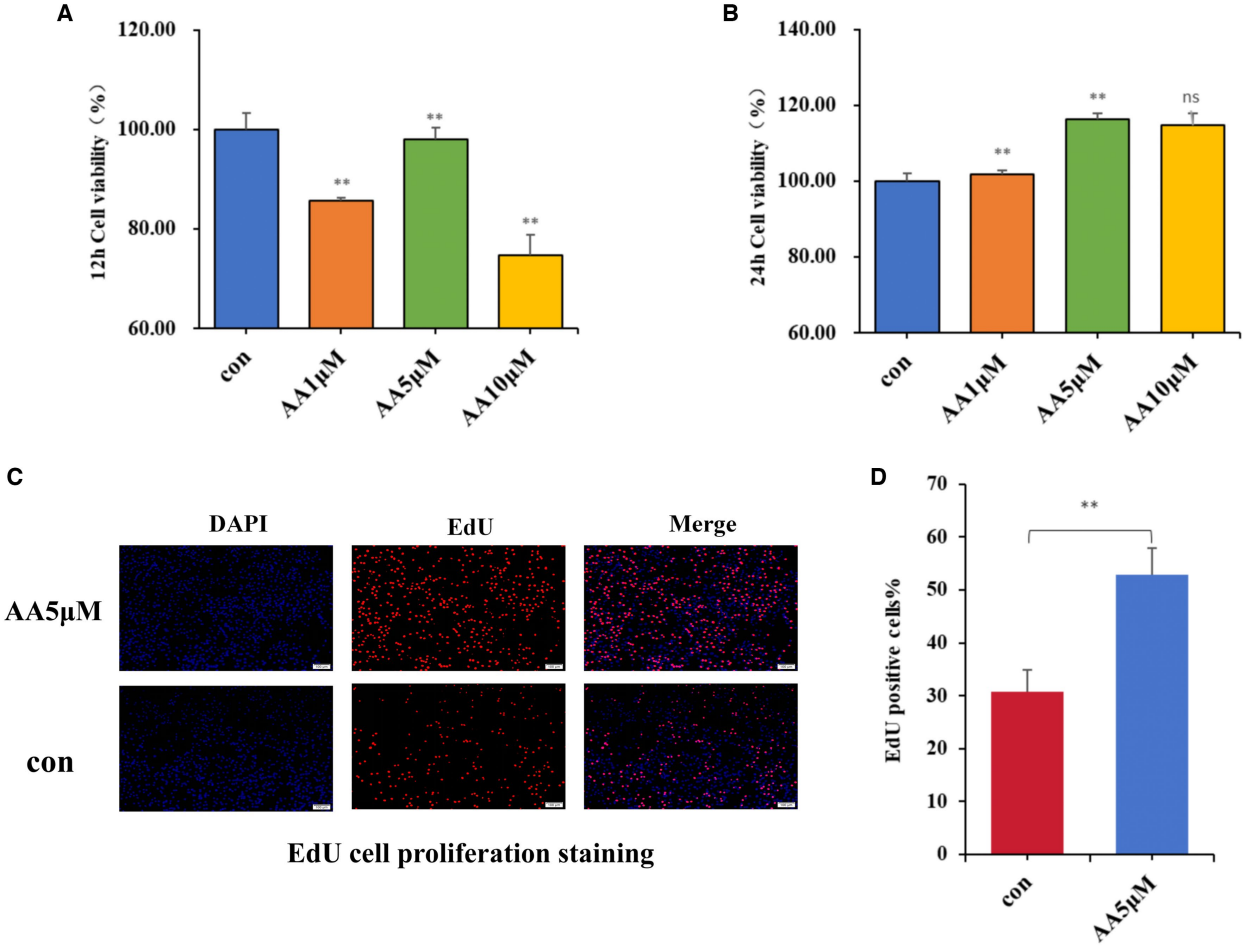
The effects of AA on cell proliferation. **(A,B)** Effects of different concentrations of AA on the viability of BMECs at 12 h and 24 h. **(C)** EdU cell proliferation staining images. **(D)** EdU proliferating cell counting analysis.

After culturing BMECs *in vitro* for 12 and 24 h, different AA concentrations were added to the culture medium, and their effects on BMEC viability were evaluated using the CCK-8 assay. According to the results, 5 μM AA was the optimal concentration, with the cells reaching the highest viability after 24 h (Figures 7A,B). Furthermore, the EdU results showed that the 5 μM AA-treated group exhibited enhanced BMEC proliferation after 24 h of incubation (Figures 7C,D).

### Effects of the candidate marker metabolite AA on milk fat synthesis

**Figure 8:**
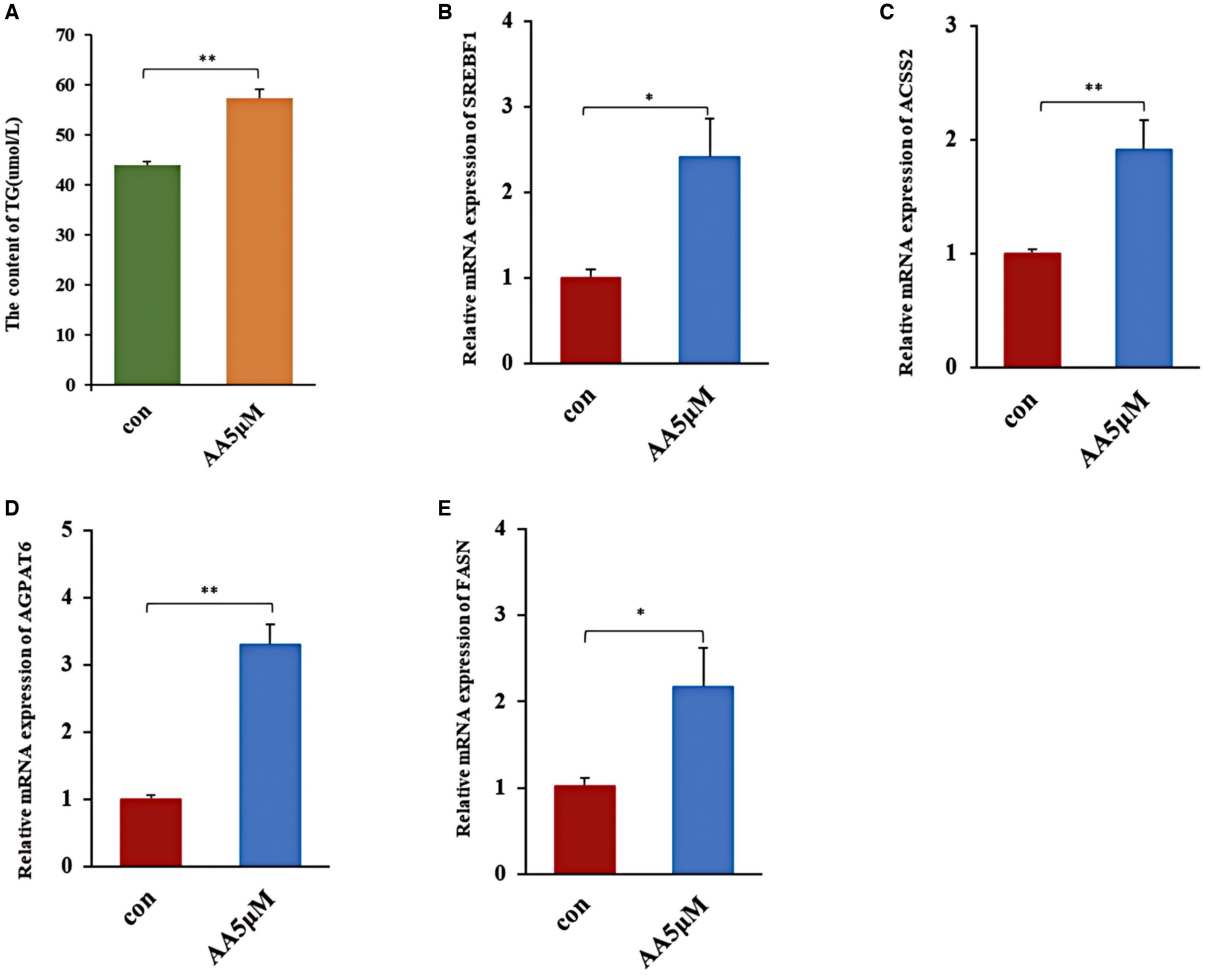
The effect of AA on milk fat synthesis. **(A)** TG content; **(B–E)** relative expression of milk lipid synthesis marker genes *SREBF1*, *ACSS2*, *AGPAT6* and *FASN*. ** In the figure represents a significance *p* < 0.01, and * in the figure represents a significance *p* < 0.05.

We treated BMECs with 5 μM AA for 24 h to further determine whether AA influences the cells’ milk lipid synthesis. We then assessed TG concentration and expression levels of milk lipid marker genes. According to the results, the 5 μM concentration AA-treated group exhibited a significantly elevated TG concentration (*p* < 0.01) (Figure 8A), as well as the upregulation of lactolipid marker genes, including SREBF1, *ACSS2*, *AGPAT6*, and *FASN* (Figures 8B–E).

## Discussion

A triad of flora, metabolites, and organismal immunity has been established to regulate the internal rumen environment, significantly impacting dairy cow health and performance (15). In the rumen, flora digest polysaccharides from feeds into Short-Chain Fatty Acids (SCFA) such as acetate, butyrate, and propionate, contributing up to 70% of animals’ total energy intake (14). Metabolites are important markers of biochemical reactions in the rumen microecosystem and are sensitive to changes in rumen microbiology (26). According to research, β-casein is essential for individual cow health and lactation performance (27, 28), a phenomenon consistent with our findings, which demonstrated that A2A2 genotyped cows exhibited a higher milk fat % than the other two genotyped groups. Furthermore, A1β-casein and A2β-casein can impact colony fermentation, and the rumen flora structure could affect colony metabolites and milk fat synthesis processes (29, 30). Nonetheless, whether rumen flora and metabolites differ in β-casein A1A1, A1A2, and A2A2 genotyped cows remained unclear. Consequently, we screened the characteristic metabolites related to milk fat synthesis in A2A2 genotyped cows using an integrated metabolomics and WGCNA approach. We then examined the rumen characteristic flora and functions in cows with different β-casein genotypes using metagenomics technology. Finally, Spearman correlation analysis was employed to determine the relationships between characteristic flora and metabolites of A2A2 genotyped cows.

Our findings revealed that AA, AdA, GCA, and TCA were significantly upregulated in A2A2 cows (*p* < 0.05). According to research, AdA, as a substance downstream of AA, has anti-inflammatory effects (31, 32). On the other hand, TCA and GCA are mainly enriched in the primary bile acid synthesis and cholesterol metabolism pathways, which are critically involved in lipid homeostasis and inflammation regulation (33, 34). Furthermore, TCA can inhibit the production of inflammatory mediators such as nitric oxide (NO), prostaglandin E2 (PGE2), and histamine, exerting anti-inflammatory effects (35). Additionally, TCA can regulate *ACACA*, *FASN*, *AACS*, and *LPL* expression, potentially promoting adipogenesis. On the other hand, GCA has been established to lower the serum levels of NO and Leukotriene B4 (LTB4), as well as PGE2 levels in inflammatory tissues, exerting an anti-inflammatory effect (36). Notably, AA is enriched in the AA metabolic pathway (part of the lipid metabolic pathway) and is closely related to lipid synthesis (37). According to research, AA can be converted to PGE2 and LTB4 via the cyclooxygenase pathway, inhibiting inflammatory cell migration and activation, thus exerting anti-inflammatory effects (38, 39). Furthermore, as an ω-6 Polyunsaturated Fatty Acid (PUFA), AA regulates milk lipid synthesis and secretion via *PPARγ* activity modulation (25). Research has also shown that AA can act via a specific G Protein-Coupled Receptor (*GPR120*) (40), and *GPR120* activators can promote milk fat synthesis through *SREBP1* and *FASN* upregulation (41). Herein, the AA-treated group exhibited *SREBF1* and *FASN* upregulation. Similarly, *AGPAT6* and *ACSS2*, key genes involved in fatty acid and TG synthesis regulation (42–44), were upregulated in the AA-treated group, resulting in enhanced TG synthesis. Based on these findings, we deduced that AA, a characteristic metabolite in the rumen of A2A2 genotyped cows, promotes milk fat synthesis. We further hypothesized that the higher milk fat % and anti-inflammatory effect of “A2 milk” on the GI tract of A2A2 genotyped cows may be related to AA, AdA, GCA, and TCA enrichment.

Rumen microbiota composition has been established to significantly impact milk production and composition in dairy cows (29). Herein, consistent with previous research (45, 46), the dominant phylum- and genus-level microorganisms in the flora content were *p_Bacteroidetes* and *g_Prevotella*, respectively. At the genus level, several significantly differentially expressed species were identified in the rumen of A2A2 cows, primarily belonging to genera *g_Pseudomonas*, *g_Acetobacter*, *g_Streptococcus*, and *g_Pediococcus*. According to research, *g_Pseudomonas* secrete lipase, which breaks down lipids in feeds into free fatty acids (FFAs), promoting saturated fatty acid accumulation in meat and milk (47, 48). On the other hand, *g_Acetobacter* oxidizes sugars to produce acetate, the precursor of milk fat synthesis, thus facilitating milk fat synthesis in the mammary glands (49). Furthermore, Edward et al. (48) discovered that *g_Acetobacter* can promote milk fat synthesis. Based on these findings, we hypothesized that milk fat synthesis in A2A2 genotypes cows could be linked to *g_Acetobacter* and *g_Pseudomonas* content. Moreover, *g_Streptococcus* and *g_Pediococcus* are lactic acid bacteria that produce lactic acid, which can be used as a substrate for secondary fermentation to produce precursors for milk fat synthesis: acetate, propionate, and butyrate (50, 51). In addition to increasing energy conversion efficiency to milk fat through its involvement in amino acid biosynthesis and energy substrate metabolism, *g_Streptococcus* has also been positively associated with serum bile acid levels (52, 53). Furthermore, not only is *g_Pediococcus* positively correlated with bile acid content, but its *Pediococcus pentosaceus strain KID7* can regulate bile acid regulation via the bile salt hydrolase BSH (54–56). It is also noteworthy that bile acids promote fatty acid transport and absorption in the body (57). We also found that *g_Streptococcus* and *g_Pediococcus* were significantly positively correlated (*p* < 0.01) with primary bile acids (GCA and TCA). These findings collectively suggest that *g_Streptococcus* and *g_Pediococcus* may modulate the synthesis of GCA and TCA, among other bile acids, potentially impacting milk fat synthesis. However, additional research will be required to verify the specific contribution of these genera in milk fat synthesis.

Functional enrichment results in the KEGG analysis for the colony revealed that the JAK-ATAT pathway was enriched in cows containing β-casein A1. This pathway has been implicated in the occurrence of cardiovascular disease, diabetes mellitus, inflammation, and immune regulation (58, 59). Type A1 β-casein has negative effects on gastrointestinal, endocrine, neurological, and cardiovascular systems by promoting inflammation via the JAK-ATAT pathway (9, 10). A2A2 genotype cows The genes involved in inflammatory mediator-regulated and mTOR signaling pathways of the TRP channel, which inhibit inflammation and milk fat synthesis, respectively (60, 61). The high rate of milk fat in the A2A2 genotyped cows and the suppressive effect of “A2 milk” on the intestinal inflammation may contribute to this effect.

Taken together, these results suggest that the rumen fluid characteristic flora and metabolites of A2A2 genotyped dairy cows are involved in milk fat synthesis and inflammation inhibition. Studies have demonstrated that the mechanisms and pathways of milk fat synthesis are complex, and are not were understood. In this study, significant correlations were observed between the rumen characteristic flora (*g_Streptococcus*, *g_Pediococcus*, *g_Acetobacter*, and *g_Pseudomona*s) and metabolites involved in milk fat rate (AA, adrenic acid, taurocholic acid, and glycocholic acid) of the A2A2 genotypic dairy cows. Furthermore, we found that the characteristic metabolite AA enhances milk fat synthesis. However, this study only screened rumen characteristic flora and metabolites without conducting a joint analysis with serum and milk metabolites. In future, the characteristic metabolites in serum and milk of A2A2-type cows need to be investigated to identify biomarkers in rumen fluid and understand the mechanisms of milk lipid synthesis in A2A2-type cows.

## Conclusion

In conclusion, cows with the A2A2 genotype in herds with similar body condition exhibited higher milk fat rates, with rumen signature metabolites including AA, adrenic acid, taurocholic acid and glycocholic acid, and signature genera including *g_Acetobacter*, *g_Pseudoxanthomonas*, *g_Streptococcus* and *g_Pediococcus*. Among them, the signature metabolite AA promotes the synthesis of milk lipids in BMECs, suggesting that they may serve as potential biomarkers in the A2A2 genotyped cows.

## Data Availability

The raw sequence data reported in this paper have been deposited in the Genome Sequence Archive (Genomics, Proteomics & Bioinformatics 2021) in National Genomics Data Center (Nucleic Acids Res 2021), China National Center for Bioinformation/Beijing institute of Genomics, Chinese Academy of Sciences (GSA: OMIX007432, GSA: OMIX007436) that are publicly accessible at https://ngdc.cncb.ac.cn/gsa.

## Footnotes

^1^ https://www.ncbi.nlm.nih.gov/guide/taxonomy/

^2^ http://www.genome.jp/kegg/pathway.html

^3^ https://www.mzcloud.org/

^4^ https://hmdb.ca/metabolites

^5^ http://www.lipidmaps.org/
