# Corrigendum: Integrated metagenomics and metabolomics analyses revealed biomarkers in β-casein A2A2-type cows

**DOI:** 10.3389/fvets.2024.1518338

**Published:** 2024-11-15

**Authors:** Jinyan Zhao, Chuanchuan Wang, Jiahuan Hu, Ruoshuang Ma, Baojun Yu, Wei Zhao, Hua Wang, Yaling Gu, Juan Zhang

**Affiliations:** ^1^Key Laboratory of Molecular Cell Breeding for Ruminants, Yinchuan, China; ^2^Ningxia University College of Animal Science and Technology, Yinchuan, China

**Keywords:** Holstein dairy cows, beta-casein, A2A2, milk fat percentage, metabolomics, metagenomics

In the published article, there was an error in the author list, and author Chuanchuan Wang's contribution as first author was omitted. The corrected author list appears below.

Jinyan Zhao^1,2†^, Chuanchuan Wang^1,2†^, Jiahuan Hu^1,2^, Ruoshuang Ma^1,2^, Baojun Yu^1,2^, Wei Zhao^1,2^, Hua Wang^1,2^, Yaling Gu^1,2^ and Juan Zhang^1,2*^

^†^These authors share first authorship

The authors apologize for this error and state that this does not change the scientific conclusions of the article in any way. The original article has been updated.

