# Supplementary material for: Integrated metagenomics and metabolomics analyses revealed biomarkers in β-casein A2A2-type cows
Source: Front Vet Sci. 2024 Oct 1;11:1438717. doi: 10.3389/fvets.2024.1438717 (PMC11475472; doi:10.3389/fvets.2024.1438717)
Supplement: Supplementary file 1 [file Data_Sheet_1.pdf]

Table S1 TMR ingredient and nutrient component (% of DM)

Item	Content	Nutrient level	Content
Alfalfa	16. 230	Dry matter/kg	17. 280
Corn silage	51. 320	NEL/ (MJ·kg-1)	7. 770
Tablet corn	10. 820	CP	18. 310
Soybeanmeal	10. 820	NDF	35. 840
Cottonseedmeal	5. 400	DF	21. 850
10% premix	5. 410	Fat	2. 590
Total	100.000	Ca	0. 520

Note: Each kilogram of premix contains: VA 800 000 IU, VD 200 000 IU, VE 4 000 mg, Cu 1200 mg, Fe 6 000 mg, Mn 4 000 mg, Zn 4 000 mg, I 40 mg, Co 40 mg, Se 32 mg. Note: Per kilogram of the premix contained the following: VA 800 000IU, VD 200 000 IU, VE 4 000 mg, Cu 1 200 mg, Fe 6 000 mg, Mn 4 000 mg, Zn 4 000 mg, I40 mg, Co 40 mg, Se 32 mg.

Table S2 The basic information and grouping of experimental cows (n = 45)

	Group			*P*-value
	A1A1	A1A2	A2A2	
Milk fat percentages	3.860±0.220	3.870±0.160	3.920±0.160	0.640
Milk protein percentage	3.270±0.100	3.320±0.130	3.360±0.200	0.310
Somatic cell count (10 000/ml)	46.270±65.410	15.930±17.910	43.930±69.710	0.290
somatic cell score	1.210±1.260	0.560±0.690	1.410±1.420	0.260
305 days of milk production	12457.070±1520.500	13270.400±1440.770	12629.400±1231.230	0.280
Daily milk production	38.790±4.130	40.740±4.940	42.180±10.750	0.550

Table S3 The mobile phase elution procedue

Time (min)	Flow rate (mL/min)	A (%)	B (%)
0.000	0.350	95	5
2.000	0.350	95	5
4.000	0.350	70	30
8.000	0.350	50	50
10.000	0.350	20	80
15.000	0.350	0	100
15.000	0.350	0	100
15.100	0.350	95	5
16.000	0.350	95	5

Table S4 List of primer information

Genes	Primer sequence (5’-3’)	Product length /bp	Annealing temperature /°C
GAPDH-F	TCGGAGTGAACGGATTCGG	192	57.000
GAPDH-R	TGATGACGAGCTTCCCGTTC		
SREBF1-F	CCACCAGCATCAACCACG	133	58.200
SREBF1-R	GCCAAGGAGAAGAGCACCAG		
ACSS2-F	AGATTGGCCCCATTGCCACC	108	59.400
ACSS2-R	GCCTCATGATTTTCCCTGAGCG		
AGPAT6-F	TATGTGAAGGATGTGGATGATGG	140	58.200
AGPAT6-R	TGTCGGAAGAGGTTAGGGAAGT		
FASN-F	AGACGGTGCTCATTCACTCG	106	57.200
FASN-R	TTTTCGGCTGACCCCACAAG		

Table S5 The result of the assembly

Items	Count
Total length /bp	6467923563
Total No. /pc	15361808
Mean Length/bp	421.040
Max Length/bp	23691
Min Length/bp	201
GC Content/%	43.360

Table S6 Alpha diversity analysis

	Group			SEM	*P*-value		
	A1A1	A1A2	A2A2		A1A1VSA1A2	A1A1VSA2A2	A1A2VSA2A2
shannon	5.870	5.650	5.960	0.050	0.310	0.770	0.460
simpson	0.990	0.990	0.990	0.000	0.860	0.750	0.620
ACE	26178.810	24918.050	26956.900	387.570	0.050	0.580	0.140

Table S7 Differences in relative abundance of microflora of dairy cows among the three genotypes

Biomarker	Logarithm value	Groups	LDA_value	*P*-value

g__Pediococcus	2.40	A2A2	2.200	0.005
f__Streptococcaceae	4.15	A2A2	3.990	0.003
f__Streptococcaceae	3.27	A2A2	2.620	0.036
g__Pasteurella	2.91	A2A2	2.600	0.017
f__Pseudomonadaceae	3.10	A2A2	2.750	0.011
f__Acetobacteraceae	4.05	A2A2	3.870	0.002
g__Gluconobacter	2.39	A2A2	2.220	0.002
g__Xanthomonas	4.08	A2A2	3.930	0.001
g__Stenotrophomonas	2.99	A2A2	2.810	0.001
g__Pichia	2.44	A2A2	2.150	0.018
g__Glomus	2.62	A2A2	2.280	0.038
g__Pseudoxanthomonas	2.31	A2A2	2.150	0.001
f__Neisseriaceae	2.81	A2A2	2.390	0.040
g__Streptococcus	3.24	A2A2	2.620	0.036
g__Mannheimia	3.51	A2A2	3.250	0.006
g__Luteimonas	2.44	A2A2	2.280	0.002
f__Xanthomonadaceae	4.15	A2A2	3.990	0.003
f__Anaplasmataceae	4.19	A2A2	3.930	0.003
f__Anaplasmataceae	2.43	A2A2	2.070	0.020
p__Cyanobacteria	3.01	A2A2	2.560	0.022
g__Komagataeibacter	2.43	A2A2	2.260	0.007
f__Pichiaceae	2.87	A2A2	2.560	0.030
g__Acetobacter	4.02	A2A2	3.850	0.004
g__Pseudomonas	3.00	A2A2	2.740	0.010
f__Barnesiellaceae	2.902	A1A2	2.080	0.007
f__Muribaculaceae	3.42	A1A2	2.510	0.001
f__Tannerellaceae	3.60	A1A2	2.630	0.034
g__Aidingimonas	0	A1A2	2.240	0.029
g__Acidaminococcus	3.04	A1A1	2.430	0.019
g__Bibersteinia	3.82	A1A1	3.350	0.008
g__Klebsiella	3.20	A1A1	2.620	0.013
g__Staphylococcus	3.51	A1A1	3.090	0.016
f__Moraxellaceae	3.94	A1A1	3.380	0.013
f__Zhaonellaceae	2.80	A1A1	2.300	0.002
g__Acinetobacter	3.89	A1A1	3.350	0.015
g__Zhaonella	2.81	A1A1	2.300	0.002
g__Puteibacter	3.56	A1A1	3.050	0.011
g__Enterobacter	3.25	A1A1	2.730	0.040
g__Actinobacillus	2.79	A1A1	2.350	0.007
g__Chryseobacterium	2.80	A1A1	2.190	0.024
g__Dialister	3.04	A1A1	2.480	0.027
f__Pasteurellaceae	4.12	A1A1	3.740	0.010
g__Skermania	0	A1A1	2.110	0.006
g__Carideicomes	3.18	A1A1	2.670	0.034

g__Glaesserella	2.72	A1A1	2.270	0.004
p__Proteobacteria	4.92	A1A1	4.510	0.015
f__Staphylococcaceae	3.53	A1A1	3.100	0.019
f__Halomonadaceae	2.89	A1A1	2.250	0.046

Table S8 Significantly different metabolites in A1A1 cows and A1A2 cows

Retention	Ion	Metabolites	VIP	FC	Regulation	*P*-value
5.204	pos	Aflatoxin B1 dialcohol	20.420	0.556	↓	0.024
5.802	pos	(10S,13S,16R)-d14-9-PhytoF[9S,12S]	18.487	0.570	↓	0.010
7.489	pos	Dodecanedioic acid	12.332	0.507	↓	0.012
5.684	neg	3-(4-Hydroxyphenyl)-3,5,6,8-tetrahydro-2H-chromene-	8.140	0.357	↓	0.016
5.219	pos	Cinncassiol A	7.890	0.570	↓	0.007
5.413	pos	2,3-dinor-PGE1	7.805	0.659	↓	0.006
4.445	pos	2,8-Quinolinediol	7.458	0.538	↓	0.016
6.415	neg	Tupichinol A	7.155	0.556	↓	0.046
9.182	pos	2S-hydroxy-3-(10Z-tetradecenoyloxy)-propanoic acid	7.098	0.347	↓	0.019
5.060	pos	Kuhlmanniquinol	6.186	0.566	↓	0.007
5.055	neg	4-Hydroxy-5,7,4'-trimethoxyflavan	5.767	0.578	↓	0.009
6.254	neg	2,3-Dinor-TXB2	5.618	0.549	↓	0.005
5.413	pos	oscr#19	5.409	0.560	↓	0.003
6.794	pos	oscr#24	5.110	0.473	↓	0.004
5.204	pos	Glyzarin	5.004	0.520	↓	0.024
9.166	neg	ascr#18	4.946	0.356	↓	0.020
7.303	pos	(Z)-5-[(2R,3S,4S)-4-Hydroxy-2-[(E)-3-hydroxyoct-1-	4.943	0.659	↓	0.033
5.563	pos	Farnesyl Thiosalicylic Acid	4.815	0.543	↓	0.006
5.204	pos	bhas#20	4.422	0.560	↓	0.010
4.936	neg	Isoimperatorin	4.348	0.421	↓	0.039
12.135	pos	Nuatigenin	4.337	0.022	↓	0.046
5.560	neg	(7Z)-14-hydroxy-10,13-dioxoheptadec-7-enoic acid	4.290	0.555	↓	0.008
6.845	pos	2-Hydroxyundec-9-enoylcarnitine	4.200	0.661	↓	0.013
5.012	pos	Mucroquinone	4.090	0.389	↓	0.028
5.211	neg	Cycloartomunoxanthone	4.006	0.578	↓	0.039
5.598	pos	3'-N'-Acetylfusarochromanone	3.967	0.218	↓	0.033
8.882	neg	Brefeldin A	3.922	0.559	↓	0.042
6.659	pos	Undecanedioic acid	3.918	0.501	↓	0.022
4.798	pos	4,1-Benzoxazepine	3.853	0.555	↓	0.003
5.187	pos	Entecavir	3.852	0.667	↓	0.020
5.249	pos	3-(2-Furanylmethyl)-1H-pyrrole	3.846	0.543	↓	0.014
7.269	neg	Ipomeatetrahydrofuran	3.760	0.654	↓	0.044
4.450	neg	4-Acetamidobenzoic acid	3.688	0.606	↓	0.032
5.785	pos	Mycinonic acid III	3.642	0.626	↓	0.016
9.044	pos	9,10-Epoxyoctadecanoic acid	3.493	0.764	↓	0.040
6.807	neg	ent-16-epi-16-D1t-PhytoP	3.439	0.672	↓	0.042

5.546	pos	Monic acid	3.389	0.631	↓	0.005
5.820	pos	8,8a-Deoxyoleandolide	3.373	0.711	↓	0.019
7.303	pos	6-Hexyltetrahydro-2H-pyran-2-one	3.349	0.562	↓	0.021
6.592	pos	(Z)-5-((2R,3S,4S,6R)-4,6-Dihydroxy-2-((S,E)-3-	3.278	0.572	↓	0.011
6.214	pos	Sorbitan laurate	3.199	0.513	↓	0.006
11.278	pos	(25R)-3beta-hydroxycholest-5-en-7-one-26-oate	3.109	0.171	↓	0.023
9.896	pos	PGF1a alcohol	3.063	0.429	↓	0.033
5.481	pos	1,3,7-Trimethyl-8-nonylpurine-2,6-dione	2.974	0.543	↓	0.002
4.936	neg	(2S,3S)-3,7,4'-trihydroxy-5-methoxy-6-methylflavanone	2.966	0.403	↓	0.039
4.818	neg	7Z-Decenyl acetate	2.965	0.603	↓	0.035
9.182	pos	7-hydroxy-10E,16-heptadecadien-8-ynoic acid	2.800	0.350	↓	0.018
4.031	pos	9-amino-nonanoic acid	2.800	0.517	↓	0.012
5.684	neg	Aflatoxin Ex2B1	2.772	0.517	↓	0.026
4.413	pos	1-(2-methoxy-13-methyl-6Z-tetradecenyl)-sn-glycero-3-	2.744	0.358	↓	0.008
5.075	pos	5,6-Dihydroxyprostaglandin F1a	2.719	0.608	↓	0.003
7.336	pos	PE-Cer(d14:1(4E)/16:0)	2.674	0.103	↓	0.015
5.060	pos	3'-Hydroxy-T2-triol	2.654	0.489	↓	0.004
4.539	neg	5,4'-Dihydroxy-7-methoxy-8-methylflavanone	2.621	0.500	↓	0.022
2.113	pos	5,7,3',4',5'-Pentahydroxyflavanone	2.609	0.558	↓	0.039
4.733	pos	Dactimicin	2.598	0.552	↓	0.001
6.980	pos	7-hydroxygranisetron	2.573	0.399	↓	0.009
11.400	pos	(23S,25R)-1alpha,25-dihydroxyvitamin D3 26,23-lactol /	2.526	0.030	↓	0.049
13.220	neg	15-methyl-15R-PGE2	2.513	0.152	↓	0.013
5.973	pos	DG(2:0/18:3(9,11,15)-OH(13)/0:0)	2.499	0.320	↓	0.013
5.613	neg	20-Hydroxy-PGE2	2.495	0.683	↓	0.009
4.880	pos	11-Hydroxyyangonin	2.494	0.401	↓	0.002
6.201	neg	5C-aglycone	2.486	0.420	↓	0.010
5.204	pos	5-Megastigmen-7-yne-3,9-diol 9-glucoside	2.478	0.496	↓	0.004
4.445	pos	Flazine methyl ether	2.475	0.399	↓	0.011
4.946	pos	Benzoyl-fvr-pna	2.422	0.553	↓	0.040
8.314	pos	14beta-14,15-dihydrocalonysterone	2.414	0.031	↓	0.033
4.913	pos	Inproquone	2.350	0.433	↓	0.024
4.767	pos	(Z)-N-Feruloyl-5-hydroxyanthranilic acid	2.347	0.532	↓	0.024
5.228	neg	Scillaren A	2.332	0.545	↓	0.047
10.740	pos	11-Hydroxyheptadecanoylcarnitine	2.327	0.295	↓	0.019
7.489	pos	PC(3:0/3:0)	2.324	0.475	↓	0.048
4.814	pos	3,4-Dihydroxy-2-methoxy-4-methyl-3-[2-methyl-3-(3-	2.281	0.557	↓	0.010
4.132	pos	5-Amino-2,3-dihydro-6-(3-hydroxy-4-methoxy-1-	2.260	0.489	↓	0.042
6.794	pos	3-Epiaphidicolin	2.234	0.529	↓	0.001
6.219	neg	(9R,12S,15S)-d10-13-PhytoF[13S,16S]	2.210	0.444	↓	0.010
4.936	neg	oscr#14	2.194	0.714	↓	0.025
4.814	pos	4,4'-Thiobis(6-tert-butyl-m-cresol)	2.193	0.697	↓	0.010
7.117	pos	4,14-dihydroxy-octadecanoic acid	2.190	0.574	↓	0.001
6.144	pos	Dihydrocycloartomunin	2.186	0.399	↓	0.018

5.072	neg	Isoflupredone acetate	2.172	0.263	↓	0.047
5.028	pos	Neobyakangelicol	2.170	0.390	↓	0.037
5.092	pos	2alpha-Hydroxyalantolactone	2.167	0.641	↓	0.008
5.028	pos	2-Propanamine, N,N,2-trimethyl-1-((3-phenyl-2-	2.144	0.250	↓	0.042
6.540	neg	Isosativan	2.144	0.513	↓	0.013
0.754	pos	[(2R,3S,4R,5R)-3,4,5,6-Tetrahydroxy-1-oxohexan-2-yl]	2.143	0.619	↓	0.034
5.187	pos	(3beta,9beta)-7-Drimene-3,11,12-triol	2.129	0.545	↓	0.012
6.231	pos	PGF2alpha-dihydroxypropanylamine	2.118	0.409	↓	0.010
4.750	neg	Phenmedipham	2.103	0.558	↓	0.035
4.527	pos	Tetranor-PGD1	2.091	0.304	↓	0.012
5.754	neg	Involucrin	2.089	0.518	↓	0.021
6.450	neg	Lonchocarpenin	2.077	0.373	↓	0.031
5.413	pos	9-epi-9-F1t-PhytoP	2.070	0.685	↓	0.023
4.913	pos	galactosyl hydroxylysine	2.059	0.621	↓	0.005
7.534	neg	Gibberellin A53	2.057	0.781	↓	0.005
7.569	neg	15-Keto-prostaglandin E2	2.043	0.826	↓	0.047
8.143	pos	11,12-dihydroxy arachidic acid	2.037	0.742	↓	0.049
5.613	neg	3,4-DHPEA-EA	2.026	0.659	↓	0.021
6.344	neg	8-Hydroxy-4,8-dimethyl-4E,9-decadienoic acid	2.020	0.662	↓	0.048
6.367	pos	hexadecanedioic acid mono-L-carnitine ester	1.996	0.416	↓	0.013
6.964	neg	Lactapiperanol C	1.984	0.450	↓	0.007
7.091	neg	2-((3,5-Dihydroxyphenyl)amino)acetic acid	1.970	0.402	↓	0.043
5.187	pos	Riesling acetal	1.969	0.683	↓	0.012
7.660	pos	PE-Cer(d15:2(4E,6E)/18:0(2OH))	1.968	0.130	↓	0.025
7.481	neg	3-[4-[(E)-2-[6-(Dibutylamino)naphthalen-2-	1.964	0.556	↓	0.016
6.454	pos	Tarazepide	1.963	0.335	↓	0.026
8.687	neg	oscr#17	1.954	0.747	↓	0.032
5.126	neg	3,5-Diferuloylquinic acid	1.953	0.321	↓	0.043
9.744	pos	Taccagenin	1.949	0.045	↓	0.049
5.195	neg	Lactarofulvene	1.949	0.520	↓	0.022
5.560	neg	4'-Hydroxy-2'-methoxychalcone	1.942	0.255	↓	0.044
6.129	neg	Puerarol	1.933	0.399	↓	0.030
5.596	neg	Methyl clofenapate	1.912	0.468	↓	0.013
4.493	pos	Guaiaretic acid	1.892	0.546	↓	0.031
4.542	pos	5,2'-Dihydroxy-7,4',5'-trimethoxyflavanone	1.891	0.594	↓	0.035
4.880	pos	4-(4-Methylcyclohexyl)-4-oxobutanoic acid	1.890	0.569	↓	0.013
5.055	neg	2H-1-Benzopyran-2-one, 7-[[2-(acetylamino)-2-deoxy-	1.885	0.551	↓	0.028
6.254	neg	17-F2t-dihomo-IsoP	1.871	0.284	↓	0.006
1.446	neg	2-(2-Oxopropanoyloxy)propanoic acid	1.864	0.553	↓	0.048
7.481	neg	PA(18:4(6Z,9Z,12Z,15Z)/18:4(6Z,9Z,12Z,15Z))	1.850	0.507	↓	0.041
5.397	pos	Reproterol	1.844	0.410	↓	0.002
9.896	pos	Aminopentol	1.844	0.354	↓	0.016
7.481	neg	N'-Hydroxysaxitoxin	1.840	0.600	↓	0.025
7.838	neg	1-(9Z-tetradecenoyl)-glycero-3-phosphate	1.837	0.406	↓	0.012

5.546	pos	gibberellin A36	1.833	0.519	↓	0.005
5.598	pos	Formylfusarochromanone	1.830	0.239	↓	0.029
5.878	neg	5-Hydroxydecanedioylcarnitine	1.819	0.478	↓	0.016
6.076	pos	Obacunone 17-O-beta-D-glucoside	1.794	0.032	↓	0.036
5.243	neg	1-[Ethyl-(6-hydrazinylpyridazin-3-yl)amino]propan-2-ol	1.771	0.698	↓	0.006
9.890	neg	4,6-Nonadecanedione	1.758	0.292	↓	0.032
7.481	neg	3-Hydroxylidocaine	1.757	0.505	↓	0.014
8.627	pos	MG(0:0/a-17:0/0:0)[rac]	1.750	0.512	↓	0.006
4.282	pos	Crocin 3	1.726	0.584	↓	0.031
7.015	pos	(25R)-12beta-acetyl-17alpha-hydroxyspirost-4-en-3-one	1.721	0.402	↓	0.047
5.091	neg	Razaxaban	1.720	0.411	↓	0.041
5.331	pos	Myricanene B 5-[arabinosyl-(1->6)-glucoside]	1.719	0.355	↓	0.008
9.627	pos	N-Linoleoyl Lysine	1.717	0.257	↓	0.015
4.768	neg	3,10-dihydroxydecanoic acid	1.712	0.585	↓	0.042
5.752	pos	4,11,13,15-Tetrahydroridentin B	1.710	0.703	↓	0.032
6.997	pos	DG(i-12:0/20:5(7Z,9Z,11E,13E,17Z)-3OH(5,6,15)/0:0)	1.706	0.075	↓	0.011
5.701	pos	Coriolic acid	1.698	0.539	↓	0.037
11.278	pos	iso (4E,15-methyl-d16:1) sphingosine	1.691	0.232	↓	0.021
6.760	pos	Dinor-PGD2	1.690	0.555	↓	0.008
7.000	neg	Prostaglandin I3	1.681	0.644	↓	0.019
4.381	neg	Dihydromyricetin 3-rhamnoside	1.678	0.287	↓	0.022
8.023	pos	13-Labdene-2,8,15-triol (ent-2a,8a,13E)-form)	1.669	0.416	↓	0.028
0.783	pos	Cndac	1.639	0.487	↓	0.034
12.073	neg	2-hydroxy-3-methylhexadecanoic acid	1.633	0.417	↓	0.044
9.062	pos	9,10,13-TriHOME	1.612	0.701	↓	0.006
8.020	neg	1-(3-Furanyl)-6,7-dihydroxy-4,8-dimethyl-1-nonanone	1.610	0.627	↓	0.020
8.291	neg	25-Acetylvulgaroside	1.609	0.020	↓	0.036
4.798	pos	Trimethoprim	1.602	0.480	↓	0.024
5.313	neg	Sumarotene	1.598	0.690	↓	0.005
5.808	neg	Glas 20	1.588	0.668	↓	0.019
5.785	pos	2,2'-(3-methylcyclohexane-1,1-diyl)diacetic acid	1.573	0.756	↓	0.010
5.204	pos	2,3-Dimethoxy-5-methyl-6-(9'-carboxynonyl)-1,4-	1.565	0.590	↓	0.005
5.878	neg	12-hydroxy-3Z,6Z-dodecadienoic acid	1.565	0.519	↓	0.010
4.867	neg	Citreovirenone	1.563	0.382	↓	0.005
5.109	pos	(4Z,7Z)-Hexadeca-4,7-dienedioylcarnitine	1.563	0.257	↓	0.039
7.305	neg	Kurilensoside G	1.562	0.492	↓	0.031
11.104	pos	9-(3-Methyl-5-propylfuran-2-yl)nonanoylcarnitine	1.551	0.581	↓	0.002
7.091	neg	Isoeugenol benzyl ether	1.551	0.393	↓	0.043
4.493	pos	Isanolic acid	1.549	0.413	↓	0.015
6.201	neg	Berkeleylactone E	1.544	0.451	↓	0.001
8.882	neg	1-[(1R,2R,3S,4S)-3-Hydroxy-4,7,7-trimethyl-2-	1.529	0.669	↓	0.048
6.823	neg	2-hydroxy-nonadecanoic acid	1.525	0.521	↓	0.017
7.785	neg	11-methoxy-octadecanoic acid	1.516	0.503	↓	0.023
4.460	pos	4,5-Dihydrovomifoliol	1.514	0.697	↓	0.033

7.489	pos	4E,6E-undecenal	1.511	0.574	↓	0.015
8.006	pos	3alpha,7alpha,12alpha,16alpha-tetrahydroxy-5beta-cholan-	1.510	0.571	↓	0.008
4.366	pos	Berkeleylactone !	1.507	0.602	↓	0.017
7.659	neg	5-(Biotinamido)pentylamine	1.505	0.278	↓	0.031
5.177	neg	(-)-Euphomine	1.503	0.424	↓	0.017
5.195	neg	3-(6-((4-(Trifluoromethoxy)phenyl)amino)pyrimidin-4-	1.499	0.577	↓	0.036
10.377	pos	14-Methylpentadecanoylcarnitine	1.497	0.250	↓	0.024
4.834	neg	vanillyl mandelic acid	1.494	0.412	↓	0.005
7.506	pos	Gibberellin A17	1.492	0.201	↓	0.039
6.665	neg	cis-p-Menthane-1,7,8-triol	1.491	0.482	↓	0.008
4.382	pos	Orientin	1.490	0.165	↓	0.049
5.598	pos	4-[(2R,5R)-5-(6-Aminopurin-9-yl)-3,4-dihydroxyoxolan-	1.483	0.591	↓	0.011
5.632	pos	19-Hydroxyprostaglandin E2	1.479	0.506	↓	0.004
6.896	pos	Ethylene brassylate	1.476	0.637	↓	0.010
4.116	pos	ibho#16	1.465	0.474	↓	0.027
5.055	neg	mezerein	1.465	0.449	↓	0.007
6.219	neg	6-exo-Hydroxyfenchone	1.455	0.549	↓	0.018
6.110	pos	N-(3-hydroxy-decanoyl)-homoserine lactone	1.451	0.501	↓	0.008
10.070	pos	14-Deoxy-20-hydroxyecdysone	1.451	0.269	↓	0.029
4.557	neg	Darexaban glucuronide	1.445	0.576	↓	0.032
5.481	pos	19-Hydroxy-PGE2	1.443	0.488	↓	0.001
7.489	pos	Gosogliptin	1.436	0.383	↓	0.002
5.280	pos	Deca-4,8-dienedioylcarnitine	1.433	0.371	↓	0.018
7.694	pos	3,7-Dihydroxydecanoylcarnitine	1.433	0.630	↓	0.018
6.858	neg	PI(20:1(11Z)/0:0)	1.432	0.560	↓	0.043
4.332	pos	Phenylbutyrylglutamine	1.429	0.525	↓	0.010
6.056	neg	Cfp-aaf-pab	1.423	0.074	↓	0.037
5.228	neg	9,10-Dihydro-2,3,5,7-Phenanthrenetetrol	1.420	0.368	↓	0.033
5.578	neg	Atipamezole	1.406	0.196	↓	0.041
5.384	neg	Cotinine glucuronide	1.406	0.552	↓	0.004
9.628	neg	DG(20:4(6E,8Z,11Z,13E)-2OH(5S,15S)/i-12:0/0:0)	1.406	0.455	↓	0.045
7.018	neg	oscr#3	1.406	0.591	↓	0.008
5.719	neg	3'-Amino-3'-deoxythimidine	1.402	0.611	↓	0.002
5.187	pos	Bisacurone epoxide	1.398	0.646	↓	0.034
4.733	pos	7,2'-Dihydroxy-5,8-dimethyl-4',5'-methylenedioxyflavan	1.391	0.591	↓	0.008
4.936	neg	1-Galactopyranosyl-5-fluorouracil	1.389	0.566	↓	0.035
4.847	pos	Myricanol 5-[arabinosyl-(1->6)-glucoside]	1.388	0.382	↓	0.025
4.557	neg	2-Nonene-1,4-diol	1.382	0.524	↓	0.040
13.690	pos	Cholestane-3b,5a,6b,(25R)26-tetrol	1.376	0.499	↓	<0.001
4.818	neg	trans-p-Menthane-7,8-diol 7-glucoside	1.367	0.667	↓	0.024
6.110	pos	Tetranor-PGF1alpha	1.354	0.641	↓	0.005
5.172	pos	Dapiprazole	1.353	0.467	↓	0.010
7.557	pos	Pteroside Z	1.347	0.680	↓	0.031
10.175	pos	15-hydroxyicosanoic acid	1.334	0.251	↓	0.029

4.637	pos	Estetrol	1.332	0.487	↓	0.015
5.401	neg	7,3'-Dihydroxy-5,4'-dimethoxy-6-formyl-4-	1.331	0.527	↓	0.049
6.201	neg	Glutamic acid-betaxanthin	1.322	0.457	↓	0.013
6.334	pos	Dibenzo-P-dioxin	1.322	0.271	↓	0.017
7.851	pos	PE-Cer(d14:2(4E,6E)/20:0)	1.314	0.048	↓	0.010
7.336	pos	Nummularine A	1.308	0.107	↓	0.014
4.902	neg	Cybutryne	1.306	0.514	↓	0.013
9.997	neg	Saringosterol 3-glucoside	1.299	0.354	↓	0.032
4.698	neg	Arphamenine B	1.299	0.540	↓	0.008
8.784	pos	ubiquinone-8	1.293	0.704	↓	0.041
10.035	pos	2-methyl-7R,8S-Epoxy-17-octadecene	1.292	0.300	↓	0.035
5.684	neg	Pentaleno(1,6a-c)pyran-5-carboxylic acid, 1-	1.288	0.381	↓	0.015
4.953	neg	Cynaroside A	1.286	0.587	↓	0.005
5.028	pos	5-Hydroxydec-8-enoylcarnitine	1.282	0.491	↓	0.031
5.401	neg	Triamcinolone	1.278	0.473	↓	0.006
10.241	pos	Nordihydrocapsiate	1.278	0.696	↓	0.020
8.400	pos	Monomenthyl succinate	1.277	0.669	↓	0.042
4.867	neg	Rosmarinic acid	1.271	0.563	↓	0.025
5.419	neg	(S)-p-Menth-1-ene-4,7-diol 4-glucoside	1.264	0.617	↓	0.033
5.397	pos	3,14-Dihydroxy-11,13-dihydrocostunolide	1.261	0.643	↓	0.010
3.931	neg	Pyrocatechol	1.256	0.434	↓	0.048
5.464	pos	1,17-Diamino-4,9,13-triazaheptadecane	1.254	0.476	↓	0.022
5.249	pos	3-(4-Methyl-3-pentenyl)thiophene	1.254	0.529	↓	0.012
5.785	pos	5-Hydroxyenterolactone	1.248	0.506	↓	0.007
14.313	pos	TG(8:0/8:0/15:0)	1.245	0.305	↓	0.006
6.020	neg	bhos#16	1.240	0.635	↓	0.012
10.393	pos	Diosgenin	1.238	0.085	↓	0.031
5.524	neg	Daumone	1.237	0.625	↓	0.039
4.953	neg	indole-3-acetyl-glutamine	1.235	0.616	↓	0.027
4.382	pos	DIACETYLDIDEISOVALERYL-RHODOMYRTOXIN	1.230	0.427	↓	0.038
4.382	pos	Citrusinine I	1.230	0.514	↓	0.017
10.879	pos	(20R,24R)-20-fluoro-1alpha,24-dihydroxy-26,27-	1.229	0.390	↓	0.022
4.801	neg	(S)-3-Methylthiohexyl butyrate	1.228	0.588	↓	0.001
5.234	pos	DN-isobutylamide	1.227	0.344	↓	0.011
5.948	neg	LYSYL-TYROSYL-LYSINE	1.227	0.617	↓	0.049
6.111	neg	oscr#16	1.224	0.704	↓	0.022
5.785	pos	Pumilaisoflavone C	1.212	0.391	↓	0.012
7.767	neg	(+)-12-hydroxy-9Z-hexadecenoic acid	1.212	0.611	↓	0.013
6.629	neg	Milbemycin D	1.208	0.472	↓	0.040
6.790	neg	N-(2,3,4-Trihydroxybutyl)-L-valine	1.208	0.552	↓	0.002
5.092	pos	ent-16-epi-13-E4c-NeuroP	1.204	0.318	↓	0.010
5.413	pos	8-Hydroxygalangin 7-methyl ether 8-acetate	1.203	0.467	↓	0.029
10.275	pos	Macamide B	1.196	0.264	↓	0.042
8.505	pos	FAHFA(12:1(5Z)/3-O-8:0)	1.195	0.525	↓	0.016

9.510	pos	N-oleoyl threonine	1.190	0.250	↓	0.011
5.754	neg	3-methylflavone-8-carboxylic acid	1.187	0.526	↓	0.017
3.425	pos	Glycerol 1,2-dimethacrylate	1.186	0.520	↓	0.015
6.056	neg	2-Methylbenzoic acid	1.184	0.370	↓	0.038
6.237	neg	2,3-Dinor-TXB1	1.184	0.373	↓	0.006
5.187	pos	Gibberellin A23	1.184	0.480	↓	0.003
4.864	pos	Phaseic acid	1.183	0.715	↓	0.034
5.560	neg	Glycerol 1-(5-hydroxydodecanoate)	1.181	0.555	↓	0.012
4.919	neg	prednisolone-16alpha-carboxylic acid	1.181	0.487	↓	0.021
4.460	pos	Siguazodan	1.179	0.623	↓	0.034
9.182	pos	N-(2(S)-(Acetylthiomethyl)-3-(2-methylphenyl)-1-	1.179	0.276	↓	0.007
4.936	neg	2-Phenyl-3,4-dihydrochromen-2-ol	1.179	0.439	↓	0.037
6.344	neg	Altamisic acid; 3-[(3aS,8R,8aR)-8-Hydroxy-6,8-dimethyl-	1.178	0.680	↓	0.036
9.643	pos	9-Methyltetradecanoylcarnitine	1.177	0.429	↓	0.004
4.573	pos	Phe-Pro-Ile	1.175	0.546	↓	0.015
6.450	neg	13-Dihydrodaunorubicin	1.175	0.226	↓	0.029
6.402	pos	[(8S,9S,13S,14S,17R)-3,17-Dihydroxy-13-methyl-	1.173	0.509	↓	0.021
5.296	pos	1-Tert-butyl 4-ethyl 5-oxoazepane-1,4-dicarboxylate	1.171	0.420	↓	0.018
10.106	pos	Aragusterol B	1.168	0.525	↓	0.047
4.970	neg	1-(2-Methylbenzo[d]oxazol-6-yl)-3-(1,5-naphthyridin-4-	1.164	0.465	↓	0.028
3.306	pos	(2E,4E)-Hexa-2,4-dienedioylcarnitine	1.158	0.548	↓	0.012
4.847	pos	O-11	1.158	0.375	↓	0.034
4.867	neg	N-Palmitoyl Cysteine	1.156	0.385	↓	0.022
8.314	pos	PE-Cer(d16:2(4E,6E)/18:0(2OH))	1.152	0.048	↓	0.007
8.882	neg	3-(2-(Diisopropylamino)ethyl)-1H-indol-5-OL	1.150	0.487	↓	0.036
6.646	neg	Nor-cocaine	1.144	0.613	↓	0.017
6.254	neg	Cucurbitacin P	1.138	0.406	↓	0.015
5.195	neg	CP-471474	1.137	0.634	↓	0.026
6.646	neg	Oxypinnatanine	1.136	0.599	↓	0.026
6.237	neg	gamma-Glutamylfelinylglycine	1.135	0.553	↓	0.039
5.382	pos	Dihydroxyeicosatrienoic acid	1.134	0.675	↓	0.022
4.493	pos	Semilepidinoside A	1.131	0.562	↓	0.011
8.887	pos	8,9-Dihydro-5-hydroxy-8-(1-hydroxy-1-methylethyl)-6-(1-	1.127	0.504	↓	0.014
8.974	pos	3-(1-Propyl-3-piperidinyl)phenol	1.126	0.440	↓	0.017
7.557	pos	3,7-Dihydroxy-12-oxocholanoic acid	1.125	0.574	↓	0.012
9.450	neg	9,13-dihydroxy-12-ethoxy-10-octadecenoic acid	1.124	0.602	↓	0.026
7.091	neg	Dopaxanthin quinone	1.124	0.413	↓	0.049
6.692	pos	Sofalcone	1.122	0.419	↓	0.016
4.988	neg	4-((6-Methoxyquinolin-8-yl)amino)pentanoic acid	1.122	0.423	↓	0.014
7.920	pos	Diethylpropion	1.121	0.487	↓	0.022
8.125	pos	Chenodeoxycholylaspartic acid	1.120	0.460	↓	0.037
10.070	pos	Butaprost	1.118	0.562	↓	0.028
5.887	pos	2-Methoxy-4-(2-(methylamino)propyl)phenyl beta-D-	1.114	0.577	↓	0.001
6.807	neg	Botrydial	1.111	0.395	↓	0.010

5.878	neg	Chenodeoxycholic acid 3-glucuronide	1.109	0.462	↓	0.033
5.249	pos	Efaproxiral	1.107	0.571	↓	0.038
11.295	pos	N-(15-methyl-3-hydroxy-hexadecanoyl)-glycine	1.106	0.565	↓	0.034
4.476	pos	Agestricin D	1.103	0.475	↓	0.010
3.356	pos	3,5,7-Trimethylepicatechin	1.102	0.513	↓	0.043
5.887	pos	[8]-Gingerdiol	1.099	0.304	↓	0.020
4.604	pos	2,8-Dihydroxyquinoline-beta-D-glucuronide	1.099	0.610	↓	0.004
5.649	pos	(4r,5s,6s,7r)-4,7-Dibenzyl-5,6-dihydroxy-1,3-bis[4-	1.095	0.432	↓	0.047
5.768	pos	Mitoflaxone	1.095	0.479	↓	0.011
4.249	pos	(5E)-3-Hydroxyhept-5-enoylcarnitine	1.092	0.334	↓	0.027
4.382	pos	Isradipine	1.090	0.650	↓	0.015
7.481	neg	2-Pyrrol-1-yl-1H-imidazole	1.089	0.506	↓	0.020
11.954	neg	Bendigole D	1.089	0.138	↓	0.016
7.506	pos	MeOSuc-Ala-Ala-Pro-Val-PNA	1.088	0.268	↓	0.026
7.473	pos	Labetalol	1.087	0.346	↓	0.030
6.559	pos	2-Hydroxy-4-octenoylcarnitine	1.086	0.296	↓	0.003
4.165	neg	Benzyloxycarbonyl-Val-Ala-Asp-fluoromethylketone	1.078	0.507	↓	0.020
5.560	neg	Euchrenone b4	1.076	0.282	↓	0.040
1.720	pos	4(1H)-Pteridinone, 2-amino-6-methyl-	1.074	0.303	↓	0.001
6.272	neg	bhos#24	1.073	0.455	↓	0.008
9.060	neg	ent-3alpha,14alpha,16beta,17-tetrahydroxyatisane	1.072	0.684	↓	0.044
6.231	pos	Umbelliprenin	1.072	0.391	↓	0.003
6.896	pos	Tetranor-PGE1	1.072	0.632	↓	0.019
4.233	pos	2-[[10-(2-Hydroxyethoxy)anthracen-9-yl]methylamino]-2-	1.069	0.558	↓	0.001
6.823	neg	(Z)-7-[(1R,3R)-3-Hydroxy-2-[(1E,3S,5Z)-3-hydroxyocta-	1.066	0.592	↓	0.035
6.290	neg	Senkyunolide N	1.066	0.730	↓	0.027
5.984	neg	3,8-Dihydroxy-6-methoxy-7(11)-eremophilen-12,8-olide	1.065	0.568	↓	0.003
7.237	pos	Delcorine	1.064	0.326	↓	0.043
6.165	neg	(E)-2-Octen-1-ol	1.063	0.785	↓	0.034
6.201	neg	Annuolide C	1.062	0.411	↓	0.012
9.896	pos	Hexadecenoylcarnitine	1.059	0.170	↓	0.021
5.684	neg	SPARSOMYCIN	1.048	0.421	↓	0.022
9.785	neg	10-Deoxymethynolide	1.045	0.586	↓	0.017
5.596	neg	PI(19:0/0:0)	1.045	0.346	↓	0.029
6.272	neg	L-Proline, 1-(2-methyl-3-(methylthio)-1-oxopropyl)-, (S)-	1.043	0.465	↓	0.003
4.493	pos	Pteroside B	1.042	0.607	↓	0.017
8.255	neg	Dacarbazine	1.038	0.267	↓	0.009
5.277	neg	20-Hydroxy-PGF2a	1.037	0.745	↓	0.005
12.536	neg	Lepidine C	1.032	0.375	↓	0.033
10.175	pos	24-Oxo-1alpha,25-dihydroxyvitamin D3	1.032	0.279	↓	0.042
5.826	neg	6-Hydroxyhexadecanedioylcarnitine	1.031	0.630	↓	0.029
4.919	neg	3',4'-Methylenedioxy epicatechin 5,7-dimethyl ether	1.027	0.615	↓	0.020
4.485	neg	7-Hydroxy-5-methoxy-4-methyl-3-(4-methylpiperazin-1-	1.025	0.600	↓	0.030
7.179	neg	S-(2-(N,N-Diisopropylamino)ethyl)isothiourea	1.021	0.627	↓	0.001

4.349	pos	10-hydroxy-11-dodecenoic acid	1.018	0.628	↓	0.037
7.456	pos	Bucindolol	1.016	0.477	↓	0.021
4.266	pos	Erythro-9-hydroxy-nonyl-adenine	1.014	0.528	↓	0.005
4.129	neg	Diphenyl(2,4,6-trimethylbenzoyl)phosphine oxide	1.014	0.608	↓	0.029
5.684	neg	PHODiA-PA	1.012	0.265	↓	0.022
3.804	neg	Amorphaquinone	1.012	0.397	↓	0.006
6.248	pos	Tetraphenylethylene	1.011	0.475	↓	0.001
4.732	neg	(1S,2S,4S,5S)-2,4,7-Thujanetriol 4-glucoside	1.009	0.600	↓	0.009
4.953	neg	(-)-11-hydroxy-9,10-dihydrojasmonic acid 11-beta-D-	1.009	0.441	↓	0.010
9.200	pos	alpha-N-(3-hydroxy-14-methyl-pentadecanoyl)-ornithine	1.001	0.275	↓	0.005
3.695	pos	4-Chloro-L-phenylalanine	17.562	1.640	↑	0.034
9.322	pos	Pelargonidin 3-sophoroside	17.180	5.720	↑	0.047
11.209	pos	Dibutyl phthalate	16.728	1.779	↑	0.013
8.211	pos	C16 Sphinganine	13.939	1.742	↑	0.028
14.018	pos	pyridine-3,4-diol	11.238	1.895	↑	0.018
6.042	pos	3-Deazaneplanocin A	10.720	27.955	↑	0.001
10.241	pos	(10E,12Z)-(9S)-9-Hydroperoxyoctadeca-10,12-dienoic	10.349	1.998	↑	0.029
3.695	pos	Thiabendazole	10.112	1.646	↑	0.033
10.664	neg	alpha-Dimorphecolic acid	9.459	1.888	↑	0.045
14.018	pos	(S)-Methyl 2,5-diamino-5-oxopentanoate	8.429	1.823	↑	0.021
10.275	pos	9(S)-HOTrE	8.191	2.165	↑	0.043
10.106	pos	N,N-dimethyl-Safingol	7.929	1.741	↑	0.017
12.435	pos	MG(16:0/0:0/0:0)	7.837	2.596	↑	0.024
4.559	pos	N1,N10-Dicoumaroylspermidine	7.722	4.855	↑	0.006
10.949	pos	Ricinoleic acid	7.262	1.742	↑	0.010
0.635	neg	beta-D-fructose 2,6-bisphosphate	6.651	2.335	↑	0.014
12.652	neg	Ptilosteroid B	6.596	1.495	↑	0.033
10.687	pos	Punicic acid	6.487	2.372	↑	0.031
5.075	pos	3'-AZIDO-2',3'-DIDEOXYURIDINE	6.365	25.719	↑	0.010
8.784	pos	3alpha-Hydroxy-5beta-chola-7,9(11)-dien-24-oic Acid	6.294	3.508	↑	0.002
8.759	neg	1alpha-hydroxy-24-(dimethylphosphoryl)-25,26,27-	6.255	1.637	↑	0.030
11.071	pos	1-(11Z-docosenoyl)-glycero-3-phosphate	6.089	1.491	↑	0.033
8.759	neg	Cholic acid	5.964	3.062	↑	0.010
9.291	neg	Pelargonidin 3,5-di-(6-acetylglucoside)	5.953	5.141	↑	0.050
12.364	pos	8,9-Epoxyeicosatrienoic acid	5.761	5.652	↑	0.018
10.652	pos	19-methyl-heneicosanoic acid	5.296	2.123	↑	0.027
11.209	pos	Phthalic acid	4.860	1.680	↑	0.020
0.754	pos	Adenine	4.740	1.621	↑	0.047
13.229	pos	Pyridazine-3,6-diol	4.598	2.000	↑	0.010
12.748	pos	bhas#32	4.567	1.878	↑	0.015
0.723	pos	Proline betaine	4.521	1.676	↑	0.009
9.744	pos	Armillaripin	4.513	2.025	↑	0.006
8.539	pos	Soyasaponin III	4.401	5.279	↑	0.004
10.292	pos	(+)-15,16-Dihydroxyoctadecanoic acid	4.387	1.471	↑	0.043

2.924	pos	(1S,4R)-1-Hydroxy-2-oxolimonene	4.342	1.916	↑	0.012
6.385	pos	Medicagenic acid 3-O-b-D-glucuronide 28-O-[b-D-	4.101	4.326	↑	0.017
3.795	pos	5S,6S-epoxy-15R-hydroxy-ETE	4.044	1.795	↑	0.034
8.759	neg	All trans decaprenyl diphosphate	3.995	1.585	↑	0.029
9.838	neg	(3R,4S,5S,6S)-6-[4-Chloro-2-(furan-2-ylmethylamino)-5-	3.964	1.455	↑	0.042
0.628	pos	Monoethylhexyl phthalic acid	3.922	2.147	↑	0.023
13.229	pos	Polyglycerol esters of fatty acids	3.736	1.371	↑	0.036
7.134	pos	N-(9-Oxodecyl)acetamide	3.713	1.947	↑	0.026
10.664	neg	15-Deoxyprostaglandin J2	3.678	1.676	↑	0.034
8.759	neg	Hordatine A	3.663	1.726	↑	0.025
0.693	pos	Creatinine	3.630	4.417	↑	0.008
10.226	pos	3b-Hydroxy-5-cholenoic acid	3.585	2.274	↑	0.001
8.002	neg	1-[[Difluoro-(2,3,4-trifluorophenyl)methoxy]-	3.566	1.460	↑	0.036
9.847	pos	3-Oxotridecanoylcarnitine	3.548	3.693	↑	0.030
4.637	pos	Apigenin 7-[feruloyl-(->2)-glucuronyl-(1->2)-glucuronide]	3.452	10.599	↑	0.040
0.754	pos	Tranexamic Acid	3.371	1.776	↑	0.008
13.536	pos	MG(18:0/0:0/0:0)	3.256	2.775	↑	0.010
5.075	pos	oscr#9	3.254	23.688	↑	0.008
5.187	pos	(4-Aminophenyl)phosphonic acid	3.239	1.691	↑	0.030
5.044	pos	Tricin 7-glucuronide	3.222	56.529	↑	0.032
14.018	pos	trans-2-Phenylcyclopropylamine	3.215	1.690	↑	0.028
6.437	pos	Quillaic acid 3-[xylosyl-(1->3)-[galactosyl-(1->2)]-	3.181	3.688	↑	0.027
9.661	pos	12-Ketodeoxycholic acid	3.159	1.898	↑	<0.001
11.332	neg	2-Nitrophenyl octyl ether	3.092	1.610	↑	0.024
10.793	pos	Naphtho(1,2-c)furan-3(1H)-one, 4,5,5a,6,7,8,9,9a-	3.052	1.649	↑	0.022
11.192	pos	Tributyl citrate	3.011	1.502	↑	0.031
4.970	neg	Genistein 7-O-glucuronide	2.988	10.211	↑	0.015
4.818	neg	Lercanidipine	2.941	5.483	↑	0.044
9.406	pos	Momordicoside L	2.864	3.766	↑	0.001
10.361	pos	LysoPC(0:0/18:2(9Z,12Z))	2.806	3.246	↑	0.031
4.573	pos	Tricin 7-diglucuronoside	2.781	9.540	↑	0.009
10.949	pos	2-(5,8-Tetradecadienyl)cyclobutanone	2.712	1.741	↑	0.017
9.305	pos	16-Hydroxy-10-oxohexadecanoic acid	2.648	1.840	↑	0.050
14.259	pos	1-Methylhistidine	2.631	1.954	↑	0.015
5.430	pos	5-Methoxyindoleacetate	2.631	9.142	↑	0.006
5.161	neg	Camellioside A	2.628	10.752	↑	0.001
10.309	pos	LysoPE(0:0/18:2(9Z,12Z))	2.605	2.440	↑	0.009
10.343	pos	Arachidoyl Ethanolamide	2.590	1.689	↑	0.028
5.580	pos	Quinaprilat	2.571	5.415	↑	0.014
1.036	pos	(9R,13R)-1a,1b-dihomo-jasmonic acid	2.542	1.606	↑	0.036
5.234	pos	Kudzusaponin SA4	2.515	8.854	↑	0.002
5.039	neg	6-Methoxyluteolin 7-glucuronide methyl ester	2.514	23.216	↑	0.034
4.574	neg	Acacetin 7-glucuronosyl-(1->2)-glucuronide	2.460	9.079	↑	0.008
10.498	pos	14-hydroxy-5Z-tetradecenoic acid	2.439	3.918	↑	0.005

5.172	pos	Camellidin II	2.435	16.470	↑	0.010
4.669	pos	Apigenin 7-glucuronide-4'-(2'''-E-p-	2.432	11.883	↑	0.042
9.873	neg	UDP-alpha-D-xylose	2.423	1.454	↑	0.047
9.908	neg	Ethyl gallate 3-sulfate	2.416	1.471	↑	0.044
11.226	pos	Monoisobutyl phthalic acid	2.415	1.662	↑	0.022
1.460	pos	Homomethionine	2.396	3.254	↑	0.015
8.759	neg	N-Docosahexaenoyl phenylalanine	2.388	1.578	↑	0.028
8.775	neg	15-keto Latanoprost	2.384	2.905	↑	0.011
9.680	neg	N-methyl-4,6,7-trihydroxy-1,2,3,4-tetrahydroisoquinoline	2.332	1.564	↑	0.045
8.057	pos	6alpha-Carissanol	2.328	1.572	↑	0.015
1.067	pos	L-Methionine	2.320	2.350	↑	0.003
13.194	pos	icos#9	2.311	1.544	↑	0.021
7.287	pos	Indole-3-methyl acetate	2.309	16.130	↑	0.003
11.769	pos	5S-HETE di-endoperoxide	2.299	1.794	↑	0.015
5.413	pos	Kudzusaponin SA2	2.274	5.943	↑	0.004
10.241	pos	9,10,11-trihydroxy-12Z-octadecenoic acid	2.268	1.486	↑	0.012
0.677	pos	Disodium phosphate	2.267	4.032	↑	0.020
4.557	neg	Luteolin 3'-methyl ether 7-glucuronosyl-(1->2)-	2.264	9.542	↑	0.006
13.417	pos	L-2,4-diaminobutyric acid	2.246	1.814	↑	0.022
7.109	neg	Calcium ascorbate	2.245	1.385	↑	0.046
10.652	pos	24-Hydroxytetracosanoic acid	2.243	1.614	↑	0.019
5.005	neg	Irtemazole	2.204	2.258	↑	0.014
10.488	neg	13-keto myristic acid	2.192	2.401	↑	0.004
8.759	neg	LysoPC(18:4(6Z,9Z,12Z,15Z)/0:0)	2.183	1.670	↑	0.020
5.560	neg	O-Ethyl S,S-diphenyl phosphorodithioate	2.173	1.419	↑	0.047
5.615	pos	Apoanagallosaponin IV	2.151	15.205	↑	0.021
8.768	pos	PS(22:1(13Z)/TXB2)	2.129	1.806	↑	0.028
11.209	pos	8-Hydroxymianserin	2.125	1.764	↑	0.013
4.818	neg	urea citrate	2.113	1.539	↑	0.039
10.226	pos	Chenodeoxycholic acid	2.091	2.195	↑	0.001
8.922	pos	Pisumsaponin II	2.071	7.553	↑	0.029
10.788	neg	16-Hydroxyhexadecanoic acid	2.063	1.400	↑	0.038
10.983	pos	abietadiene-diol	2.049	1.870	↑	0.016
4.460	pos	Apigenin 7-[glucuronyl-(1->2)-glucuronide] 4'-	2.048	18.484	↑	0.004
10.275	pos	Urocortisone	2.048	3.467	↑	0.003
11.961	pos	1-O-(2R-hydroxy-pentadecyl)-sn-glycerol	2.039	2.019	↑	0.009
9.948	pos	Ambrettolic acid	2.032	1.700	↑	0.014
8.759	neg	DGDG(16:0/18:3(9Z,12Z,15Z))	2.031	1.734	↑	0.027
4.801	neg	Beta-D-Fructose 6-phosphate	2.025	1.507	↑	0.022
11.471	pos	Triethylene glycol bis(3-tert-butyl-4-hydroxy-5-	2.023	3.915	↑	0.044
4.282	pos	Octaethylene glycol	2.021	1.792	↑	0.045
6.420	pos	Medicagenic acid beta-maltoside	2.013	6.939	↑	0.003
7.851	pos	7-Ketodeoxycholic acid	1.995	2.976	↑	0.008
8.453	pos	Gallicynoic acid A	1.994	1.558	↑	0.032

10.088	pos	Clausarinol	1.983	2.062	↑	0.005
7.971	pos	3beta,12alpha-Dihydroxy-5beta-cholestan-26-oic acid	1.975	3.684	↑	0.009
9.965	pos	5,8-tetradecadienal	1.972	1.908	↑	0.012
7.608	pos	Isoformononetin	1.961	6.108	↑	0.003
7.219	pos	Quillaic acid 3-[galactosyl-(1->2)-glucuronide]	1.958	11.587	↑	0.008
13.417	pos	Carcinine	1.955	1.863	↑	0.016
11.347	pos	(-)-Malyngolide	1.941	2.229	↑	0.004
13.606	pos	3-Methyl-3-heptene	1.931	1.530	↑	0.047
2.774	pos	1-Hexadecanol	1.926	1.835	↑	0.012
7.489	pos	Glycocholic acid	1.919	4.542	↑	0.028
7.099	pos	Tenuifolin	1.906	2.637	↑	0.026
4.485	neg	Apigenin 7-O-diglucuronide	1.905	9.471	↑	0.008
4.831	pos	Soyasaponin A2	1.904	7.574	↑	0.035
8.159	pos	Cucurbitacin I 2-glucoside	1.897	11.434	↑	0.011
12.211	neg	MG(20:4(8Z,11Z,14Z,17Z)/0:0/0:0)	1.894	1.512	↑	0.039
1.271	pos	3,7,12-Trioxochola-1,4-dien-24-oic Acid	1.879	1.387	↑	0.012
10.664	neg	Macrophyllol A (flavone)	1.863	2.097	↑	0.020
4.132	pos	Heptaethylene glycol	1.849	1.622	↑	0.041
12.643	pos	Dihydroouabain	1.847	3.426	↑	0.014
5.419	neg	Hovenoside I	1.846	5.476	↑	0.007
12.643	pos	20-Hydroxy-leukotriene E4	1.843	1.954	↑	0.011
0.752	neg	FAPy-adenine	1.839	1.731	↑	0.016
5.701	pos	Soyasaponin V	1.807	14.760	↑	0.008
10.928	neg	Anguvetin	1.806	1.831	↑	0.007
11.558	pos	Chenodeoxycholylasparagine	1.803	1.450	↑	0.047
10.770	neg	Irene	1.789	1.572	↑	0.018
12.652	neg	11-(Dansylamino)undecanoic acid	1.785	1.407	↑	0.049
10.967	pos	12-HETE	1.769	1.913	↑	0.009
13.008	pos	Perflutren	1.766	1.771	↑	0.049
12.817	pos	13-Heptadecyn-1-ol	1.764	1.708	↑	0.017
8.075	pos	LPIM2(16:0/0:0)	1.760	24.286	↑	0.009
12.643	pos	20-Oxo-leukotriene E4	1.759	2.014	↑	0.016
12.364	pos	5alpha-Androsta-16-ene-3-ol	1.753	5.529	↑	0.018
4.557	neg	6S,9R-Dihydroxy-4,7E-megastigmadien-3-one 9-[apiosyl-	1.715	4.498	↑	0.007
3.862	pos	Epsilon-caprolactam	1.715	1.579	↑	0.028
10.227	neg	Palomid 529	1.706	1.744	↑	0.029
11.523	pos	1-O-(2R-hydroxy-tetradecyl)-sn-glycerol	1.704	1.813	↑	0.016
10.793	pos	cis-Palmitvaccenic acid	1.691	1.657	↑	0.009
1.215	neg	6-Formylindolo [3,2-B] carbazole	1.687	1.522	↑	0.041
8.400	pos	1-(2,6,6-Trimethyl-2-cyclohexen-1-yl)-1,6-heptadien-3-	1.687	1.636	↑	0.022
5.596	neg	Medinoside E	1.685	12.837	↑	0.024
12.433	neg	1,4-Dimethyl-7-ethylazulene	1.677	2.090	↑	0.048
12.973	pos	14,15-EE-8(Z)-E	1.673	2.247	↑	0.005
5.060	pos	Kaempferol 3-methyl ether 7-glucuronide	1.650	61.012	↑	0.032

8.759	neg	Camicinal	1.641	1.675	↑	0.019
12.643	pos	bhas#22	1.635	1.915	↑	0.008
8.989	neg	3-Hydroxy-4-aminopyridine sulfate	1.631	1.535	↑	0.032
4.717	pos	Tricin 7-[feruloyl-(->2)-glucuronyl-(1->2)-[glucuronyl-	1.617	11.456	↑	0.039
9.475	pos	7Z-hexadecen-1-ol	1.614	1.558	↑	0.036
8.751	pos	Soyasaponin IV	1.613	2.932	↑	0.009
7.851	pos	8-O-Methylretusin	1.605	4.876	↑	0.004
11.209	pos	PKODiA-PA	1.602	2.487	↑	0.006
9.504	neg	Methylpyrogallol sulfate 3	1.598	1.503	↑	0.037
5.904	pos	26-(2-Glucosyl-6-acetylglucosyl]-1,3,11,22-	1.595	9.920	↑	0.006
6.385	pos	Ampeloside Bs1	1.590	4.445	↑	0.026
8.768	pos	Emetine	1.587	1.754	↑	0.038
13.799	neg	Heptafluorobutyric acid	1.585	1.463	↑	0.045
8.056	neg	3-[(1R,2R,3S)-3-Carboxy-1,2,3-	1.577	1.365	↑	0.049
10.241	pos	DG(19:0/0:0/20:5(7Z,9Z,11E,13E,17Z)-3OH(5,6,15))	1.577	2.338	↑	0.005
13.229	pos	2',3'-Didehydro-2',3'-dideoxycytidine	1.575	1.766	↑	0.006
4.450	neg	Hexafluoropropene	1.567	1.618	↑	0.036
9.729	pos	13-HODE-EA	1.562	1.828	↑	0.011
8.759	neg	Veranisatin A	1.555	1.890	↑	0.018
10.687	pos	5-Oxo-6,8,11,14-eicosatetraenoic acid	1.551	2.129	↑	0.025
9.913	pos	Butylparaben	1.546	1.661	↑	0.026
11.332	neg	9S-hydroxy-7E-hexadecenoic acid	1.545	1.761	↑	0.023
7.851	pos	6-((8Z,11Z,14Z)-heptadeca-8,11,14-trien-1-yl)salicylic	1.539	3.590	↑	0.013
6.290	neg	28-Glucosylarjunolate 3-[rhamnosyl-(1->3)-glucuronide]	1.517	8.288	↑	0.021
8.159	pos	3beta-3-Hydroxy-18-lupen-21-one	1.514	5.590	↑	0.037
3.578	pos	Pentanamide	1.513	2.023	↑	0.017
13.332	pos	Diphenylamine	1.497	1.846	↑	0.044
6.397	neg	Medicoside G	1.494	5.852	↑	0.008
11.000	pos	21-oxo-docosanoic acid	1.491	1.463	↑	0.040
9.645	neg	Piridronic acid	1.491	1.474	↑	0.037
9.760	pos	3alpha,12alpha-Dihydroxy-5beta-chola-7,14-dien-24-oic	1.484	4.824	↑	0.001
8.799	pos	Methymycin	1.482	2.091	↑	0.004
8.991	pos	Diethyl phthalic acid	1.477	1.591	↑	0.034
10.879	pos	8,8-Diethoxy-2,6-dimethyl-2-octanol	1.476	1.995	↑	0.014
6.629	neg	2-Phenylpyrazolo(4,3-c)quinolin-3(5H)-one	1.475	2.282	↑	0.042
14.313	pos	Secnidazole	1.473	1.728	↑	0.024
12.660	pos	7beta-Hydroxy-12-oxo-5alpha-cholan-24-oic Acid	1.455	1.923	↑	0.002
12.222	pos	(R)-4A-(Ethoxymethyl)-1-(4-fluorophenyl)-6-((4-	1.446	2.139	↑	0.012
9.760	pos	PG(5-iso PGF2VI/i-13:0)	1.438	1.491	↑	0.005
8.522	pos	cis-10-palmitoleic acid	1.437	1.516	↑	0.047
8.627	pos	Ophioxanthin	1.434	8.128	↑	0.044
9.440	pos	3,4',5,6,7-PENTAMETHOXYFLAVONE	1.433	46.244	↑	0.027
10.946	neg	OSU03012	1.432	1.770	↑	0.005
14.313	pos	Alanylhydroxyproline	1.431	1.859	↑	0.020

10.326	pos	LysoPE(0:0/20:4(8Z,11Z,14Z,17Z))	1.421	6.266	↑	0.043
13.178	pos	n-arachidonylethanolamine	1.417	2.094	↑	0.034
8.917	neg	Dehydrosoyasaponin I	1.417	7.251	↑	0.031
9.235	pos	N-Myristoyl Glutamic acid	1.417	4.226	↑	0.008
4.416	neg	Sordarin	1.413	4.018	↑	0.007
10.227	neg	5-Amino-1-[(2R,4S,5R)-3,4-dihydroxy-5-	1.410	1.670	↑	0.028
4.048	pos	N-Benzoylaspartic acid	1.403	13.638	↑	0.002
8.775	neg	Perindopril Acyl-beta-D-glucuronide	1.401	3.629	↑	0.008
11.035	pos	Tris(2-butoxyethyl) phosphate	1.391	1.600	↑	0.022
8.784	pos	Palmitoyl Ara-C	1.384	1.825	↑	0.037
5.295	neg	Perfluoroisobutylene	1.375	1.550	↑	0.038
7.186	pos	n-Formyl-methionyl-leucyl-phenyl-alanine	1.375	1.963	↑	0.022
13.042	pos	Isolinderenolide	1.362	10.495	↑	0.004
8.092	pos	Digitoxigenin bisdigitoxide	1.360	25.578	↑	0.002
5.768	pos	Bancroftinone	1.360	1.802	↑	0.013
14.259	pos	Gentisic acid	1.356	1.843	↑	0.022
8.759	neg	PI(20:3(8Z,11Z,14Z)/22:4(7Z,10Z,13Z,16Z))	1.355	1.852	↑	0.024
14.331	pos	Ectoine	1.349	1.860	↑	0.018
13.417	pos	Vaccenic acid	1.347	1.524	↑	0.001
10.932	pos	Methysergide	1.346	1.857	↑	0.046
4.539	neg	Sulfamethoxazole N1-glucuronide	1.340	8.614	↑	0.018
3.695	pos	Acipimox	1.318	1.648	↑	0.035
10.687	pos	Piroxantrone	1.311	2.272	↑	0.018
5.055	neg	Benzoylmalic acid	1.303	31.154	↑	0.035
8.002	neg	Dimethyl 2,3-bis(sulfanyl)butanedioate	1.303	1.444	↑	0.041
12.366	neg	DG(20:4(5Z,8Z,11Z,14Z)-OH(17)/i-16:0/0:0)	1.302	4.255	↑	0.048
7.162	neg	5-Fluorouridine monophosphate	1.297	1.352	↑	0.048
4.574	neg	Myricetin 3-O-(4''-O-acetyl-2''-O-galloyl)-alpha-L-	1.292	11.508	↑	0.006
4.559	pos	N-decanoyl histidine	1.292	4.711	↑	0.010
10.241	pos	3beta,7alpha-Dihydroxychol-5-en-24-oic Acid	1.290	2.549	↑	0.001
0.768	pos	2-Phenylethyl octanoate	1.284	1.749	↑	0.016
8.505	pos	1,3-Dicyclohexylurea	1.281	1.578	↑	0.034
5.684	neg	Trillfurostanoside B	1.281	3.479	↑	0.050
9.677	pos	Berkeleylactone L	1.271	1.564	↑	0.022
13.042	pos	2-Diethylaminoethanol	1.271	1.608	↑	0.032
6.718	neg	m-Chlorobenzoic acid	1.268	1.587	↑	0.032
12.660	pos	7-O-(4-Hydroxycinnamoyl) astragalin	1.261	2.055	↑	0.014
13.452	pos	N-Ethyl trans-2-cis-6-nonadienamide	1.258	1.664	↑	0.029
5.012	pos	PGP(18:0/22:4(7Z,10Z,13Z,16Z))	1.255	7.779	↑	0.023
5.331	neg	Araliasaponin III	1.254	13.025	↑	0.025
4.834	neg	Methyl dioxindole-3-acetate	1.249	6.587	↑	0.003
6.947	pos	Undecylenic acid	1.248	1.161	↑	0.033
1.886	pos	3-Diphosphoglyceric acid	1.246	1.685	↑	0.040
10.775	pos	1-Octadecene	1.246	1.799	↑	0.012

4.627	neg	5-Hydroxyferulic acid	1.244	1.437	↑	0.036
13.042	pos	3-oxo-heneicosanoic acid	1.242	5.976	↑	0.004
4.627	neg	Lirodenine	1.240	9.334	↑	0.025
5.464	pos	3-Hydroxynona-4,6-dienoylcarnitine	1.240	1.838	↑	0.029
4.880	pos	Tricin 7-[rhamnosyl-(1->2)-galacturonide]	1.240	7.780	↑	0.044
9.373	pos	Mono-(2-ethyl-5-hydroxyhexyl) phthalate	1.240	1.814	↑	0.022
4.988	neg	cephalosporanic acid	1.234	45.761	↑	0.039
12.433	neg	N-Oleoyl Asparagine	1.228	2.000	↑	0.035
8.834	pos	Nobiletin	1.225	18.247	↑	0.032
5.895	neg	Sufotidine	1.224	7.452	↑	0.009
10.687	pos	Bapta	1.224	2.530	↑	0.019
10.258	pos	MG(18:1(9Z)-O(12,13)/0:0/0:0)	1.211	1.564	↑	0.019
7.587	neg	Dihydrowogonin	1.210	5.118	↑	0.003
9.628	neg	Methyl 3,5-dinitrobenzoate	1.209	1.483	↑	0.027
8.741	neg	Declopramide	1.208	1.786	↑	0.027
10.567	pos	oscr#25	1.206	3.636	↑	0.009
9.027	pos	1-Hexadecene	1.206	1.646	↑	0.031
12.652	neg	Tert-Butyl (4-(3-((7-(hydroxyamino)-7-	1.205	1.418	↑	0.047
4.542	pos	Amobarbital	1.205	3.180	↑	0.036
10.051	neg	AS-252424	1.203	1.459	↑	0.048
13.212	pos	Octa-3,5-dienoylcarnitine	1.199	1.618	↑	0.009
7.179	neg	Chitotriose	1.197	1.709	↑	0.012
13.717	neg	Barbituric acid	1.197	1.593	↑	0.020
0.987	pos	Vinylphosphonic acid	1.193	1.646	↑	0.037
3.695	pos	Proversilin D	1.190	1.795	↑	0.035
14.192	pos	2-Amino-4-methylpyridine	1.187	1.761	↑	0.024
10.652	pos	2-[3]-ladderane ethanoic acid	1.180	3.289	↑	0.005
7.821	neg	Pentadecanoylglycine	1.179	4.090	↑	0.045
8.723	neg	Pitheduloside A	1.177	2.637	↑	0.025
4.238	neg	Sialorphin	1.173	3.497	↑	0.037
12.643	pos	3-[2-(4-Fluorophenyl)ethylamino]-1-methyl-4-(2-methyl-	1.170	1.993	↑	0.019
6.248	pos	Sodium nitrate (NaNO3)	1.170	2.001	↑	0.031
1.055	neg	1H-Pyrazolo[3,4-d]pyrimidin-4-amine	1.168	1.559	↑	0.021
5.580	pos	Cucurbitacin F	1.168	2.864	↑	0.049
9.305	pos	alpha-Irone	1.166	1.631	↑	0.012
4.309	neg	Swertiamarin	1.166	5.523	↑	0.012
9.643	pos	Cladosporin	1.162	1.994	↑	0.013
8.768	pos	PG(i-22:0/20:5(5Z,8Z,11Z,14Z,16E)-OH(18))	1.159	1.862	↑	0.037
12.435	pos	Pracinostat	1.158	3.468	↑	0.010
10.983	pos	Avocadyne 4-acetate	1.154	2.584	↑	0.001
7.838	neg	Lucidenic acid L	1.153	3.438	↑	0.012
12.678	pos	Dihomolinoleic acid	1.152	1.563	↑	0.020
9.291	neg	methyl 8-[2-(2-formyl-vinyl)-3-hydroxy-5-oxo-	1.142	1.594	↑	0.033
5.546	pos	N-(p-Hydroxyphenyl)ethyl p-hydroxycinnamide	1.140	2.511	↑	0.012

1.686	pos	3-oxo-4-pentenoic acid	1.139	1.634	↑	0.030
10.278	neg	Urolithin-3-sulfate	1.136	1.481	↑	0.049
6.042	pos	Pratensein	1.131	42.058	↑	0.001
10.296	neg	5,10-Pentadecadien-1-ol	1.127	1.768	↑	0.024
13.264	pos	(1Ar,3S,4aS,7S,8aS)-3-[(1S,2R,3R)-3-[(2R,5S)-5,6-	1.113	1.856	↑	0.008
10.949	pos	Goshuyic acid	1.110	1.723	↑	0.007
10.967	pos	Valtratum	1.109	1.949	↑	0.015
1.215	neg	2-Oxazolidinone, 3-[[(5-nitro-2-	1.107	1.474	↑	0.009
7.055	neg	Quillaic acid 3-[rhamnosyl-(1->3)-[galactosyl-(1->2)]-	1.107	13.738	↑	0.028
10.261	neg	1-(2-Pyridylazo)-2-naphthol	1.107	2.581	↑	0.032
10.123	pos	triacetylresveratrol	1.101	2.114	↑	0.014
10.828	pos	3,4-Dimethyl-5-pentyl-2-furanpentanoic acid	1.096	1.682	↑	0.020
12.834	pos	DG(15:0/0:0/15:0) (d5)	1.093	1.588	↑	0.028
6.415	neg	2,4-Dinitrophenol	1.093	1.639	↑	0.049
8.784	pos	PGP(18:3(9,11,15)-OH(13)/i-21:0)	1.093	5.046	↑	0.002
2.958	pos	9-Decenal	1.092	1.507	↑	0.032
4.897	pos	Chapso	1.087	22.919	↑	0.006
5.155	pos	2-[2-(2-Butoxyethoxy)ethoxy]ethanol	1.086	1.671	↑	0.042
3.494	pos	2-Amino-3-(2-fluoro-3,4-dihydroxyphenyl)propanoic acid	1.085	2.032	↑	0.015
11.840	pos	Palmitoylcarnitine	1.076	1.712	↑	0.028
9.733	neg	(±)-(E)-3-Methyl-4-decen-1-ol	1.074	1.599	↑	0.035
7.270	pos	Nb-Feruloyltryptamine	1.070	5.564	↑	0.015
10.227	neg	DG(18:0/20:5(5Z,8Z,11Z,14Z,16E)-OH(18R)/0:0)	1.068	2.017	↑	0.022
11.209	pos	[6-(5-Chloropyridin-2-yl)-7-hydroxypyrrolo[3,4-	1.063	1.663	↑	0.042
4.098	pos	Mauritine A	1.061	13.490	↑	0.014
8.759	neg	Sulfated Dihydromenaquinone-9	1.051	1.840	↑	0.015
4.559	pos	Enol-phenylpyruvate	1.048	2.786	↑	0.010
3.023	pos	3',5'-Dideoxythymidine	1.043	1.725	↑	0.015
12.576	pos	Denticulaflavonol	1.042	1.926	↑	0.015
7.203	pos	8,9-Dihydro-5-hydroxy-8-(1-hydroxy-1-methylethyl)-6-(2-	1.041	2.477	↑	0.013
2.985	neg	[(2S,3R,5S,6S)-2,3,4,5,6-Pentaphosphonooxycyclohexyl]	1.035	1.885	↑	0.041
10.532	pos	(R)-4-(2-(2-(2-Methylpyrrolidin-1-yl)ethyl)benzofuran-5-	1.033	1.569	↑	0.026
9.398	neg	PI(22:0/0:0)	1.026	4.810	↑	0.001
9.999	pos	14R-hydroxy-11E-eicosenoic acid	1.021	1.619	↑	0.023
5.820	pos	PGP(i-15:0/LTE4)	1.018	12.549	↑	0.022
14.745	pos	N-Stearoyl Lysine	1.016	1.666	↑	0.037
6.180	pos	3b-Pregnadienolone 3-[rhamnosyl-(1->4)-rhamnosyl-	1.015	11.399	↑	0.028
8.759	neg	LysoPE(0:0/22:6(4Z,7Z,10Z,13Z,16Z,19Z))	1.009	2.186	↑	0.019
6.700	neg	(2S,5R)-3,3-Dimethyl-2-(2-phenylethyl)-4-thia-1-	1.006	8.537	↑	0.033

Table S9 Significantly different metabolites in A1A1 cows and A2A2 cows

Retention	Ion	Metabolites	VIP	FC	Regulation	*P*-value
13.088	neg	Adrenic acid	5.160	0.558	↓	0.040

8.075	pos	LPIM2(16:0/0:0)	4.147	0.296	↓	0.046
5.529	pos	Astemizole	3.961	0.472	↓	0.045
4.963	pos	5-Megastigmen-7-yne-3,9-diol 3-glucoside	3.202	0.474	↓	0.041
5.498	pos	Foretinib	3.200	0.472	↓	0.042
5.615	pos	PG(18:3(9Z,12Z,15Z)/0:0)	3.041	0.290	↓	0.007
4.609	neg	Daidzin	2.990	0.249	↓	0.030
7.456	pos	Cibaric acid	2.893	0.622	↓	0.006
12.206	pos	Oleoylethanolamide	2.863	0.632	↓	0.022
7.269	neg	(-)-11-Hydroxy-9,15,16-trioxooctadecanoic acid	2.852	0.741	↓	0.022
4.953	neg	pelargonidin-3-O-beta-D-glucoside	2.848	0.258	↓	0.022
11.000	pos	Alpha-Linolenoyl ethanolamide	2.462	0.681	↓	0.013
4.292	neg	Nifekalant	2.430	0.421	↓	0.038
6.522	neg	Taurocholic acid	2.300	0.306	↓	0.032
0.597	pos	dCMP	2.249	0.219	↓	0.026
4.332	pos	Vomifoliol	2.209	0.637	↓	0.036
3.965	pos	(1R,2R,4S)-p-Menthane-1,2,8-triol 8-glucoside	2.162	0.418	↓	0.038
7.875	neg	11-Dihydro-12-norneoquassin	1.971	0.640	↓	0.013
8.165	neg	Abrusoside B	1.963	0.262	↓	0.050
9.729	pos	13-HODE-EA	1.862	0.571	↓	0.038
8.056	neg	Imiglitazar	1.809	0.258	↓	0.040
4.031	pos	4-O-alpha-D-Galactopyranosylcalystegine B2	1.757	0.232	↓	0.047
5.542	neg	Polysorbate 80	1.725	0.393	↓	0.046
3.627	pos	Atenolol	1.697	0.427	↓	0.014
4.216	pos	Methylnissolin 3-O-glucoside	1.676	0.514	↓	0.045
5.613	neg	Fluocortolone Pivalate	1.551	0.346	↓	0.017
9.166	pos	Deoxycholylglycine	1.545	0.350	↓	0.043
6.144	pos	Trichocarposide	1.521	0.358	↓	0.006
4.681	neg	ascr#4	1.517	0.386	↓	0.029
4.930	pos	(7'R,8'R)-4,7'-Epoxy-3',5-dimethoxy-4',9,9'-lignanetriol 9'-	1.514	0.415	↓	0.028
0.597	pos	Diazoxon	1.511	0.212	↓	0.024
4.963	pos	N-(2,5-Dimethoxybenzyl)-N-(5-fluoro-2-	1.497	0.366	↓	0.038
4.627	neg	Genistein 7-O-glucoside-6''-malonate	1.488	0.228	↓	0.040
0.582	pos	Ovalitenin C	1.476	0.306	↓	0.048
1.770	pos	Monomethyl glutaric acid	1.468	0.679	↓	0.022
5.580	pos	Gibberellin A7	1.439	0.427	↓	0.041
4.292	neg	3b,6a-Dihydroxy-alpha-ionol 9-[apiosyl-(1->6)-glucoside]	1.422	0.459	↓	0.041
8.651	neg	1-tauro-dinor-PGE1	1.410	0.278	↓	0.044
0.754	pos	L-Lysine	1.375	0.657	↓	0.026
4.733	pos	Isorhamnetin 3-O-[b-D-glucopyranosyl-(1->2)-a-L-	1.363	0.211	↓	0.036
5.701	neg	Cyclokievitone hydrate	1.360	0.544	↓	0.048
5.718	pos	6,8-Di-C-methylkaempferol 3,7-dimethyl ether	1.329	0.576	↓	0.044
4.184	neg	Ladirubicin	1.297	0.429	↓	0.032
5.683	pos	kaempferol 7-O-neohesperidoside	1.294	0.490	↓	0.029
6.454	pos	Secundifloran	1.288	0.548	↓	0.017

0.628	pos	(4R,5S)-4-Hydroxy-3,4,5-trimethyl-5,6,7,8-tetrahydronaphtho[2,3-	1.226	0.206	↓	0.029
9.062	pos	Prostaglandin A2	1.218	0.440	↓	0.020
3.966	neg	(1S,2R,4R,8S)-p-Menthane-2,8,9-triol 9-glucoside	1.217	0.419	↓	0.041
8.075	pos	A6 Peptide	1.195	0.255	↓	0.027
7.948	neg	Sambutoxin	1.186	0.411	↓	0.020
4.048	pos	Robinetinidol-4alpha-ol	1.182	0.387	↓	0.031
5.768	pos	Musabalbisiane C	1.178	0.482	↓	0.033
4.521	neg	Diffusoside C	1.175	0.463	↓	0.046
7.851	pos	2-Hydroxyestradiol	1.172	0.636	↓	0.006
5.754	neg	Methyl 4-[(E)-2-(5,5,8,8-tetramethyl-6,7-dihydronaphthalen-2-	1.165	0.440	↓	0.042
6.486	neg	hemsleyanoside	1.154	0.463	↓	0.020
10.997	neg	3-Hydroxy-cis-5-tetradecenoylcarnitine	1.149	0.556	↓	0.005
6.576	pos	isoorientin 6-O-hexoside	1.149	0.472	↓	0.028
5.313	neg	Paromomycin	1.139	0.391	↓	0.031
3.222	pos	Caffeoylmalic acid	1.117	0.458	↓	0.037
4.913	pos	Foeniculoside IX	1.114	0.562	↓	0.041
9.795	pos	N-Palmitoyl Lysine	1.089	0.289	↓	0.031
5.125	pos	Madreselvin B	1.080	0.356	↓	0.048
8.433	neg	Cladosporacid D	1.068	0.532	↓	0.004
4.902	neg	(R)-1-O-b-D-glucopyranosyl-1,3-octanediol	1.058	0.422	↓	0.040
6.385	pos	chrysoeriol 7-O-neohesperidoside	1.047	0.483	↓	0.025
4.627	neg	Riboflavin cyclic-4',5'-phosphate	1.046	0.378	↓	0.047
6.646	neg	2-Hydroxy-3-methyl-4H-pyran-4-one O-(6E-cinnamoyl-b-D-	1.038	0.441	↓	0.048
4.953	neg	6''-Malonylcosmosiin	1.008	0.262	↓	0.042
8.698	pos	Sundiversifolide	1.006	0.530	↓	0.027
4.818	neg	3-(3-Hydroxyphenyl)propanoic acid	15.707	3.438	↑	0.043
8.314	pos	5,8,12-Trihydroxy-9-octadecenoic acid	15.100	1.829	↑	0.016
10.585	pos	LysoPE(0:0/16:0)	12.238	2.763	↑	0.028
4.332	pos	(3b,4b,11b,14b)-11-Ethoxy-3,4-epoxy-14-hydroxy-12-cyathen-15-	12.058	3.435	↑	0.028
5.966	neg	Americanol A	10.570	3.688	↑	0.033
1.267	neg	Inosine	9.798	3.457	↑	0.017
1.192	pos	Adenosine	8.126	2.219	↑	0.035
4.381	neg	Thonzylamine	7.219	5.479	↑	0.040
4.057	neg	4-(2-Aminophenyl)-2,4-dioxobutanoic acid	6.723	6.571	↑	0.034
4.521	neg	2-(3-Hydroxy-1H-indol-2-yl)acetic acid	5.930	4.644	↑	0.031
4.818	neg	Quercetin 3-O-sophoroside	5.869	5.556	↑	0.037
12.730	pos	FAHFA(16:0/14-O-18:0)	5.801	2.265	↑	0.048
5.430	pos	Dehydromatricaric acid	5.472	4.365	↑	0.030
14.290	neg	Schisanhenol	5.361	1.710	↑	0.018
4.588	pos	7-Epi-12-hydroxyjasmonic acid	5.188	1.809	↑	0.050
5.295	neg	Gibberellin A59	4.915	2.948	↑	0.025
9.878	pos	LysoPE(15:0/0:0)	4.804	2.558	↑	0.046
1.267	neg	Glucose pyruvate lactate	4.584	2.827	↑	0.017
12.049	pos	Glycyrrhetol	4.040	1.718	↑	0.048

4.818	neg	oscr#7	3.975	3.168	↑	0.033
11.717	pos	PE-Cer(d14:2(4E,6E)/16:0(2OH))	3.943	3.241	↑	0.008
1.644	neg	Baohuoside I	3.760	3.846	↑	0.023
9.878	pos	(2R,3R)-2-Aminooctadecane-1,3-diol	3.651	2.584	↑	0.039
4.767	pos	Ceranapril	3.588	2.228	↑	0.047
8.869	pos	24-Nor-5beta-cholane-3alpha,7alpha,12alpha-triol	3.571	11.090	↑	0.047
9.760	pos	PG(5-iso PGF2VI/i-13:0)	3.500	1.543	↑	0.003
10.192	pos	N-(m-Methoxybenzyl)hexadecanamide	3.443	11.256	↑	0.008
10.175	pos	LysoPE(0:0/15:0)	3.421	3.089	↑	0.004
4.542	pos	Vignatic acid B	3.345	4.732	↑	0.025
9.744	pos	LysoPE(14:0/0:0)	3.317	3.910	↑	0.008
1.231	neg	Guanosine	3.268	1.804	↑	0.026
4.527	pos	Estriol-16-Glucuronide	3.245	27.680	↑	0.045
0.768	pos	Acetophenazine	3.178	2.752	↑	0.033
1.398	pos	Noralfentanil	3.163	9.568	↑	0.015
1.231	neg	True blue	3.137	4.337	↑	0.026
4.669	pos	Linagliptin	3.130	2.904	↑	0.049
7.465	neg	bhos#42	3.088	6.324	↑	0.015
12.312	pos	Momorcharaside B	3.057	2.825	↑	0.005
4.801	neg	Methyl helianthenoate A glucoside	3.032	3.223	↑	0.032
1.321	neg	9H-Purine-9-butanoic acid, 6-amino-alpha-hydroxy-, methyl ester	2.958	2.279	↑	0.032
7.465	neg	Dimethindene	2.950	2.971	↑	0.035
9.339	pos	Dehydrophytosphingosine	2.833	2.549	↑	0.018
0.739	pos	Daunosamine	2.820	1.653	↑	0.010
0.768	pos	Fructose lactate	2.819	2.505	↑	0.013
8.326	neg	2,15-dihydroxy-pentadecylic acid	2.785	2.446	↑	0.028
3.140	pos	Procaine	2.772	2.954	↑	0.035
12.540	pos	PE-Cer(d14:2(4E,6E)/16:0)	2.766	9.302	↑	0.007
10.035	pos	Sphingosine	2.763	4.099	↑	0.014
5.701	neg	Enterodiol	2.738	2.427	↑	0.031
0.769	neg	Elephantorrhizol	2.710	2.532	↑	0.022
3.425	pos	TIADENOL	2.675	3.268	↑	0.031
9.475	pos	Phytosphingosine	2.673	1.586	↑	0.026
11.173	neg	LysoPS(16:0/0:0)	2.647	4.954	↑	0.008
11.712	neg	Iprovalicarb	2.609	3.383	↑	0.013
7.456	pos	DG(20:5(5Z,8Z,11Z,14Z,17Z)/22:6(4Z,7Z,10Z,13Z,16Z,19Z)/0:0)	2.608	4.159	↑	0.022
4.381	neg	Cyclohexyladenosine	2.587	11.072	↑	0.019
12.696	pos	PE-Cer(d14:2(4E,6E)/18:0(2OH))	2.576	2.786	↑	0.012
5.172	pos	Hv-NCC-1	2.533	3.875	↑	0.043
1.464	neg	4-Pyridoxic acid	2.501	3.193	↑	0.030
5.966	neg	o-Cresolphthalein complexone	2.491	27.445	↑	0.024
4.801	neg	19-Noraldosterone	2.456	7.016	↑	0.039
9.847	pos	1alpha-hydroxy-24,25,26,27-tetranorvitamin D3 / 1alpha-hydroxy-	2.446	4.719	↑	0.024
4.527	pos	4-Hydroxyquinoline	2.406	3.165	↑	0.043

10.227	neg	PA(0:0/16:0)	2.383	2.914	↑	0.020
4.416	neg	Pisumic acid	2.373	3.216	↑	0.039
4.057	neg	Orotidine	2.353	18.911	↑	0.019
12.031	pos	Lysocellin	2.271	6.591	↑	0.005
9.627	pos	Clupadonic acid	2.236	11.091	↑	0.019
8.211	pos	Ganoderenic acid C	2.225	3.525	↑	0.017
10.361	pos	LysoPS(18:2(9Z,12Z)/0:0)	2.218	3.295	↑	0.029
4.521	neg	Glucosamine 6-phosphate	2.172	2.982	↑	0.044
11.646	pos	Soyasapogenol E	2.152	2.558	↑	0.025
5.296	pos	7-Hydroxyenterolactone	2.145	4.219	↑	0.032
1.197	neg	4'-Azidocytidine	2.132	2.657	↑	0.007
7.287	pos	O-Desmethylangolensin	2.087	2.339	↑	0.010
1.267	neg	Alginic acid	2.081	2.847	↑	0.017
4.349	pos	3-O-acetylecdysone 2-phosphate	2.079	7.772	↑	0.025
4.557	neg	p-Cresol sulfate	2.073	2.823	↑	0.043
7.473	pos	Neofusapyrone	2.069	3.679	↑	0.043
5.177	neg	Xamoterol	2.016	4.547	↑	0.034
6.486	neg	3-(1,1-Dimethylallyl)herniarin	1.991	3.087	↑	0.047
4.834	neg	Ustusoic acid A	1.990	8.772	↑	0.037
7.987	pos	(3R,5R)-7-[(1S,2R,3R,8S,8Ar)-3-hydroxy-2-methyl-8-(2-	1.985	5.180	↑	0.028
4.818	neg	18-Oxocortisol	1.983	12.431	↑	0.034
6.841	neg	14S-hydroxy-hexadecanoic acid	1.966	3.347	↑	0.019
13.248	pos	N-oleoyl isoleucine	1.946	2.365	↑	0.040
0.723	pos	Fudosteine	1.940	3.222	↑	0.028
3.222	pos	4-Amino-2,2,6,6-tetramethylpiperidin-1-ol	1.932	4.721	↑	0.040
7.448	neg	N-Lauroyl Cysteine	1.926	4.564	↑	0.023
14.290	neg	Diospyrin	1.925	2.004	↑	0.005
11.471	pos	Triethylene glycol bis(3-tert-butyl-4-hydroxy-5-	1.896	12.862	↑	0.017
5.430	pos	Cinnamamide	1.894	2.209	↑	0.016
5.718	pos	2,3-Bis(3-hydroxybenzyl)butane-1,4-diol	1.887	2.520	↑	0.030
4.850	neg	9,10-Dihydro-8-hydroxy-10-methyl-8H-pyrano[2,3-h]epicatechin	1.883	2.919	↑	0.046
11.823	pos	N-Acetylsphinganine	1.867	2.494	↑	0.020
6.692	pos	Equol	1.861	1.864	↑	0.044
9.733	neg	Sphingosine 1-phosphate	1.850	3.007	↑	0.012
4.327	neg	2-Oxo-turkesterone	1.817	3.496	↑	0.030
5.055	neg	3-[7-[3-(4-Acetyl-3-hydroxy-2-propylphenoxy)-2-	1.816	11.395	↑	0.011
8.129	neg	Citpressine I	1.815	1.350	↑	0.012
3.391	pos	Benzoyl L-arginine methyl ester	1.804	4.479	↑	0.041
5.837	pos	5,4'-Dihydroxy-6-C-prenylflavanone 4'-xylosyl-(1->2)-rhamnoside	1.797	3.302	↑	0.050
9.913	pos	LysoPE(0:0/16:1(9Z))	1.790	2.841	↑	0.012
1.215	neg	Oxodipine	1.754	2.073	↑	0.024
10.718	neg	PA(2:0/PGJ2)	1.749	5.432	↑	0.046
1.267	neg	Triazophos	1.748	17.605	↑	0.021
11.120	neg	LysoPA(P-16:0/0:0)	1.690	2.310	↑	0.027

4.521	neg	2-Methoxy-5-(2,4-dioxo-5-thiazolidinyl)-N-((4-	1.687	9.813	↑	0.034
5.826	neg	Lariciresinol-sesquilignan	1.674	3.707	↑	0.045
8.183	neg	Ganoderic acid A	1.671	3.658	↑	0.017
6.316	pos	(E)-5,8-Megastigmadien-4-one	1.663	1.405	↑	0.008
4.521	neg	Indole-3-carbinol	1.652	2.422	↑	0.029
5.615	pos	Aflatoxin ExB2	1.650	4.130	↑	0.041
5.966	neg	Epipodophyllotoxin derivitive	1.639	3.278	↑	0.049
4.527	pos	Phenylacetylglycine	1.638	2.079	↑	0.031
3.915	pos	Centpropazine	1.612	2.009	↑	0.043
9.574	neg	(3beta,8beta)-3-Hydroxy-7(11)-eremophilen-12,8-olide	1.610	2.467	↑	0.037
5.966	neg	Moschamindole	1.558	3.308	↑	0.032
4.588	pos	3alpha,7alpha,12alpha-trihydroxy-5alpha-cholan-24-yl sulfate	1.549	2.482	↑	0.022
5.295	neg	6-Deoxy-8-O-methylrabelomycin; 3-Hydroxy-8-methoxy-3-	1.542	3.313	↑	0.033
2.194	pos	5-Aminopentanoic acid	1.536	1.189	↑	0.030
4.256	neg	1-O-p-Coumaroyl-beta-D-glucose	1.527	4.147	↑	0.027
1.267	neg	Sulfacetamide	1.527	2.541	↑	0.020
4.785	neg	Alminoprofen	1.525	3.139	↑	0.049
4.382	pos	Deferitazole	1.512	4.532	↑	0.049
2.194	pos	(-)-Slaframine	1.508	2.236	↑	0.010
13.212	pos	DG(20:3(8Z,11Z,14Z)-2OH(5,6)/i-13:0/0:0)	1.507	2.495	↑	0.008
5.718	pos	16-Oxoestrone	1.483	2.854	↑	0.022
10.682	neg	Maslinic acid	1.480	2.074	↑	0.048
0.597	pos	Agmatine	1.475	5.522	↑	0.034
0.644	pos	L-Asparagine	1.448	3.170	↑	0.040
1.267	neg	Sinalbin A	1.442	2.520	↑	0.007
0.768	pos	AFP 07 (free acid)	1.438	2.676	↑	0.001
11.717	pos	Officinoterpenoside C	1.405	4.342	↑	0.018
9.812	pos	Glabric acid	1.405	1.753	↑	0.030
0.754	pos	Atglistatin	1.398	4.365	↑	0.031
13.220	neg	Glucocorticoid receptor agonist	1.384	1.407	↑	0.013
4.559	pos	Dibudipine	1.384	6.008	↑	0.011
4.818	neg	2,3-Dimethylphenol	1.380	5.672	↑	0.047
4.332	pos	1,2,3,4-Tetrahydro-beta-carboline	1.378	2.414	↑	0.026
5.419	neg	Nitisinone	1.374	3.123	↑	0.027
4.818	neg	BENZOYLARGININE NITROANILIDE	1.367	8.754	↑	0.029
4.818	neg	Phenylacetaldehyde	1.365	3.293	↑	0.039
6.718	neg	4-Butyl-5-hydroxy-1,2-diphenylpyrazol-3-one	1.350	2.144	↑	0.020
13.229	pos	N-(1-(1,2-Dihydro-1-methanesulfonylspiro(3H-indole-3,4'-	1.338	1.882	↑	0.041
11.330	pos	Epoxyganoderiol A	1.324	2.163	↑	0.024
13.075	pos	24-Nor-5beta-cholane-3alpha,12alpha,23-triol	1.321	2.417	↑	0.005
4.681	neg	DG(PGE2/0:0/2:0)	1.303	53.137	↑	0.033
8.882	neg	DG(10:0/0:0/18:1(12Z)-2OH(9,10))	1.293	7.067	↑	0.023
1.267	neg	(R)-Byakangelicin 3'-glucoside	1.292	17.783	↑	0.049
1.339	neg	5-Diazo-4-oxo-L-norvaline	1.285	2.562	↑	0.022

6.506	pos	Elephantopinolide A	1.280	4.527	↑	0.037
5.022	neg	OS-PI	1.278	2.503	↑	0.034
4.233	pos	3-[2-[[(1S,2R,3S)-3-[4-(Pentylcarbamoyl)-1,3-oxazol-2-yl]-7-	1.276	8.695	↑	0.019
5.701	neg	LEUCOGENENOL	1.270	3.487	↑	0.038
5.012	pos	Panaquinquecol 5	1.267	7.483	↑	0.024
4.363	neg	(5R)-5-Hydroxyhexanoic acid	1.264	2.845	↑	0.041
14.239	neg	3-hydroxyicosanoic Acid	1.260	2.208	↑	0.003
4.064	pos	2-(1-carboxy-2-hydroxypropyl)-4-{[5-	1.260	10.477	↑	0.024
12.716	neg	Harmalol	1.251	2.784	↑	0.033
1.375	neg	dTDP-4-acetamido-4,6-dideoxy-D-galactose	1.251	2.122	↑	0.042
5.844	neg	Oxindanac	1.248	6.839	↑	0.013
8.651	neg	1-tetradecanoyl-sn-glycero-3-phosphate	1.245	6.515	↑	0.048
11.541	pos	30:6(12Z,15Z,18Z,21Z,24Z,27Z)	1.243	2.179	↑	0.040
4.381	neg	26,26,26,27,27,27-hexafluoro-25-hydroxyvitamin D2	1.243	28.140	↑	0.039
4.663	neg	2-Hydroxyphenylacetic acid O-b-D-glucoside	1.240	5.861	↑	0.024
5.072	neg	Tyrosyl-Gamma-glutamate	1.239	2.664	↑	0.033
8.939	pos	Ouabain	1.229	2.931	↑	0.008
4.057	neg	Anti-phosphotyrosine	1.228	13.456	↑	0.021
4.782	pos	7,8-Dihydrovomifoliol 9-[rhamnosyl-(1->6)-glucoside]	1.226	6.226	↑	0.033
6.858	neg	LysoPI(20:0/0:0)	1.225	3.672	↑	0.041
8.291	neg	(2Z)-2-Morpholin-4-yliminoacetonitrile	1.205	1.944	↑	0.045
8.575	pos	PE(12:0/0:0)	1.190	2.583	↑	0.034
5.912	neg	Benzo(1,2-b:4,3-b')dipyrrole-2-carboxylic acid, 3,6,7,8-tetrahydro-	1.189	3.288	↑	0.044
4.381	neg	OHHdiA-PG	1.182	5.025	↑	0.039
7.499	neg	UNC-0638	1.176	8.961	↑	0.049
9.855	neg	PS(O-16:0/15:0)	1.168	16.272	↑	0.017
4.327	neg	(S)-6-Acetamido-2-(2-((S)-2-acetamido-4-	1.164	3.681	↑	0.042
5.854	pos	Fujikinetin methyl ether	1.162	2.921	↑	0.007
11.664	pos	LysoPA(O-18:0/0:0)	1.160	2.143	↑	0.027
4.834	neg	1-[(4R,4Ar,7S,7aR,12bS)-7,9-dihydroxy-3-methyl-1,2,4,4a,7a,13-	1.147	6.383	↑	0.034
5.560	neg	Gibberellin A97	1.146	3.484	↑	0.026
9.962	neg	13'-Carboxy-alpha-tocopherol	1.146	2.077	↑	0.033
5.613	neg	(-)-Warfarin	1.115	3.739	↑	0.040
1.231	neg	5,7-Dihydroxy-3,6,8,4'-tetramethoxyflavone 7-glucosyl-(1->3)-	1.110	41.124	↑	0.034
9.256	neg	ascr#26	1.109	1.663	↑	0.026
7.481	neg	Octyl propanoate	1.109	2.051	↑	0.049
1.411	neg	2-Hydroxy-L-methionine	1.105	2.315	↑	0.034
6.486	neg	Arctignan B	1.104	2.572	↑	0.035
0.769	neg	N-Gluconyl ethanolamine phosphate	1.101	2.012	↑	0.013
4.627	neg	Xanthinol	1.097	3.002	↑	0.041
3.812	pos	Bacampicillin	1.095	6.892	↑	0.033
4.591	neg	L-arginino-succinate	1.085	4.418	↑	0.021
10.241	pos	PG-PG	1.083	5.617	↑	0.025
2.385	neg	3alpha,4,7,7alpha-Tetrahydro-4-hydroxy-1H-isoindole-1,3(2H)-	1.080	1.851	↑	0.050

0.857	neg	Trigonelline	1.079	2.848	↑	0.026
5.419	neg	Indoleacetic acid	1.076	7.107	↑	0.020
8.344	neg	Gorgostane-1alpha,3beta,5alpha,6beta,11alpha,12beta-hexol 12-	1.075	10.386	↑	0.016
7.015	pos	(3S,4S,6R,7S)-1,10-Bisaboladiene-3,4-diol	1.073	1.332	↑	0.045
11.471	pos	Hellebrigenin-3-(12-hydroxy-laurate)	1.058	7.699	↑	0.020
2.774	pos	5-[(2R)-2-Aminopropyl]-2-hydroxybenzoic acid	1.056	2.680	↑	0.015
11.243	neg	LysoPS(18:1(9Z)/0:0)	1.055	2.682	↑	0.036
4.098	pos	Phenyl(piperidin-4-yl)methanone	1.053	10.206	↑	0.008
4.681	neg	Isopropyl 2-[[[(1R)-2-(6-aminopurin-9-yl)-1-methyl-	1.050	13.101	↑	0.034
6.038	neg	5-Hydroxyaloin A	1.045	2.492	↑	0.033
4.057	neg	2-(6'-methylthio)hexylmalate	1.036	5.472	↑	0.028
6.272	neg	4-Methyl-5-hexanolide	1.035	1.594	↑	0.041
5.155	pos	Serotonin O-sulfate	1.032	7.965	↑	0.047
7.966	neg	PGI2-EA	1.026	3.635	↑	0.026
3.846	pos	polypodine B	1.024	2.312	↑	0.013
14.277	pos	5alpha-Cholest-25-ene-3alpha,7alpha,12alpha-triol	1.009	11.239	↑	0.035
9.027	pos	PE(13:0/0:0)	1.005	6.198	↑	0.033
9.006	neg	C17 Sphingosine-1-phosphate	1.000	4.119	↑	0.008

Table S10 Significantly different metabolites in A1A2 cows and A2A2 cows

Retentio	Ion	Metabolites	VIP	FC	Regulation	*P*-value
9.322	pos	Pelargonidin 3-sophoroside	33.487	0.084	↓	0.001
10.664	neg	alpha-Dimorphecolic acid	15.395	0.342	↓	0.002
3.695	pos	4-Chloro-L-phenylalanine	14.612	0.586	↓	0.016
11.209	pos	Dibutyl phthalate	12.935	0.556	↓	0.008
10.241	pos	(10E,12Z)-(9S)-9-Hydroperoxyoctadeca-10,12-dienoic acid	12.552	0.371	↓	0.008
10.275	pos	9(S)-HOTrE	11.651	0.316	↓	0.002
8.211	pos	C16 Sphinganine	11.019	0.564	↓	0.016
9.291	neg	Pelargonidin 3,5-di-(6-acetylglucoside)	10.631	0.108	↓	0.002
6.612	neg	Apigenin	10.453	0.322	↓	0.007
8.183	neg	Calenduloside B	10.421	0.141	↓	0.004
7.179	neg	9S,10S,13R-trihydroxyoctadec-11E-enoic acid	10.331	0.579	↓	0.047
14.018	pos	pyridine-3,4-diol	9.562	0.483	↓	0.004
10.687	pos	Punicic acid	9.438	0.268	↓	0.001
5.701	neg	7,4'-Dihydroxyflavone	9.028	0.341	↓	0.005
6.625	pos	3-(3,4-Dihydroxyphenyl)-1-(2,3,4-trihydroxyphenyl)prop-2-	8.463	0.333	↓	0.007
10.949	pos	Ricinoleic acid	8.434	0.474	↓	0.001
4.559	pos	N1,N10-Dicoumaroylspermidine	8.419	0.139	↓	0.001
3.695	pos	Thiabendazole	8.325	0.588	↓	0.016
9.080	pos	Peonidin 3-rhamnoside 5-glucoside	7.649	0.044	↓	0.002
8.245	pos	9S,11R,15S-trihydroxy-2,3-dinor-13E-prostaenoic acid-	7.456	0.539	↓	0.044
9.829	pos	Prostaglandin H2	7.248	0.611	↓	0.045
8.768	pos	7-dehydrocholesterol-d7	7.228	0.638	↓	0.041

14.018	pos	(S)-Methyl 2,5-diamino-5-oxopentanoate	7.205	0.504	↓	0.005
9.847	pos	13(S)-Hydroperoxylinolenic acid	6.959	0.464	↓	0.020
6.042	pos	3-Deazaneplanocin A	6.732	0.060	↓	0.015
12.435	pos	MG(16:0/0:0/0:0)	6.659	0.367	↓	0.010
10.106	pos	N,N-dimethyl-Safingol	6.451	0.549	↓	0.031
0.754	pos	Adenine	6.369	0.450	↓	0.004
9.643	pos	17-Hydroxylinolenic acid	6.341	0.505	↓	0.025
10.928	neg	10-Oxo-11-octadecen-13-olide	6.172	0.597	↓	0.048
8.539	pos	Soyasaponin III	6.136	0.108	↓	<0.001
8.522	pos	Soyasaponin II	6.033	0.164	↓	0.006
12.652	neg	Ptilosteroid B	5.941	0.625	↓	0.015
0.635	neg	beta-D-fructose 2,6-bisphosphate	5.878	0.405	↓	0.008
12.643	pos	3-Palmitoyl-sn-glycerol	5.877	0.567	↓	0.013
9.847	pos	3-Oxotridecanoylcarnitine	5.440	0.164	↓	0.010
9.803	neg	9,10-DiHODE	5.315	0.547	↓	0.033
8.759	neg	Cholic acid	5.293	0.292	↓	0.008
13.517	neg	MG(i-18:0/0:0/0:0)	5.285	0.462	↓	0.030
8.784	pos	3alpha-Hydroxy-5beta-chola-7,9(11)-dien-24-oic Acid	5.264	0.282	↓	0.004
9.182	pos	Graminoxin A2	5.171	0.426	↓	0.006
11.071	pos	1-(11Z-docosenoyl)-glycero-3-phosphate	5.064	0.657	↓	0.021
8.759	neg	1alpha-hydroxy-24-(dimethylphosphoryl)-25,26,27-	4.956	0.604	↓	0.030
10.292	pos	(+)-15,16-Dihydroxyoctadecanoic acid	4.956	0.572	↓	0.006
0.661	pos	(E)-2-Penten-1-ol	4.933	0.535	↓	0.010
12.635	neg	TG(8:0/13:0/13:0)	4.854	0.597	↓	0.008
12.920	neg	4-Dodecylbenzenesulfonic Acid	4.738	0.594	↓	0.026
10.664	neg	15-Deoxyprostaglandin J2	4.670	0.432	↓	0.001
4.637	pos	Apigenin 7-[feruloyl-(->2)-glucuronyl-(1->2)-glucuronide] 4'-	4.629	0.055	↓	0.008
8.332	pos	16-epi-16-J1-PhytoP	4.617	0.523	↓	0.008
6.794	pos	5,7-Dihydroxy-8,4'-dimethoxyisoflavone	4.596	0.491	↓	0.046
6.385	pos	Medicagenic acid 3-O-b-D-glucuronide 28-O-[b-D-xylosyl-	4.488	0.163	↓	0.005
10.932	pos	3,4-Dimethyl-5-propyl-2-furannonanoic acid	4.395	0.553	↓	0.016
5.701	pos	Diferuloylputrescine	4.338	0.167	↓	0.009
9.729	pos	12,17;13,17-Diepoxy-16-hydroxy-9Z-octadecenoic acid	4.277	0.487	↓	0.003
9.097	pos	Cyanidin 3-(4'''-acetylrutinoside)	4.229	0.058	↓	0.001
5.125	pos	Apigenin 4'-(2''-feruloylglucuronosyl)-(1->2)-glucuronide	4.067	0.119	↓	0.030
10.106	pos	(9Z,11R,12S,13S,15Z)-12,13-Epoxy-11-hydroxy-9,15-	4.023	0.416	↓	0.014
11.902	neg	N-Undecylbenzenesulfonic acid	3.997	0.563	↓	0.027
10.687	pos	DG(15:0/0:0/18:4n3)	3.977	0.134	↓	0.011
5.887	pos	Wogonin	3.977	0.425	↓	0.011
11.209	pos	Phthalic acid	3.932	0.574	↓	0.009
6.629	neg	Pexacerfont	3.883	0.634	↓	0.050
4.818	neg	Lercanidipine	3.852	0.113	↓	0.003
12.748	pos	bhas#32	3.789	0.501	↓	0.006
9.305	pos	Simulanoquinoline	3.780	0.059	↓	0.001

13.229	pos	Pyridazine-3,6-diol	3.745	0.468	↓	0.003
3.795	pos	5S,6S-epoxy-15R-hydroxy-ETE	3.697	0.488	↓	0.019
9.363	neg	(9S,12S,16R)-d14-10-PhytoF[10S,13S]	3.592	0.193	↓	0.023
4.573	pos	Tricin 7-diglucuronoside	3.559	0.057	↓	<0.001
10.664	neg	DG(PGJ2/15:0/0:0)	3.520	0.190	↓	0.009
10.309	pos	(11S,12S,13S)-Epoxy-hydroxyoctadeca-cis-9-cis-15-dien-1-oic	3.514	0.413	↓	0.015
9.838	neg	(3R,4S,5S,6S)-6-[4-Chloro-2-(furan-2-ylmethylamino)-5-	3.503	0.654	↓	0.030
10.278	neg	9,10-Dihydroxystearic acid	3.447	0.676	↓	0.030
6.415	neg	Betavulgaroside X	3.446	0.251	↓	0.014
0.628	pos	Monoethylhexyl phthalic acid	3.405	0.451	↓	0.015
4.715	neg	Chrysoeriol 7-[feruloyl-(->2)-glucuronyl-(1->2)-glucuronyl-	3.396	0.041	↓	0.005
8.006	pos	Mabioside A	3.337	0.189	↓	0.007
10.949	pos	2-(5,8-Tetradecadienyl)cyclobutanone	3.327	0.465	↓	0.001
4.574	neg	Acacetin 7-glucuronosyl-(1->2)-glucuronide	3.269	0.057	↓	<0.001
12.364	pos	8,9-Epoxyeicosatrienoic acid	3.262	0.279	↓	0.043
8.487	pos	16-B1-phytoprostane	3.244	0.433	↓	0.015
10.946	neg	9-HETE	3.236	0.602	↓	0.005
2.924	pos	(1S,4R)-1-Hydroxy-2-oxolimonene	3.230	0.597	↓	0.038
5.701	pos	Rheidin B	3.224	0.238	↓	0.008
1.366	pos	L-Isoleucine	3.219	0.633	↓	0.040
8.002	neg	1-[[Difluoro-(2,3,4-trifluorophenyl)methoxy]-difluoromethyl]-	3.175	0.647	↓	0.023
0.693	pos	Creatinine	3.154	0.199	↓	0.017
7.134	pos	N-(9-Oxodecyl)acetamide	3.133	0.464	↓	0.007
5.161	neg	Camellioside A	3.106	0.051	↓	<0.001
6.437	pos	Quillaic acid 3-[xylosyl-(1->3)-[galactosyl-(1->2)]-	3.091	0.203	↓	0.005
4.970	neg	Genistein 7-O-glucuronide	3.079	0.066	↓	0.001
5.580	pos	Quinaprilat	3.053	0.128	↓	0.003
5.234	pos	Kudzusaponin SA4	2.997	0.062	↓	<0.001
7.203	pos	Neocnidolide	2.982	0.573	↓	0.013
9.760	pos	PGP(LTE4/i-12:0)	2.961	0.108	↓	0.004
4.979	pos	Tricin 7-(2''-p-coumaroylglucuronosyl)(1->2)glucronide	2.957	0.055	↓	0.021
8.922	pos	Pisumsaponin II	2.957	0.081	↓	0.003
7.489	pos	Glycocholic acid	2.954	0.086	↓	0.005
8.146	neg	28-[Glucosyl-(1->6)-glucosyl]oleanolic acid 3-arabinoside	2.926	0.116	↓	0.005
5.172	pos	Camellidin II	2.916	0.034	↓	0.001
4.557	neg	Luteolin 3'-methyl ether 7-glucuronosyl-(1->2)-glucuronide	2.854	0.057	↓	<0.001
4.979	pos	Tricin 7-[feruloyl-(->2)-glucuronyl-(1->2)-glucuronide]	2.839	0.051	↓	0.028
4.669	pos	Apigenin 7-glucuronide-4'-(2'''-E-p-coumaroylglucuronosyl)-	2.818	0.057	↓	0.011
8.759	neg	Hordatine A	2.812	0.580	↓	0.031
8.280	pos	5Z-Tetradecen-1-ol	2.810	0.625	↓	0.024
4.493	pos	Icariside B8	2.808	0.194	↓	0.004
13.655	pos	3-Amino-2-methoxynonadec-5-en-4-ol	2.804	0.353	↓	0.018
5.615	pos	Apoanagallosaponin IV	2.786	0.035	↓	0.001
14.018	pos	trans-2-Phenylcyclopropylamine	2.750	0.552	↓	0.008

9.406	pos	Momordicoside L	2.738	0.203	↓	0.001
10.793	pos	Naphtho(1,2-c)furan-3(1H)-one, 4,5,5a,6,7,8,9,9a-octahydro-	2.714	0.562	↓	0.003
9.526	pos	Nandrolone	2.706	0.504	↓	0.013
7.219	pos	Quillaic acid 3-[galactosyl-(1->2)-glucuronide]	2.654	0.043	↓	0.001
4.970	neg	Chrysoeriol 7-(2'''-feruloylglucuronosyl)-(1->2)-glucuronide	2.633	0.062	↓	0.014
10.227	neg	(3-{[3-(2-Amino-2-Oxoethyl)-1-Benzyl-2-Ethyl-1h-Indol-5-	2.624	0.296	↓	0.044
8.075	pos	LPIM2(16:0/0:0)	2.620	0.012	↓	0.005
8.159	pos	Cucurbitacin I 2-glucoside	2.606	0.043	↓	<0.001
5.464	pos	Crocin 5	2.595	0.235	↓	0.004
6.576	pos	Madecassoside	2.593	0.147	↓	0.006
4.485	neg	Apigenin 7-O-diglucuronide	2.584	0.057	↓	<0.001
8.627	pos	Ophioxanthin	2.576	0.038	↓	0.006
13.536	pos	MG(18:0/0:0/0:0)	2.569	0.360	↓	0.008
7.971	pos	3beta,12alpha-Dihydroxy-5beta-cholestan-26-oic acid	2.557	0.111	↓	0.005
8.057	pos	6alpha-Carissanol	2.554	0.530	↓	<0.001
9.847	pos	9S-HpOTrE	2.546	0.474	↓	0.019
5.187	pos	(4-Aminophenyl)phosphonic acid	2.541	0.565	↓	0.013
9.305	pos	16-Hydroxy-10-oxohexadecanoic acid	2.539	0.586	↓	0.042
4.717	pos	Tricin 7-[feruloyl-(->2)-glucuronyl-(1->2)-[glucuronyl-(1->3)]-	2.526	0.041	↓	0.003
6.790	neg	Viscutin 1	2.521	0.340	↓	0.041
5.005	neg	Irtemazole	2.520	0.318	↓	0.004
11.332	neg	2-Nitrophenyl octyl ether	2.511	0.590	↓	0.022
12.500	neg	Arachidonic acid	2.504	0.230	↓	0.013
10.664	neg	Macrophyllol A (flavone)	2.470	0.343	↓	0.002
10.788	neg	16-Hydroxyhexadecanoic acid	2.437	0.606	↓	0.004
5.701	pos	Soyasaponin V	2.426	0.032	↓	0.001
4.620	pos	Phenylalanyl-prolyl-arginine-chloromethyl ketone	2.418	0.142	↓	0.006
6.290	neg	28-Glucosylarjunolate 3-[rhamnosyl-(1->3)-glucuronide]	2.384	0.058	↓	0.001
5.413	pos	Kudzusaponin SA2	2.377	0.110	↓	<0.001
12.922	pos	1-O-(2R-hydroxy-nonadecyl)-sn-glycerol	2.371	0.715	↓	0.050
8.159	pos	3beta-3-Hydroxy-18-lupen-21-one	2.365	0.103	↓	0.002
6.093	pos	6'-Hydroxybuspirone	2.357	0.286	↓	0.024
3.897	pos	Propacetamol	2.357	0.453	↓	0.046
6.420	pos	Medicagenic acid beta-maltoside	2.352	0.082	↓	<0.001
4.397	pos	SM(d19:1/24:1(15Z))	2.349	0.007	↓	0.008
5.596	neg	Medinoside E	2.330	0.041	↓	0.002
8.751	pos	Soyasaponin IV	2.328	0.217	↓	0.001
8.245	pos	Plasmodiophorol C	2.328	0.596	↓	0.038
12.643	pos	Dihydroouabain	2.322	0.297	↓	0.001
0.752	neg	FAPy-adenine	2.304	0.395	↓	<0.001
5.529	pos	Astemizole	2.263	0.399	↓	0.014
6.042	pos	Centellasaponin C	2.259	0.160	↓	0.014
7.660	pos	Tridec-11-enedioylcarnitine	2.258	0.229	↓	0.012
8.917	neg	Dehydrosoyasaponin I	2.245	0.078	↓	0.003

13.088	neg	Adrenic acid	2.238	0.553	↓	0.045
1.460	pos	Homomethionine	2.232	0.228	↓	0.010
10.788	neg	Doisynoestrol	2.230	0.572	↓	0.040
10.664	neg	Dimethylcurcumin	2.229	0.222	↓	0.015
4.831	pos	Soyasaponin A2	2.207	0.091	↓	0.004
5.243	neg	Luteolin 5,3'-dimethyl ether 7-glucoside	2.206	0.054	↓	0.018
14.259	pos	1-Methylhistidine	2.197	0.475	↓	0.004
4.282	pos	Octaethylene glycol	2.193	0.650	↓	0.028
8.775	neg	15-keto Latanoprost	2.184	0.303	↓	0.007
8.506	neg	Somatotropin (6-13)	2.160	0.181	↓	0.010
9.803	neg	Resolvin E2	2.159	0.543	↓	0.021
7.109	neg	Calcium ascorbate	2.156	0.674	↓	0.018
6.718	neg	Trillfurostanoside F	2.148	0.025	↓	0.007
8.615	neg	Elaterinide	2.144	0.064	↓	0.010
7.186	pos	PA(22:6(4Z,7Z,10Z,13Z,16Z,19Z)/15:1(9Z))	2.142	0.322	↓	0.029
5.125	pos	NADH pyruvate	2.132	0.085	↓	0.042
9.873	neg	UDP-alpha-D-xylose	2.126	0.653	↓	0.033
9.044	pos	5'-Carboxy-gamma-chromanol	2.120	0.506	↓	0.030
4.003	neg	Sibiricose A5; 3-O-[(2E)-3-(4-Hydroxy-3-methoxyphenyl)-2-	2.115	0.206	↓	0.034
5.984	neg	Asa-PS	2.112	0.009	↓	0.007
10.983	pos	abietadiene-diol	2.100	0.453	↓	0.005
1.036	pos	(9R,13R)-1a,1b-dihomo-jasmonic acid	2.086	0.603	↓	0.019
10.488	neg	13-keto myristic acid	2.085	0.344	↓	<0.001
9.812	pos	8-iso-PGA1	2.081	0.704	↓	0.008
10.343	pos	Arachidoyl Ethanolamide	2.081	0.576	↓	0.022
4.963	pos	5-Megastigmen-7-yne-3,9-diol 3-glucoside	2.077	0.254	↓	0.004
10.670	pos	LysoPE(18:2(9Z,12Z)/0:0)	2.068	0.355	↓	0.019
3.711	pos	Pentaethylene glycol	2.067	0.610	↓	0.021
8.092	pos	7-Hydroxytrideca-9,11-dienoylcarnitine	2.055	0.338	↓	0.008
8.469	pos	Trillfurostanoside E	2.049	0.051	↓	0.017
9.235	pos	N-Myristoyl Glutamic acid	2.046	0.132	↓	0.012
9.680	neg	N-methyl-4,6,7-trihydroxy-1,2,3,4-tetrahydroisoquinoline	2.045	0.586	↓	0.022
1.271	pos	3,7,12-Trioxochola-1,4-dien-24-oic Acid	2.024	0.715	↓	0.015
1.036	pos	3-Oxoglutaric acid	2.024	0.585	↓	0.015
5.560	neg	O-Ethyl S,S-diphenyl phosphorodithioate	2.022	0.651	↓	0.018
5.615	pos	PG(18:3(9Z,12Z,15Z)/0:0)	2.016	0.202	↓	0.002
9.908	neg	Ethyl gallate 3-sulfate	2.012	0.664	↓	0.042
10.928	neg	Anguvetin	2.003	0.458	↓	0.001
10.326	pos	Dodecanamide	2.001	0.562	↓	0.040
1.067	pos	L-Methionine	1.989	0.401	↓	0.002
11.226	pos	Monoisobutyl phthalic acid	1.989	0.579	↓	0.010
10.687	pos	5-Oxo-6,8,11,14-eicosatetraenoic acid	1.988	0.326	↓	0.001
9.661	pos	12-Ketodeoxycholic acid	1.983	0.538	↓	0.020
3.880	pos	DGMG(18:5(3Z,6Z,9Z,12Z,15Z)/0:0)	1.972	0.073	↓	0.004

5.684	neg	Trillfurostanoside B	1.969	0.164	↓	0.009
4.557	neg	6S,9R-Dihydroxy-4,7E-megastigmadien-3-one 9-[apiosyl-	1.968	0.131	↓	0.001
13.417	pos	L-2,4-diaminobutyric acid	1.955	0.493	↓	0.006
9.560	pos	Dehydroisoandrosterone 3-glucuronide	1.953	0.160	↓	0.016
5.498	pos	Foretinib	1.951	0.338	↓	0.019
8.453	pos	Gallicynoic acid A	1.935	0.583	↓	0.005
10.928	neg	4-Hydroxyretinoic acid	1.929	0.619	↓	0.044
4.988	neg	6''-O-(3-Hydroxy-3-methylglutaroyl)astragalin	1.929	0.008	↓	0.008
10.226	pos	3b-Hydroxy-5-cholenoic acid	1.926	0.544	↓	0.044
10.498	pos	14-hydroxy-5Z-tetradecenoic acid	1.925	0.232	↓	0.004
4.183	pos	CAY10589	1.922	0.372	↓	0.026
4.620	pos	Angelicoidenol 2-O-beta-D-glucopyranoside	1.921	0.408	↓	0.005
4.238	neg	Sialorphin	1.915	0.142	↓	0.002
11.558	pos	Chenodeoxycholylasparagine	1.912	0.550	↓	0.029
11.053	pos	MG(0:0/20:4(5Z,8Z,11Z,13E)-OH(15S)/0:0)	1.908	0.764	↓	0.049
5.430	pos	5-Methoxyindoleacetate	1.904	0.149	↓	0.025
11.769	pos	5S-HETE di-endoperoxide	1.901	0.598	↓	0.017
6.397	neg	Medicoside G	1.900	0.093	↓	0.001
7.287	pos	Indole-3-methyl acetate	1.893	0.074	↓	0.010
13.194	pos	icos#9	1.891	0.628	↓	0.008
10.227	neg	8-Oxodecanoylcarnitine	1.884	0.322	↓	0.040
4.460	pos	Apigenin 7-[glucuronyl-(1->2)-glucuronide] 4'-glucuronide	1.872	0.034	↓	0.001
11.961	pos	1-O-(2R-hydroxy-pentadecyl)-sn-glycerol	1.870	0.496	↓	0.005
8.723	neg	Pitheduloside A	1.869	0.238	↓	0.001
5.948	neg	N-Nitrosothiazolidine-4-carboxylic acid	1.866	0.673	↓	0.018
9.327	neg	Pyranocyanin A	1.864	0.059	↓	0.002
3.533	neg	Leonuriside A	1.851	0.036	↓	0.023
6.385	pos	Ampeloside Bs1	1.844	0.151	↓	0.006
5.419	neg	Hovenoside I	1.840	0.129	↓	<0.001
4.416	neg	Sordarin	1.832	0.139	↓	0.003
4.880	pos	Tricin 7-[rhamnosyl-(1->2)-galacturonide]	1.827	0.073	↓	0.001
5.598	pos	FP-biotin	1.821	0.176	↓	<0.001
8.759	neg	N-Docosahexaenoyl phenylalanine	1.820	0.646	↓	0.043
9.716	neg	9-epiSacrolide A	1.818	0.462	↓	0.014
7.608	pos	Isoformononetin	1.816	0.121	↓	0.001
12.433	neg	1,4-Dimethyl-7-ethylazulene	1.811	0.401	↓	0.028
7.055	neg	Quillaic acid 3-[rhamnosyl-(1->3)-[galactosyl-(1->2)]-	1.806	0.032	↓	0.002
8.470	neg	Pisumsaponin I	1.801	0.048	↓	0.013
11.000	pos	Alpha-Linolenoyl ethanolamide	1.792	0.628	↓	0.002
7.851	pos	8-O-Methylretusin	1.776	0.123	↓	0.001
8.505	pos	7,11-Bisdeacetylvaltrate 7-(3-methylpentanoate) 11-(3-	1.756	0.120	↓	0.009
4.132	pos	Heptaethylene glycol	1.753	0.705	↓	0.031
10.261	neg	Armillatin	1.749	0.228	↓	0.018
10.227	neg	Palomid 529	1.747	0.475	↓	0.022

4.818	neg	urea citrate	1.741	0.615	↓	0.026
7.821	neg	Pentadecanoylglycine	1.739	0.158	↓	0.005
12.022	neg	2-Hydroxyhexadecanoic acid	1.738	0.546	↓	0.045
8.723	neg	Cimicifugoside	1.729	0.021	↓	0.005
7.851	pos	Corchorifatty acid F	1.728	0.642	↓	0.046
12.211	neg	MG(20:4(8Z,11Z,14Z,17Z)/0:0/0:0)	1.724	0.599	↓	0.021
9.948	pos	Ambrettolic acid	1.723	0.469	↓	0.020
5.580	pos	Cucurbitacin F	1.718	0.214	↓	0.008
5.878	neg	Pachybasin	1.715	0.361	↓	0.007
5.802	pos	PG(22:4(7Z,10Z,13Z,16Z)/0:0)	1.713	0.026	↓	0.042
10.793	pos	cis-Palmitvaccenic acid	1.709	0.531	↓	0.001
5.904	pos	26-(2-Glucosyl-6-acetylglucosyl]-1,3,11,22-	1.707	0.068	↓	0.001
7.099	pos	Tenuifolin	1.706	0.328	↓	0.020
1.589	pos	TRIETHYLENE GLYCOL	1.693	0.544	↓	0.037
8.768	pos	PS(22:1(13Z)/TXB2)	1.688	0.544	↓	0.012
5.091	neg	9-Hydroxy-7-megastigmen-3-one glucoside	1.684	0.238	↓	0.006
13.672	pos	DG(22:5(4Z,7Z,10Z,13Z,16Z)/24:1(15Z)/0:0)	1.669	0.524	↓	0.019
10.967	pos	12-HETE	1.669	0.437	↓	0.002
10.946	neg	DG(a-15:0/20:4(5Z,7E,11Z,14Z)-OH(9)/0:0)	1.666	0.377	↓	0.008
6.807	neg	Ginseng	1.666	0.254	↓	0.012
11.209	pos	8-Hydroxymianserin	1.656	0.561	↓	0.008
4.801	neg	Beta-D-Fructose 6-phosphate	1.639	0.640	↓	0.020
4.093	neg	Vignatic acid A	1.635	0.097	↓	0.019
10.241	pos	9,10,11-trihydroxy-12Z-octadecenoic acid	1.630	0.681	↓	0.009
4.627	neg	Lirodenine	1.629	0.057	↓	0.001
13.008	pos	Perflutren	1.622	0.486	↓	0.007
13.417	pos	Carcinine	1.620	0.502	↓	0.005
4.637	pos	2-Phenylethyl beta-D-glucopyranoside	1.614	0.396	↓	0.005
13.606	pos	3-Methyl-3-heptene	1.613	0.649	↓	0.038
6.074	neg	(6E,10E)-3-Hydroxydodeca-6,10-dienoylcarnitine	1.612	0.265	↓	0.025
0.723	pos	D-Proline	1.611	0.534	↓	0.019
7.186	pos	Lipoxin B4	1.610	0.653	↓	0.013
9.729	pos	13-HODE-EA	1.608	0.312	↓	0.001
4.184	neg	4-O-(Indole-3-acetyl)-D-glucopyranose	1.608	0.350	↓	0.029
8.759	neg	LysoPC(18:4(6Z,9Z,12Z,15Z)/0:0)	1.606	0.626	↓	0.041
5.331	neg	Araliasaponin III	1.604	0.045	↓	0.002
12.652	neg	11-(Dansylamino)undecanoic acid	1.592	0.629	↓	0.012
6.254	neg	17alpha-(N-acetyl-alpha-D-glucosaminyl)estradiol 3-	1.588	0.222	↓	0.010
6.197	pos	N-Decanoylglycine	1.580	0.693	↓	0.041
4.542	pos	6,8-Di-C-beta-D-arabinopyranosylapigenin	1.580	0.292	↓	0.017
10.275	pos	Telocinobufagin-3-(14-hydroxymyristate)	1.578	0.256	↓	0.019
4.559	pos	N-decanoyl histidine	1.577	0.130	↓	0.001
7.015	pos	Ascaroside C3	1.564	0.423	↓	0.045
4.219	neg	Cornoside	1.564	0.208	↓	0.005

5.785	pos	Cyclo(Leu-Phe)	1.560	0.369	↓	0.016
10.241	pos	DG(19:0/0:0/20:5(7Z,9Z,11E,13E,17Z)-3OH(5,6,15))	1.554	0.325	↓	0.024
10.261	neg	DG(PGE2/15:0/0:0)	1.554	0.395	↓	0.026
8.400	pos	1-(2,6,6-Trimethyl-2-cyclohexen-1-yl)-1,6-heptadien-3-one	1.551	0.498	↓	0.027
10.687	pos	Cyclopassifloside VI	1.535	0.160	↓	0.005
6.522	neg	Taurocholic acid	1.534	0.174	↓	0.010
12.643	pos	20-Hydroxy-leukotriene E4	1.531	0.478	↓	0.003
4.332	pos	Cinnamoside	1.531	0.257	↓	0.034
4.274	neg	Aromadendrin 3-galactoside	1.525	0.291	↓	0.040
8.799	pos	Methymycin	1.523	0.473	↓	<0.001
4.274	neg	N-(1-Deoxy-1-fructosyl)histidine	1.519	0.174	↓	0.002
11.347	pos	(-)-Malyngolide	1.517	0.469	↓	<0.001
1.215	neg	6-Formylindolo [3,2-B] carbazole	1.517	0.606	↓	0.026
8.056	neg	3-[(1R,2R,3S)-3-Carboxy-1,2,3-trihydroxypropyl]dioxirane-3-	1.510	0.677	↓	0.017
10.687	pos	[7]-Paradol	1.506	0.518	↓	0.007
0.768	pos	2-Phenylethyl octanoate	1.506	0.469	↓	0.004
5.471	neg	Propericiazine	1.500	0.126	↓	0.012
4.963	pos	5a,6a-Epoxy-7E-megastigmene-3a,9e-diol 3-glucoside	1.499	0.323	↓	0.010
9.677	pos	Berkeleylactone L	1.498	0.539	↓	0.002
3.966	neg	Lamioside	1.493	0.117	↓	0.008
4.574	neg	Myricetin 3-O-(4''-O-acetyl-2''-O-galloyl)-alpha-L-	1.492	0.052	↓	<0.001
3.894	neg	Dihydroferulic acid 4-O-glucuronide	1.477	0.168	↓	0.006
10.687	pos	Piroxantrone	1.473	0.326	↓	0.001
2.774	pos	1-Hexadecanol	1.468	0.606	↓	0.015
10.770	neg	Irene	1.468	0.624	↓	0.009
5.012	pos	PGP(18:0/22:4(7Z,10Z,13Z,16Z))	1.465	0.078	↓	0.003
4.539	neg	Citrusin B	1.459	0.163	↓	0.008
7.473	pos	Sphingofungin B	1.456	0.287	↓	0.002
7.179	neg	(Z)-7-[(1S,4R,6R)-4-[(E)-Oct-6-enyl]-2,3-	1.456	0.571	↓	0.037
8.759	neg	DGDG(16:0/18:3(9Z,12Z,15Z))	1.456	0.610	↓	0.050
13.314	pos	2-Imino-4-methylpiperidine	1.451	0.658	↓	0.022
1.055	neg	1H-Pyrazolo[3,4-d]pyrimidin-4-amine	1.451	0.475	↓	0.001
0.798	pos	Shanzhiside methyl ester	1.450	0.147	↓	0.028
10.296	neg	5,10-Pentadecadien-1-ol	1.447	0.410	↓	0.002
0.754	pos	5-((6-((Aminomethyl)amino)-1-oxohexyl)amino)pentanoic	1.446	0.149	↓	0.004
7.036	neg	Nocardicin A	1.441	0.025	↓	0.021
5.895	neg	Sufotidine	1.435	0.082	↓	0.001
8.522	pos	phorbol 13-acetate 12-myristate	1.431	0.137	↓	0.002
10.664	neg	PG(22:1(11Z)/22:1(11Z))	1.430	0.123	↓	0.020
12.643	pos	20-Oxo-leukotriene E4	1.419	0.465	↓	0.006
7.499	neg	Medicagenic acid 28-O-[b-D-xylosyl-(1->4)-a-L-rhamnosyl-	1.418	0.151	↓	0.004
13.332	pos	Diphenylamine	1.411	0.436	↓	0.002
10.932	pos	Cucumariaxanthin C	1.411	0.291	↓	0.007
9.733	neg	(±)-(E)-3-Methyl-4-decen-1-ol	1.409	0.494	↓	0.005

8.989	neg	3-Hydroxy-4-aminopyridine sulfate	1.405	0.617	↓	0.019
5.820	pos	PGP(i-15:0/LTE4)	1.405	0.040	↓	0.001
4.292	neg	Nifekalant	1.405	0.281	↓	0.021
3.679	pos	Gentamicin A sulfate	1.400	0.128	↓	0.011
9.504	neg	Methylpyrogallol sulfate 3	1.400	0.626	↓	0.029
5.060	pos	Kaempferol 3-methyl ether 7-glucuronide	1.397	0.015	↓	0.040
8.775	neg	Cimimanol F	1.395	<0.00	↓	0.008
9.574	neg	Glc-GP(18:0/0:0)	1.395	0.050	↓	0.001
4.637	pos	Daidzein 4'-O-glucuronide	1.387	0.053	↓	0.012
13.059	pos	Stearoylethanolamide	1.382	0.563	↓	0.019
5.546	pos	N-(p-Hydroxyphenyl)ethyl p-hydroxycinnamide	1.377	0.279	↓	0.001
4.979	pos	ascr#12	1.376	0.310	↓	0.047
11.523	pos	1-O-(2R-hydroxy-tetradecyl)-sn-glycerol	1.375	0.585	↓	0.024
14.071	pos	N-Docosahexaenoyl Tryptophan	1.373	0.389	↓	0.019
8.159	pos	Goyaglycoside f	1.369	0.094	↓	0.003
2.924	pos	2,6-diamino-4-hydroxy-5-formamidopyrimidine	1.364	0.612	↓	0.034
12.973	pos	14,15-EE-8(Z)-E	1.364	0.428	↓	0.002
12.817	pos	13-Heptadecyn-1-ol	1.358	0.597	↓	0.017
4.450	neg	Hexafluoropropene	1.357	0.567	↓	0.021
11.000	pos	7a-Hydroxy-5b-cholanic acid	1.352	0.615	↓	0.011
5.264	pos	Oxyayanin B 3'-glucoside	1.352	0.064	↓	0.007
10.532	pos	(R)-4-(2-(2-(2-Methylpyrrolidin-1-yl)ethyl)benzofuran-5-	1.347	0.500	↓	0.002
10.138	neg	1-[2,3-Dimethyl-2-(2-methylbut-3-en-2-yl)furan-3-yl]-3,5-	1.337	0.590	↓	0.009
4.299	pos	Sulfamide, N,N-dimethyl-N'-((8alpha)-6-propylergolin-8-yl)-	1.328	0.160	↓	0.002
12.040	neg	Isovalerylsarcosine	1.324	0.512	↓	0.008
10.227	neg	PI(22:0/19:1(9Z))	1.324	0.110	↓	0.030
9.475	pos	7Z-hexadecen-1-ol	1.323	0.620	↓	0.028
0.709	pos	1,2-Ethanedisulfonic acid	1.323	0.577	↓	0.020
10.946	neg	OSU03012	1.322	0.491	↓	0.002
0.821	neg	Trimetaphosphoric acid	1.320	0.638	↓	0.038
1.382	pos	Zalcitabine	1.310	0.298	↓	0.012
4.098	pos	Mauritine A	1.308	0.027	↓	0.004
9.913	pos	Butylparaben	1.306	0.566	↓	0.009
12.049	pos	6-Hydroxy-8-methyl-8-azabicyclo[3.2.1]octan-3-yl 3-hydroxy-	1.299	0.474	↓	0.010
9.220	neg	N-tetradecanoyl-homoserine lactone	1.299	0.133	↓	0.011
12.834	pos	(2R,3Z)-Phycocyanobilin	1.297	0.348	↓	0.002
6.180	pos	3b-Pregnadienolone 3-[rhamnosyl-(1->4)-rhamnosyl-(1->4)-	1.296	0.051	↓	0.003
4.884	neg	Glucodistylin	1.295	0.131	↓	0.032
9.645	neg	Piridronic acid	1.293	0.655	↓	0.025
8.759	neg	Camicinal	1.293	0.608	↓	0.029
10.928	neg	Candidone	1.291	0.485	↓	0.008
10.775	pos	11-Deoxy-PGE1	1.283	0.514	↓	0.016
8.092	pos	Digitoxigenin bisdigitoxide	1.280	0.028	↓	<0.001
6.594	neg	Coumarin-3-carboxylic acid succinimidyl ester	1.280	0.382	↓	0.004

12.040	neg	4-(Methylnitrosamino)-1-(3-pyridyl)-1-butanone	1.279	0.513	↓	0.005
5.808	neg	1alpha,3beta,22R-Trihydroxyergosta-5,24E-dien-26-oic acid 3-	1.278	0.049	↓	0.002
12.660	pos	7beta-Hydroxy-12-oxo-5alpha-cholan-24-oic Acid	1.278	0.487	↓	<0.001
12.678	pos	Dihomolinoleic acid	1.276	0.547	↓	0.001
6.700	neg	(2S,5R)-3,3-Dimethyl-2-(2-phenylethyl)-4-thia-1-	1.275	0.068	↓	0.005
12.643	pos	bhas#22	1.275	0.514	↓	0.009
10.191	neg	UDP-D-galacturonic acid	1.273	0.672	↓	0.045
10.687	pos	Bapta	1.273	0.298	↓	0.002
4.995	pos	Cer(d18:1(4E)/32:0(2OH))	1.265	0.143	↓	0.038
5.802	pos	Acuminoside	1.264	0.111	↓	0.007
10.635	pos	Met-leu-phe	1.262	0.197	↓	0.001
9.981	pos	Prostaglandin B-2	1.262	0.510	↓	0.031
9.166	pos	Deoxycholylglycine	1.257	0.067	↓	0.003
8.768	pos	Emetine	1.256	0.556	↓	0.024
9.539	neg	Capsianoside V	1.252	0.110	↓	0.021
4.669	pos	Isolariciresinol 9-O-beta-D-glucoside	1.249	0.183	↓	0.029
6.665	neg	Notoginsenoside R2	1.249	0.031	↓	0.002
6.316	pos	Lyciumoside VII	1.246	0.042	↓	0.001
7.162	neg	5-Fluorouridine monophosphate	1.244	0.690	↓	0.018
14.313	pos	Secnidazole	1.238	0.542	↓	0.008
9.677	pos	17(S)-HETE	1.237	0.660	↓	0.016
10.879	pos	8,8-Diethoxy-2,6-dimethyl-2-octanol	1.234	0.483	↓	0.010
10.928	neg	[(2S,3As,7aS)-Octahydro-1-[[(1R,2R)-2-	1.234	0.601	↓	0.038
7.186	pos	n-Formyl-methionyl-leucyl-phenyl-alanine	1.227	0.423	↓	0.002
9.096	neg	Pelargonidin 3-(6''-malonylglucoside)-5-(6'''-acetylglucoside)	1.227	0.025	↓	0.003
7.587	neg	Dihydrowogonin	1.226	0.134	↓	0.001
11.332	neg	9S-hydroxy-7E-hexadecenoic acid	1.223	0.569	↓	0.003
4.814	pos	Vanillin	1.216	0.380	↓	0.001
8.159	pos	Neuromedin N	1.215	0.102	↓	0.002
10.326	pos	LysoPE(0:0/20:4(8Z,11Z,14Z,17Z))	1.211	0.147	↓	0.006
12.108	neg	Cetylmannoside	1.210	0.668	↓	0.046
7.539	pos	(S)-10,16-Dihydroxyhexadecanoic acid	1.207	0.699	↓	0.046
5.666	pos	epsilon-(Hexanoyl)lysine	1.207	0.345	↓	0.021
13.690	pos	PA(15:0/24:0)	1.204	0.523	↓	0.033
3.678	neg	S-(2-Furanylmethyl) methanethioate	1.203	0.529	↓	0.022
9.829	pos	Apo-13-zeaxanthinone	1.203	0.494	↓	0.018
6.290	neg	Betavulgaroside VI	1.202	0.041	↓	0.002
12.523	pos	12R-hydroxy-5Z,8Z,12Z-eicosatrienoic acid	1.201	0.209	↓	0.007
0.677	pos	Disodium phosphate	1.199	0.471	↓	0.032
4.315	pos	Benzyl alcohol beta-D-rutinoside	1.198	0.259	↓	0.009
8.165	neg	Abrusoside B	1.194	0.015	↓	0.007
0.739	pos	Icaridin	1.191	0.268	↓	0.034
5.413	pos	2-(3-Carboxy-3-aminopropyl)-L-histidine	1.191	0.129	↓	0.005
6.676	pos	1-Acetyl-3,14,20-trihydroxywitha-5,24-dienolide 3-glucoside	1.189	0.010	↓	0.001

12.433	neg	N-Oleoyl Asparagine	1.188	0.438	↓	0.021
7.270	pos	Nb-Feruloyltryptamine	1.185	0.119	↓	0.004
8.775	neg	Perindopril Acyl-beta-D-glucuronide	1.185	0.253	↓	0.011
10.932	pos	Methysergide	1.180	0.483	↓	0.014
10.393	pos	Athanacalvic acid	1.179	0.560	↓	0.008
9.557	neg	19-hydroxytelocinobufagin	1.178	0.243	↓	0.030
14.313	pos	Alanylhydroxyproline	1.171	0.506	↓	0.004
5.295	neg	Perfluoroisobutylene	1.167	0.598	↓	0.023
8.991	pos	Diethyl phthalic acid	1.162	0.617	↓	0.017
7.018	neg	Elatoside G	1.160	0.348	↓	0.014
10.258	pos	6,9,12,15-octadecatetraenoic acid	1.157	0.358	↓	0.002
4.539	neg	Sulfamethoxazole N1-glucuronide	1.156	0.083	↓	0.006
8.002	neg	Dimethyl 2,3-bis(sulfanyl)butanedioate	1.152	0.660	↓	0.022
10.652	pos	2-[3]-ladderane ethanoic acid	1.152	0.228	↓	0.001
4.588	pos	4'-Hydroxy-5,7,2'-trimethoxyflavanone 4'-rhamnosyl-(1->6)-	1.148	0.196	↓	0.021
7.660	pos	gitoxin	1.148	0.168	↓	0.004
9.363	neg	Diflucortolone	1.147	0.287	↓	0.025
10.541	neg	Tryptophyl-Arginine	1.144	0.545	↓	0.026
4.782	pos	Diacetylfusarochromanone	1.142	0.438	↓	0.027
8.784	pos	Palmitoyl Ara-C	1.141	0.498	↓	0.017
11.035	pos	Tris(2-butoxyethyl) phosphate	1.140	0.634	↓	0.020
4.559	pos	Enol-phenylpyruvate	1.139	0.255	↓	0.001
10.967	pos	Valtratum	1.138	0.401	↓	0.003
7.746	pos	PGP(18:1(9Z)/LTE4)	1.134	0.071	↓	0.006
11.000	pos	21-oxo-docosanoic acid	1.128	0.682	↓	0.038
14.331	pos	Ectoine	1.125	0.502	↓	0.004
6.007	pos	LPIM3(16:0/0:0)	1.124	0.004	↓	0.013
12.652	neg	Tert-Butyl (4-(3-((7-(hydroxyamino)-7-	1.121	0.639	↓	0.013
7.875	neg	11-Dihydro-12-norneoquassin	1.121	0.624	↓	0.012
6.629	neg	2-Phenylpyrazolo(4,3-c)quinolin-3(5H)-one	1.118	0.412	↓	0.028
8.505	pos	1,3-Dicyclohexylurea	1.115	0.585	↓	0.012
3.965	pos	(1R,2R,4S)-p-Menthane-1,2,8-triol 8-glucoside	1.114	0.324	↓	0.050
3.678	neg	Iguratimod	1.114	0.484	↓	0.033
13.229	pos	2',3'-Didehydro-2',3'-dideoxycytidine	1.110	0.587	↓	0.004
7.270	pos	Swertisin	1.109	0.551	↓	0.002
11.209	pos	PKODiA-PA	1.109	0.406	↓	0.007
5.055	neg	Benzoylmalic acid	1.109	0.030	↓	0.030
8.146	neg	PKODiA-PG	1.108	0.039	↓	<0.001
13.042	pos	2-Diethylaminoethanol	1.104	0.580	↓	0.011
5.161	neg	dhas#18	1.102	0.036	↓	0.005
10.258	pos	8-methyl-hexadecanedioic acid	1.101	0.390	↓	0.020
14.259	pos	Gentisic acid	1.101	0.527	↓	0.006
1.886	pos	3-Diphosphoglyceric acid	1.098	0.542	↓	0.010
10.192	pos	Dimethylsphingosine	1.097	0.572	↓	0.013

8.056	neg	Imiglitazar	1.096	0.002	↓	0.005
8.759	neg	Veranisatin A	1.096	0.557	↓	0.032
4.897	pos	Chapso	1.093	0.024	↓	0.021
12.713	pos	DG(20:4n6/0:0/22:2n6)	1.092	0.638	↓	0.016
5.701	neg	Dihydroneopterin phosphate	1.091	0.473	↓	0.006
8.834	pos	Nobiletin	1.090	0.042	↓	0.038
3.578	pos	Pentanamide	1.090	0.552	↓	0.008
10.963	neg	(3R)-10,13-Dimethyl-2,3,4,5,6,7,8,9,11,12,14,15,16,17-	1.089	0.186	↓	0.010
3.695	pos	Acipimox	1.088	0.582	↓	0.016
5.580	pos	(-)-lactol	1.087	0.171	↓	0.006
2.425	pos	Piracetam	1.087	0.503	↓	0.029
12.049	pos	Amifloxacin	1.086	0.468	↓	0.003
10.051	neg	AS-252424	1.085	0.656	↓	0.037
13.452	pos	N-Ethyl trans-2-cis-6-nonadienamide	1.083	0.558	↓	0.010
3.840	neg	Hyaluronan biosynthesis, precursor 1	1.079	0.062	↓	0.010
10.949	pos	Goshuyic acid	1.076	0.534	↓	<0.001
6.893	neg	3-O(4-O-beta-D-glucopyranosyl-alpha-L-rhamnopyranosyl)-	1.074	0.033	↓	0.001
9.962	neg	2,2'-Dithiodipyridine	1.070	0.618	↓	0.030
8.433	neg	C75 trans	1.068	0.649	↓	0.025
7.851	pos	7-Ketodeoxycholic acid	1.063	0.458	↓	0.015
3.611	pos	arabinofuranosylguanine	1.062	0.238	↓	0.013
3.494	pos	2-Amino-3-(2-fluoro-3,4-dihydroxyphenyl)propanoic acid	1.059	0.373	↓	0.001
14.192	pos	2-Amino-4-methylpyridine	1.057	0.517	↓	0.007
5.768	pos	Bancroftinone	1.056	0.547	↓	0.013
6.718	neg	m-Chlorobenzoic acid	1.055	0.591	↓	0.024
0.987	pos	Vinylphosphonic acid	1.055	0.551	↓	0.010
4.202	neg	N1-trans-Feruloylagmatine	1.054	0.272	↓	0.006
12.222	pos	(R)-4A-(Ethoxymethyl)-1-(4-fluorophenyl)-6-((4-	1.053	0.501	↓	0.007
4.266	pos	Stillopsin	1.051	0.271	↓	0.037
9.440	pos	3,4',5,6,7-PENTAMETHOXYFLAVONE	1.050	0.018	↓	0.048
8.615	neg	PKODiA-PI	1.049	0.036	↓	0.009
6.625	pos	Tetraethyl pyrophosphate	1.047	0.320	↓	0.008
8.183	neg	CDP-DG(22:6(4Z,7Z,10Z,13E,15E,19Z)-OH(17)/i-15:0)	1.043	0.154	↓	0.006
4.814	pos	6-beta-D-Glucopyranosyl-4',5-dihydroxy-3',7-	1.043	0.150	↓	0.007
5.613	neg	Fluocortolone Pivalate	1.042	0.269	↓	0.007
4.098	pos	3,4-Dihydroxyphenylethanol-4-diglucoside	1.040	0.018	↓	0.006
8.263	pos	20-trifluoro-LTB4	1.040	0.439	↓	0.004
12.364	pos	5alpha-Androsta-16-ene-3-ol	1.039	0.277	↓	0.034
5.966	neg	Egonol glucoside	1.038	0.124	↓	0.013
5.012	pos	bayogenin 3-O-cellobioside	1.037	0.092	↓	0.004
12.228	neg	2-METHYL GRAMINE	1.036	0.088	↓	0.002
4.953	neg	Glucocapparin	1.036	0.344	↓	0.004
10.446	pos	4E,14Z-Sphingadiene	1.035	0.629	↓	0.032
6.201	neg	p-hydroxybenzoylecgonine	1.034	0.064	↓	0.007

4.292	neg	Verbasoside	1.028	0.390	↓	0.016
3.306	pos	Isobutyryl-L-carnitine	1.025	0.376	↓	0.020
13.178	pos	n-arachidonylethanolamine	1.024	0.438	↓	0.010
9.356	pos	trans-feruloyl-CoA	1.023	0.086	↓	0.002
8.522	pos	Soyasapogenol B 3-O-b-D-glucuronide	1.021	0.135	↓	0.002
7.203	pos	8,9-Dihydro-5-hydroxy-8-(1-hydroxy-1-methylethyl)-6-(2-	1.020	0.421	↓	0.005
5.768	pos	Musabalbisiane C	1.020	0.281	↓	0.003
7.179	neg	Chitotriose	1.019	0.523	↓	0.007
6.093	pos	PIP(18:1(9Z)-O(12,13)/22:3(10Z,13Z,16Z))	1.018	0.063	↓	0.003
5.990	pos	Delamanid	1.013	0.110	↓	0.010
9.373	pos	Mono-(2-ethyl-5-hydroxyhexyl) phthalate	1.013	0.488	↓	0.030
4.098	pos	Sarcoehrendin A	1.012	0.269	↓	0.010
9.149	neg	Deoxycholic acid glycine conjugate	1.012	0.052	↓	0.003
9.628	neg	Methyl 3,5-dinitrobenzoate	1.010	0.664	↓	0.034
4.801	neg	6-[4-(2-carboxyethyl)phenoxy]-3,4,5-trihydroxyoxane-2-	1.005	0.242	↓	0.029
8.488	neg	Lisinopril-tryptophan	1.005	0.208	↓	0.016
8.159	pos	3alpha,12alpha-Dihydroxy-5beta-cholest-24-en-26-oic acid	1.003	0.092	↓	0.001
12.652	neg	DG(17:2(9Z,12Z)/18:3(9Z,12Z,15Z)/0:0)[iso2]	1.002	0.667	↓	0.040
8.194	pos	Saponin E	1.002	0.121	↓	0.005
10.949	pos	Linezolid	1.001	0.504	↓	0.001
5.204	pos	Aflatoxin B1 dialcohol	14.886	1.987	↑	0.045
4.445	pos	2,8-Quinolinediol	11.341	2.930	↑	0.001
5.802	pos	(10S,13S,16R)-d14-9-PhytoF[9S,12S]	11.220	1.907	↑	0.012
6.609	pos	Traumatic acid	10.658	1.671	↑	0.004
4.818	neg	3-(3-Hydroxyphenyl)propanoic acid	10.002	4.441	↑	0.037
5.228	neg	Azelaic acid	8.366	3.858	↑	0.027
7.489	pos	Dodecanedioic acid	8.013	2.170	↑	0.025
8.314	pos	5,8,12-Trihydroxy-9-octadecenoic acid	7.758	1.983	↑	0.001
5.055	neg	4-Hydroxy-5,7,4'-trimethoxyflavan	7.689	2.647	↑	<0.001
5.060	pos	Kuhlmanniquinol	7.443	2.310	↑	0.001
4.132	pos	Phenylalanylproline	7.314	3.098	↑	0.013
5.966	neg	Americanol A	7.044	4.916	↑	0.003
3.222	pos	Styrene	6.445	4.123	↑	0.001
3.931	pos	Quinaldine	6.369	2.263	↑	0.035
7.305	neg	13,16-Epoxy-7,12-dihydroxy-9Z-octadecenoic acid	6.214	1.548	↑	0.004
4.953	neg	4-Hydroxycinnamic acid	6.022	2.448	↑	0.036
4.450	neg	4-Acetamidobenzoic acid	5.791	2.546	↑	0.001
4.527	pos	xi-2,3-Dihydro-2-oxo-1H-indole-3-acetic acid	5.733	6.294	↑	0.028
6.980	pos	9,10,18-TriHOME(12Z)	5.717	1.438	↑	0.015
4.332	pos	(3b,4b,11b,14b)-11-Ethoxy-3,4-epoxy-14-hydroxy-12-cyathen-	5.479	3.330	↑	0.028
1.320	pos	Deoxyadenosine	5.452	2.933	↑	0.001
1.267	neg	Inosine	5.410	4.538	↑	<0.001
5.140	pos	Ciclopirox	5.111	2.703	↑	0.020
4.801	neg	Linusic acid	5.107	2.010	↑	<0.001

5.219	pos	Cinncassiol A	5.059	1.806	↑	0.015
5.264	pos	Dodecyl glucoside	4.962	2.089	↑	0.001
1.446	neg	Deoxyinosine	4.958	2.679	↑	0.015
8.974	pos	(9S,10E,12S,13S)-9,12,13-Trihydroxy-10-octadecenoic acid	4.762	1.492	↑	0.005
4.798	pos	4,1-Benzoxazepine	4.696	2.617	↑	<0.001
4.381	neg	Thonzylamine	4.438	6.599	↑	0.010
5.436	neg	Salicylic acid	4.387	3.200	↑	0.020
5.413	pos	2,3-dinor-PGE1	4.379	1.567	↑	0.012
11.751	pos	Heliantriol F	4.281	2.033	↑	0.022
10.585	pos	LysoPE(0:0/16:0)	4.157	2.289	↑	0.014
0.644	pos	Pirlindole	4.105	5.117	↑	0.002
5.895	neg	ORTHOTHYMOTINIC ACID	4.090	2.259	↑	0.017
0.754	pos	N-Acetylcadaverine	4.023	3.252	↑	0.012
5.249	pos	3-(2-Furanylmethyl)-1H-pyrrole	4.003	2.511	↑	0.001
4.818	neg	7Z-Decenyl acetate	3.916	2.040	↑	0.006
4.798	pos	bhos#20	3.884	1.775	↑	0.002
4.292	neg	2-Pyrocatechuic acid	3.873	3.303	↑	0.008
6.367	pos	Cucujolide IV	3.867	1.594	↑	0.001
9.044	pos	9,10-Epoxyoctadecanoic acid	3.865	1.635	↑	0.001
7.320	pos	19-hydroxyprostaglandin H2(1-)	3.830	1.675	↑	<0.001
4.521	neg	2-(3-Hydroxy-1H-indol-2-yl)acetic acid	3.824	6.214	↑	0.023
2.097	neg	4-Amino-1-[(2R,3S,4S,5R)-3,4-dihydroxy-5-	3.818	2.581	↑	0.001
4.382	pos	Corey PG-Lactone Diol	3.814	4.260	↑	0.012
4.717	pos	(3S,7S)-Jasmonic acid	3.796	2.066	↑	0.002
7.394	neg	9-hydroperoxy-12,13-dihydroxy-10-octadecenoic acid	3.736	1.691	↑	<0.001
5.204	pos	bhas#20	3.725	2.095	↑	0.004
5.187	pos	Entecavir	3.669	1.692	↑	0.004
11.488	neg	5-Hexyltetrahydro-2-furanoctanoic acid	3.588	1.813	↑	0.016
4.445	pos	Flazine methyl ether	3.568	4.431	↑	0.001
5.413	pos	oscr#19	3.562	2.004	↑	0.002
6.344	neg	8-Hydroxy-4,8-dimethyl-4E,9-decadienoic acid	3.500	2.273	↑	0.001
4.588	pos	7-Epi-12-hydroxyjasmonic acid	3.460	2.118	↑	0.011
5.430	pos	Dehydromatricaric acid	3.395	4.527	↑	0.009
2.113	pos	5,7,3',4',5'-Pentahydroxyflavanone	3.384	2.593	↑	0.003
7.336	pos	PE-Cer(d14:1(4E)/16:0)	3.382	10.05	↑	0.022
4.717	pos	p-Acetaminobenzaldehyde	3.311	2.227	↑	0.015
5.382	pos	(1R,2R,4R,5S)-(+)-p-Menthane-2,5-diol	3.278	2.041	↑	0.005
5.228	neg	(S)-9-Hydroxy-10-undecenoic acid	3.252	3.376	↑	0.002
4.818	neg	Quercetin 3-O-sophoroside	3.250	6.185	↑	0.036
10.740	pos	N-3-hydroxy-13-methyl-hexadecanoyl glycyl-L-serine	3.212	3.230	↑	0.001
4.542	pos	Dabigatran	3.192	3.441	↑	0.026
5.211	neg	Lucidenic acid E2	3.168	4.938	↑	0.001
5.489	neg	4'-Dihydroabscisic acid	3.164	2.347	↑	<0.001
6.248	pos	Hydantoin-5-propionic acid	3.123	1.972	↑	0.023

5.785	pos	Mycinonic acid III	3.102	1.950	↑	0.005
6.896	pos	Enterolactone	3.052	1.860	↑	0.020
9.878	pos	LysoPE(15:0/0:0)	3.021	3.774	↑	0.001
0.768	pos	Monopropionylcadaverine	3.020	5.663	↑	0.006
5.295	neg	Gibberellin A59	3.018	3.472	↑	0.002
4.801	neg	Naringenin 7,4'-dimethyl ether	2.991	3.461	↑	0.005
7.269	neg	Ipomeatetrahydrofuran	2.986	2.025	↑	0.006
4.183	pos	1,2-Dihydronaphthalene-1,2-diol	2.963	2.441	↑	0.011
1.067	pos	4-(Aminomethyl)-1-methylpiperidin-4-ol	2.940	3.240	↑	0.008
0.754	pos	[(2R,3S,4R,5R)-3,4,5,6-Tetrahydroxy-1-oxohexan-2-yl] (2S)-	2.931	2.508	↑	0.001
7.303	pos	6-Hexyltetrahydro-2H-pyran-2-one	2.928	1.982	↑	0.013
5.195	neg	Glunicate	2.913	2.055	↑	0.019
8.882	neg	Brefeldin A	2.859	3.016	↑	0.003
1.267	neg	Glucose pyruvate lactate	2.855	3.850	↑	<0.001
6.794	pos	oscr#24	2.821	2.111	↑	0.015
6.127	pos	Indole-3-propionic acid	2.820	3.587	↑	0.001
10.652	pos	LysoPE(18:1(9Z)/0:0)	2.809	1.891	↑	0.029
4.946	pos	(3S,5R,6R,7E)-3,5,6-Trihydroxy-7-megastigmen-9-one	2.802	2.682	↑	0.004
14.290	neg	Schisanhenol	2.772	1.978	↑	0.002
5.331	neg	4-Oxododecanedioic acid	2.761	2.248	↑	0.001
1.446	neg	2-(2-Oxopropanoyloxy)propanoic acid	2.758	2.467	↑	0.009
3.459	pos	N-(5-ACETAMIDOPENTYL)ACETAMIDE; Pentamethylene	2.692	2.352	↑	0.006
5.211	neg	Myotoxin A	2.691	4.446	↑	0.002
6.007	pos	Fumonisin B1	2.652	3.046	↑	0.031
5.471	neg	Batatasin IV	2.644	2.137	↑	0.004
5.005	neg	5,7-Dimethoxy-3',4'-methylenedioxy-4-phenylcoumarin	2.634	3.029	↑	0.019
4.847	pos	Norbutorphanol	2.620	5.859	↑	0.022
1.192	pos	Adenosine	2.613	1.767	↑	0.047
5.382	pos	5-Isopropylbicyclo[3.1.0]hexan-2-one	2.571	1.739	↑	0.006
5.005	neg	Obtustyrene	2.538	3.208	↑	0.008
5.055	neg	2H-1-Benzopyran-2-one, 7-[[2-(acetylamino)-2-deoxy-beta-D-	2.536	3.993	↑	<0.001
4.627	neg	ascr#6.1	2.530	2.382	↑	0.013
5.436	neg	3,8-Dihydroxy-1-methylanthraquinone-2-carboxylic acid	2.527	4.327	↑	0.046
5.055	neg	Cyclo(-D-Tyr-Arg-Gly-Asp-Cys(carboxymethyl)-OH)	2.513	3.755	↑	0.003
4.493	pos	Guaiaretic acid	2.485	3.399	↑	0.001
5.211	neg	Cycloartomunoxanthone	2.440	2.076	↑	0.036
5.367	neg	(E)-2-Glucosyl-3,4',5-trihydroxystilbene	2.437	2.349	↑	0.019
5.563	pos	3-Hydroxydodecanedioic acid	2.432	2.183	↑	0.009
4.685	pos	Diethylene glycol dimethacrylate	2.428	4.295	↑	0.047
7.320	pos	12-oxo-PDA	2.428	1.403	↑	0.012
4.681	neg	Phenol A	2.415	8.229	↑	0.049
6.742	pos	N,N,O-Tridesmethylvenlafaxine	2.412	2.356	↑	0.049
5.228	neg	Hydramethylnon	2.408	3.371	↑	0.041
5.546	pos	Polyethylene, oxidized	2.405	1.767	↑	0.008

4.818	neg	oscr#7	2.401	3.821	↑	0.005
4.132	pos	5-Amino-2,3-dihydro-6-(3-hydroxy-4-methoxy-1-oxobutyl)-	2.389	3.478	↑	0.004
5.854	pos	N,N,O-Tridesmethyltramadol	2.383	2.412	↑	0.020
4.539	neg	Phaseolic acid	2.383	3.758	↑	0.023
8.687	neg	oscr#17	2.374	1.608	↑	<0.001
4.768	neg	3,10-dihydroxydecanoic acid	2.371	2.726	↑	0.002
4.936	neg	oscr#14	2.368	1.617	↑	0.002
9.878	pos	(2R,3R)-2-Aminooctadecane-1,3-diol	2.365	3.508	↑	0.001
5.060	pos	PS(O-16:0/0:0)	2.351	1.484	↑	0.036
5.560	neg	Hexyl glucoside	2.350	3.790	↑	0.012
5.228	neg	Scillaren A	2.325	3.317	↑	0.003
4.814	pos	S-4-Hydroxymephenytoin	2.309	2.498	↑	0.004
4.936	neg	5,8-Dihydroxy-3-(4-hydroxybenzyl)-7-methoxy-4-chromanone	2.307	2.992	↑	0.002
1.460	pos	Conhydrinone	2.299	4.191	↑	0.007
6.646	neg	3-hydroxyl-2',5-dimethoxy-2-methylbibenzyl	2.289	1.888	↑	0.006
10.828	pos	LysoPE(P-16:0/0:0)	2.273	2.676	↑	0.022
4.292	neg	Blighinone	2.270	10.44	↑	0.029
12.730	pos	FAHFA(16:0/14-O-18:0)	2.266	2.052	↑	0.045
5.228	neg	Ethyl 7-epi-12-hydroxyjasmonate glucoside	2.257	5.555	↑	0.034
5.546	pos	Monic acid	2.255	1.654	↑	0.006
6.692	pos	Prostalene	2.247	1.451	↑	0.020
5.413	pos	9-epi-9-F1t-PhytoP	2.238	1.628	↑	0.004
6.248	pos	dapsone hydroxylamine	2.211	2.010	↑	0.013
5.887	pos	Tetraethylene glycol monododecyl ether	2.210	1.820	↑	0.003
4.847	pos	Glyceryl 5-hydroxydecanoate	2.204	2.459	↑	0.003
3.569	neg	Telotristat	2.184	4.271	↑	0.001
7.336	pos	Secoeremopetasitolide B	2.182	2.023	↑	0.001
12.049	pos	Glycyrrhetol	2.138	1.745	↑	0.024
5.028	pos	2,3-Dihydroxypropyl octanoate	2.125	1.725	↑	0.005
5.060	pos	ascr#13	2.095	1.956	↑	0.011
6.214	pos	Sorbitan laurate	2.068	2.269	↑	0.006
4.382	pos	3-amino-2-naphthoic acid	2.063	2.044	↑	0.006
4.715	neg	3-Methyldioxyindole	2.063	2.024	↑	0.024
3.339	pos	Hydroxy Tyrosol -Acetate	2.061	1.934	↑	0.031
6.025	pos	(10E,15Z)-9,12,13-Trihydroxyoctadeca-10,15-dienoylcarnitine	2.043	2.877	↑	0.004
6.997	pos	DG(i-12:0/20:5(7Z,9Z,11E,13E,17Z)-3OH(5,6,15)/0:0)	2.033	7.283	↑	0.026
7.287	neg	VD 2656	2.030	3.854	↑	0.006
7.465	neg	Dimethindene	2.026	3.972	↑	<0.001
0.553	pos	Spermidine	2.025	2.871	↑	0.021
1.267	neg	Deoxyuridine	2.025	2.984	↑	0.003
4.798	pos	5,3'-Dihydroxy-7,8,4'-trimethoxyflavanone	2.015	2.869	↑	0.010
11.610	pos	7-Hydroxyoctanoylcarnitine	2.009	1.666	↑	0.050
1.321	neg	9H-Purine-9-butanoic acid, 6-amino-alpha-hydroxy-, methyl	2.005	3.491	↑	<0.001
4.460	pos	Brevianamide B	2.004	6.751	↑	0.019

4.767	pos	(Z)-N-Feruloyl-5-hydroxyanthranilic acid	1.996	2.362	↑	0.009
8.040	pos	Turpetholic acid A	1.992	2.498	↑	0.004
6.344	neg	Altamisic acid; 3-[(3aS,8R,8aR)-8-Hydroxy-6,8-dimethyl-3-	1.991	1.999	↑	0.001
8.143	pos	11,12-dihydroxy arachidic acid	1.983	1.428	↑	0.014
4.445	pos	icos#17	1.974	2.245	↑	0.016
5.613	neg	3,4-DHPEA-EA	1.971	1.967	↑	0.005
5.754	neg	Involucrin	1.969	3.165	↑	0.002
1.398	pos	Noralfentanil	1.942	18.61	↑	0.013
1.445	pos	n6,2'-o-dibutyryladenosine	1.936	2.825	↑	0.006
10.482	pos	Ganoderiol A	1.917	3.283	↑	0.035
4.750	neg	Phenmedipham	1.906	2.491	↑	0.006
5.005	neg	2'-Hydroxy-7-methoxy-4',5'-methylenedioxyflavan	1.905	2.530	↑	0.024
4.685	pos	Methyl 12-hydroxyjasmonate	1.901	1.988	↑	<0.001
7.489	pos	3,7,7,10-Tetramethyl-12-thiabicyclo[9.1.0]dodeca-3,7-diene	1.897	1.772	↑	0.023
6.248	pos	Z-Ala-ONp	1.894	1.825	↑	0.048
5.195	neg	Bastaxanthin C	1.889	3.561	↑	0.002
9.009	pos	N-linoleoyl dopamine	1.887	7.352	↑	0.048
6.896	pos	N-Ethyl-N-Methylcathinone	1.872	2.320	↑	0.046
10.296	neg	1-(2-methoxy-tetradecanyl)-sn-glycero-3-	1.868	3.908	↑	0.008
6.197	pos	2,3-Dihydroxypropyl 4-phenylbutanoate	1.856	1.847	↑	0.046
8.020	neg	1-dodecanoyl-sn-glycerol	1.850	2.874	↑	0.003
5.005	neg	Fluridone	1.842	3.498	↑	0.021
4.588	pos	9,10-dihydroxy-2-decenoic acid	1.841	1.956	↑	0.020
5.613	neg	20-Hydroxy-PGE2	1.827	1.600	↑	0.002
6.876	neg	Roxadustat	1.815	1.915	↑	0.006
5.055	neg	mezerein	1.808	3.994	↑	<0.001
5.447	pos	L-Dihydroorotic acid	1.797	3.692	↑	0.033
11.717	pos	PE-Cer(d14:2(4E,6E)/16:0(2OH))	1.796	2.787	↑	0.002
0.769	neg	Elephantorrhizol	1.795	3.682	↑	<0.001
7.481	neg	3-[4-[(E)-2-[6-(Dibutylamino)naphthalen-2-yl]ethenyl]pyridin-	1.779	2.267	↑	0.008
8.326	neg	2,15-dihydroxy-pentadecylic acid	1.778	3.056	↑	0.003
7.465	neg	bhos#42	1.778	7.852	↑	0.001
4.831	pos	Jasmine ketolactone	1.771	2.722	↑	0.007
2.757	pos	Indoleacetaldehyde	1.767	5.089	↑	0.004
6.334	pos	11-Hydroxy-9-tridecenoic acid	1.761	1.801	↑	0.005
4.527	pos	4-Hydroxyquinoline	1.755	4.430	↑	0.034
3.222	pos	Assafoetidin	1.746	3.707	↑	0.002
7.539	pos	Phenylalanyl-prolyl-arginine	1.745	1.975	↑	0.010
1.644	neg	Baohuoside I	1.740	3.798	↑	0.039
7.473	pos	N-Oleoyl Glutamine	1.738	2.504	↑	0.041
6.858	neg	PI(20:1(11Z)/0:0)	1.726	3.230	↑	0.004
2.113	pos	Ac-DEVD-CHO	1.704	10.10	↑	0.032
4.801	neg	Methyl helianthenoate A glucoside	1.703	4.131	↑	0.029
5.022	neg	6-Ketoestriol	1.699	2.096	↑	0.042

7.336	pos	Nummularine A	1.690	7.667	↑	0.025
0.584	neg	Allitridin	1.687	1.901	↑	0.012
1.446	neg	Carbutamide	1.684	2.650	↑	0.012
5.524	neg	Daumone	1.665	2.306	↑	<0.001
5.228	neg	Nanaomycin	1.665	3.657	↑	0.014
6.811	pos	9,10-dihydroxy-13-hydroperoxy-11-octadecenoic acid	1.656	1.837	↑	<0.001
11.751	pos	Theonellasterol B	1.644	1.666	↑	0.033
4.282	pos	Crocin 3	1.640	1.915	↑	0.020
4.818	neg	trans-p-Menthane-7,8-diol 7-glucoside	1.632	1.912	↑	0.001
4.818	neg	Vanillin 3-(L-menthoxy)propane-1,2-diol acetal	1.632	6.147	↑	0.019
1.464	neg	4-Pyridoxic acid	1.626	3.816	↑	0.007
12.484	neg	Sandosapogenol	1.624	1.766	↑	0.043
5.956	pos	17-Hydroxypregnenolone sulfate	1.622	2.155	↑	0.030
8.332	pos	9,13-dihydroxy-10-ethoxy-11-octadecenoic acid	1.618	1.943	↑	0.001
5.187	pos	(3beta,9beta)-7-Drimene-3,11,12-triol	1.615	1.967	↑	0.017
6.454	pos	1a,1b-dihomo-PGD2	1.614	3.155	↑	0.040
4.539	neg	5,4'-Dihydroxy-7-methoxy-8-methylflavanone	1.606	2.450	↑	0.029
4.574	neg	Toxin T2 tetrol	1.602	1.905	↑	0.009
6.214	pos	Aleuritic acid	1.600	2.667	↑	0.001
10.175	pos	LysoPE(0:0/15:0)	1.599	3.174	↑	<0.001
4.801	neg	19-Noraldosterone	1.597	9.184	↑	0.016
5.055	neg	Annomuricatin A	1.582	5.321	↑	0.001
11.295	pos	N-(15-methyl-3-hydroxy-hexadecanoyl)-glycine	1.576	2.784	↑	0.002
10.035	pos	Sphingosine	1.575	5.047	↑	0.002
7.305	neg	Kurilensoside G	1.574	4.376	↑	0.001
3.948	pos	Dibucaine	1.573	4.478	↑	0.012
3.350	neg	Catechin 5,7,3'-trimethyl ether	1.567	4.100	↑	<0.001
11.173	neg	LysoPS(16:0/0:0)	1.566	7.210	↑	0.002
6.692	pos	Equol	1.565	2.344	↑	0.001
3.356	pos	3,5,7-Trimethylepicatechin	1.561	3.859	↑	<0.001
7.489	pos	Cholylarginine	1.558	3.195	↑	0.002
5.752	pos	4,11,13,15-Tetrahydroridentin B	1.558	1.683	↑	0.006
0.768	pos	Fructose lactate	1.552	2.908	↑	0.001
4.542	pos	Vignatic acid B	1.543	4.512	↑	0.022
5.701	neg	Enterodiol	1.539	2.824	↑	0.001
7.481	neg	N'-Hydroxysaxitoxin	1.539	2.112	↑	0.012
8.020	neg	1-(3-Furanyl)-6,7-dihydroxy-4,8-dimethyl-1-nonanone	1.536	1.918	↑	0.006
6.486	neg	3-(1,1-Dimethylallyl)herniarin	1.528	4.751	↑	<0.001
10.192	pos	N-(m-Methoxybenzyl)hexadecanamide	1.527	11.81	↑	0.001
4.946	pos	Homoveratric acid	1.521	3.224	↑	0.044
5.419	neg	(S)-p-Menth-1-ene-4,7-diol 4-glucoside	1.512	2.399	↑	0.001
2.162	pos	1-Methyladenosine	1.510	3.087	↑	0.027
12.312	pos	Momorcharaside B	1.510	2.571	↑	0.003
4.381	neg	Cyclohexyladenosine	1.507	13.81	↑	0.009

8.882	neg	1-[(1R,2R,3S,4S)-3-Hydroxy-4,7,7-trimethyl-2-	1.502	2.155	↑	0.003
5.211	neg	Strophanthin	1.477	4.075	↑	0.002
4.733	pos	Dactimicin	1.471	1.733	↑	0.006
1.231	neg	True blue	1.467	4.752	↑	0.001
10.106	pos	Tuberculostearic acid	1.465	3.391	↑	0.001
4.299	pos	Avenanthramide E	1.462	2.303	↑	0.019
10.361	pos	LysoPS(18:2(9Z,12Z)/0:0)	1.458	5.319	↑	0.006
3.569	neg	N-(4-((5-(3-(2-Aminoethyl)-1H-indol-5-yl)-1,2,4-oxadiazol-3-	1.452	1.656	↑	0.022
1.411	neg	N-Succinyl-2-amino-6-ketopimelate	1.449	2.887	↑	0.019
5.666	neg	Abscisic alcohol 11-glucoside	1.447	1.599	↑	0.014
4.913	pos	galactosyl hydroxylysine	1.446	1.711	↑	0.003
4.573	pos	Talaromycin A	1.445	2.744	↑	0.010
0.613	pos	N1-Acetylspermidine	1.441	2.833	↑	0.039
7.018	neg	2,6-Diaminopurine 2',3'-dideoxyriboside	1.438	1.719	↑	0.015
4.542	pos	5,2'-Dihydroxy-7,4',5'-trimethoxyflavanone	1.438	1.987	↑	0.032
4.750	pos	N-nonanoyl-L-Homoserine lactone	1.436	2.302	↑	0.016
6.876	neg	Ononin	1.434	1.655	↑	0.015
6.254	neg	2-(L-Menthoxy)ethanol	1.433	3.623	↑	0.002
5.243	neg	1-[Ethyl-(6-hydrazinylpyridazin-3-yl)amino]propan-2-ol	1.420	1.444	↑	0.004
6.110	pos	Tetranor-PGF1alpha	1.414	1.648	↑	0.005
3.998	pos	Phenylacetylglutamine	1.413	1.780	↑	0.027
5.044	pos	Cimifugin	1.412	2.187	↑	0.029
4.867	neg	N-Palmitoyl Cysteine	1.412	3.892	↑	0.009
5.436	neg	Epimedokoreanin A	1.409	3.363	↑	0.015
5.802	pos	Sebacoyl-L-carnitine	1.407	2.523	↑	0.012
5.413	pos	Tatridin B	1.407	1.509	↑	0.006
4.604	pos	Tensyuic acid E	1.404	1.750	↑	0.004
5.966	neg	o-Cresolphthalein complexone	1.403	34.00	↑	0.025
5.177	neg	Xamoterol	1.402	7.374	↑	<0.001
5.973	pos	Blennin D	1.402	1.662	↑	0.001
9.339	pos	Dehydrophytosphingosine	1.402	2.872	↑	0.002
6.794	pos	3-Epiaphidicolin	1.400	1.862	↑	0.005
7.573	pos	PGE2-EA	1.398	1.504	↑	0.032
5.481	pos	1,3,7-Trimethyl-8-nonylpurine-2,6-dione	1.398	1.719	↑	0.020
8.974	pos	3-(1-Propyl-3-piperidinyl)phenol	1.397	2.585	↑	0.016
7.287	neg	DG(18:1(12Z)-2OH(9,10)/8:0/0:0)	1.394	3.986	↑	0.007
5.887	pos	19-Hydroxyprostaglandin F	1.392	2.152	↑	0.001
7.489	pos	Pinazepam	1.390	1.857	↑	0.011
4.588	pos	3alpha,7alpha,12alpha-trihydroxy-5alpha-cholan-24-yl sulfate	1.386	5.298	↑	<0.001
12.696	pos	PE-Cer(d14:2(4E,6E)/18:0(2OH))	1.386	2.632	↑	0.002
6.002	neg	Gibberellin A50	1.384	3.780	↑	0.001
5.219	pos	Macrolactin O	1.382	3.984	↑	0.001
7.557	pos	Pteroside Z	1.381	1.803	↑	0.002
4.485	neg	7-Hydroxy-5-methoxy-4-methyl-3-(4-methylpiperazin-1-yl)-	1.380	3.220	↑	<0.001

4.510	pos	Myricanol 5-beta-sophoroside	1.380	2.725	↑	0.004
6.111	neg	oscr#16	1.379	1.763	↑	0.001
7.456	pos	DG(20:5(5Z,8Z,11Z,14Z,17Z)/22:6(4Z,7Z,10Z,13Z,16Z,19Z)/	1.377	4.812	↑	0.012
2.186	neg	Dibutyryl cyclic gmp	1.375	5.075	↑	<0.001
5.280	pos	(1beta,8beta)-1,8-Dihydroxy-3,7(11)-eudesmadien-12,8-olide	1.375	1.796	↑	0.012
1.267	neg	Alginic acid	1.374	4.198	↑	<0.001
4.521	neg	Glucosamine 6-phosphate	1.374	3.494	↑	0.016
6.367	pos	(-)-Perillyl alcohol	1.373	1.656	↑	0.001
6.504	neg	MG(0:0/10:0/0:0)	1.370	3.505	↑	0.004
7.481	neg	PA(18:4(6Z,9Z,12Z,15Z)/18:4(6Z,9Z,12Z,15Z))	1.369	2.713	↑	0.026
5.187	pos	Bisacurone epoxide	1.363	1.746	↑	0.034
8.784	pos	ubiquinone-8	1.359	1.794	↑	0.004
5.471	neg	Abyssinoflavanone IV	1.358	4.745	↑	0.004
3.948	pos	Spermine dialdehyde	1.357	1.949	↑	0.026
7.203	pos	Fumonisin B2	1.356	3.759	↑	0.021
6.592	pos	(Z)-5-((2R,3S,4S,6R)-4,6-Dihydroxy-2-((S,E)-3-hydroxyoct-1-	1.356	1.699	↑	0.034
5.436	neg	2-Hydroxy-4-trifluoromethyl benzoic acid	1.356	2.570	↑	0.033
5.060	pos	Penilloic acid	1.352	2.328	↑	0.024
6.700	neg	Sativic acid	1.347	1.678	↑	0.013
11.712	neg	Iprovalicarb	1.345	3.192	↑	0.001
7.473	pos	Neofusapyrone	1.336	4.438	↑	0.004
7.287	neg	Lobophysterol B	1.335	3.443	↑	0.007
5.560	neg	2-Hydroxydecanedioic acid	1.334	2.495	↑	0.024
4.493	pos	1,2-Diphenylhydrazine	1.332	4.121	↑	0.027
4.416	neg	Pisumic acid	1.323	3.176	↑	0.012
5.144	neg	(1S,3R,4R)-p-Menthane-1,3-diol	1.323	2.026	↑	<0.001
5.092	pos	2alpha-Hydroxyalantolactone	1.321	1.664	↑	0.012
5.454	neg	omega-hydroxy enanthoic acid	1.320	2.863	↑	0.037
4.381	neg	2-Amino-5-phenylpyridine	1.317	2.101	↑	0.002
1.605	pos	Ribothymidine	1.317	1.992	↑	0.048
7.987	pos	(3R,5R)-7-[(1S,2R,3R,8S,8Ar)-3-hydroxy-2-methyl-8-(2-	1.309	6.567	↑	0.006
5.249	pos	3-(4-Methyl-3-pentenyl)thiophene	1.307	2.560	↑	0.001
4.557	neg	Darexaban glucuronide	1.304	2.474	↑	0.003
0.769	neg	Neuraminic acid	1.302	2.281	↑	0.032
4.814	pos	4,4'-Thiobis(6-tert-butyl-m-cresol)	1.299	1.474	↑	0.008
5.984	neg	Fumonisin C4	1.298	3.747	↑	0.026
9.847	pos	1alpha-hydroxy-24,25,26,27-tetranorvitamin D3 / 1alpha-	1.296	7.184	↑	0.003
11.488	pos	alpha-Tocotrienoxyl radical	1.294	1.861	↑	0.003
3.140	pos	Procaine	1.293	2.788	↑	0.018
4.715	neg	Cyclocalopin E	1.284	2.330	↑	0.007
4.249	pos	(2S,3S)-2-Amino-N-benzyl-3-methylpentanamide	1.282	4.698	↑	0.015
7.015	pos	Lyngbic acid	1.280	2.277	↑	0.002
4.645	neg	3-O-(beta-D-glucopyranosyl)-3beta,14beta-dihydroxy-16beta-	1.279	2.991	↑	0.006
7.489	pos	Nomilinic acid	1.271	2.453	↑	0.029

4.382	pos	FS4 toxin	1.270	2.983	↑	0.013
5.820	pos	cis-Piceid	1.270	1.932	↑	0.047
4.913	pos	Inproquone	1.269	2.482	↑	0.031
5.313	neg	Sumarotene	1.269	1.607	↑	<0.001
9.543	pos	C17 Sphinganine	1.268	3.813	↑	0.001
7.730	pos	DG(13:0/PGE2/0:0)	1.268	58.36	↑	0.045
2.641	pos	Simmondsin	1.263	3.606	↑	0.020
6.468	neg	3,11,12-Trihydroxy-1(10)-spirovetiven-2-one	1.262	2.055	↑	<0.001
5.754	neg	Monoethyl phthalate	1.262	1.847	↑	0.046
4.366	pos	Berkeleylactone !	1.257	1.946	↑	0.003
6.020	neg	bhos#16	1.255	1.932	↑	0.001
1.460	pos	(S)-3-Butyl-1(3H)-isobenzofuranone	1.255	4.040	↑	0.030
7.117	pos	Ipurolic acid	1.255	2.479	↑	0.001
5.211	neg	Rhizoxin	1.255	6.748	↑	0.039
5.060	pos	3'-Hydroxy-T2-triol	1.253	1.847	↑	0.036
5.384	neg	(S)-Spinacine	1.250	1.983	↑	<0.001
6.165	neg	(E)-2-Octen-1-ol	1.247	1.461	↑	0.006
5.513	pos	Tryptophol	1.247	2.024	↑	0.003
4.850	neg	9,10-Dihydro-8-hydroxy-10-methyl-8H-pyrano[2,3-	1.244	3.680	↑	0.004
4.332	pos	Indolepropionamide	1.241	2.668	↑	0.008
4.953	neg	indole-3-acetyl-glutamine	1.238	2.103	↑	0.007
5.091	neg	(R)-Pelletierine	1.237	2.849	↑	0.045
1.445	pos	3-Maleimidobenzoic acid N-hydroxysuccinimide ester	1.236	2.172	↑	0.032
11.591	neg	Prostaglandin D2 methyl ester	1.233	1.851	↑	0.017
2.097	neg	Sulfameter	1.231	2.941	↑	0.001
6.367	pos	hexadecanedioic acid mono-L-carnitine ester	1.230	2.749	↑	0.010
3.749	neg	1-(beta-D-Glucopyranosyloxy)-3-octanone	1.229	2.241	↑	0.002
3.222	pos	4-Amino-2,2,6,6-tetramethylpiperidin-1-ol	1.225	5.789	↑	0.009
4.750	neg	Ganoweberianic acid E	1.221	3.492	↑	0.004
2.575	pos	epsilon-heptenoic acid	1.218	1.948	↑	0.027
5.631	neg	Oryzalic acid B	1.215	1.300	↑	0.018
7.481	neg	3-Hydroxylidocaine	1.215	2.447	↑	0.012
6.913	pos	O-(11-Carboxyundecanoyl)carnitine	1.215	2.468	↑	0.006
10.227	neg	PA(0:0/16:0)	1.213	3.458	↑	0.001
5.195	neg	Lactarofulvene	1.212	2.254	↑	0.033
7.489	pos	1-(O-alpha-D-mannopyranosyl)-25-keto-(1,3R,27R)-	1.208	13.90	↑	0.047
11.279	neg	2S-amino-octadeca-4E,6E-diene-1,3R-diol	1.206	2.672	↑	0.002
4.681	neg	13-Hydroxyabscisic acid	1.206	2.091	↑	0.002
4.936	neg	Cinncassiol C3	1.204	2.004	↑	0.001
9.627	pos	Clupadonic acid	1.198	16.95	↑	0.002
5.878	neg	12-hydroxy-3Z,6Z-dodecadienoic acid	1.196	2.415	↑	0.005
5.615	pos	Aflatoxin ExB2	1.196	5.543	↑	0.005
3.306	pos	(2E,4E)-Hexa-2,4-dienedioylcarnitine	1.196	2.263	↑	0.002
5.195	neg	3-(6-((4-(Trifluoromethoxy)phenyl)amino)pyrimidin-4-	1.194	2.262	↑	0.022

4.493	pos	Semilepidinoside A	1.193	2.354	↑	0.001
4.880	pos	4-(4-Methylcyclohexyl)-4-oxobutanoic acid	1.192	2.131	↑	0.007
5.234	pos	2-Cyclohexylidenecyclohexanone	1.192	2.120	↑	0.008
5.397	pos	3,14-Dihydroxy-11,13-dihydrocostunolide	1.191	1.821	↑	0.005
5.091	neg	terpentecin	1.190	2.317	↑	<0.001
6.254	neg	5,7-Dihydroxy-4'-methoxy-8-methylflavanone	1.185	2.760	↑	0.008
7.179	neg	9alpha-(3-Methylbutanoyloxy)-4S-hydroxy-10(14)-oplopen-3-	1.178	2.166	↑	0.001
5.012	pos	Maximaisoflavone D	1.178	2.576	↑	0.041
4.834	neg	Ustusoic acid A	1.177	10.93	↑	0.035
4.363	neg	Leptin F	1.176	3.222	↑	0.002
5.563	pos	8-[(2R,3S)-3-(8-Hydroxyoctyl)oxiran-2-yl]octanoylcarnitine	1.174	3.081	↑	0.001
5.177	neg	(-)-Euphomine	1.174	3.063	↑	0.009
4.573	pos	Phe-Pro-Ile	1.171	2.296	↑	0.004
5.295	neg	Anacyclic acid	1.169	2.555	↑	<0.001
4.129	neg	Diphenyl(2,4,6-trimethylbenzoyl)phosphine oxide	1.169	2.263	↑	0.003
7.117	pos	4,14-dihydroxy-octadecanoic acid	1.169	1.712	↑	0.002
6.841	neg	14S-hydroxy-hexadecanoic acid	1.168	4.157	↑	0.001
4.460	pos	Siguazodan	1.167	2.190	↑	0.002
7.018	neg	oscr#3	1.167	2.144	↑	0.001
4.332	pos	Biotin	1.164	3.161	↑	0.007
5.560	neg	Glycerol 1-(5-hydroxydodecanoate)	1.163	2.761	↑	<0.001
7.694	pos	3,7-Dihydroxydecanoylcarnitine	1.163	1.690	↑	0.004
1.869	pos	Prolyl-Histidine	1.162	3.804	↑	0.003
5.296	pos	7-Hydroxyenterolactone	1.158	4.531	↑	0.012
4.485	neg	6b-Angeloyl-3b,8b,9b-trihydroxy-7(11)-eremophilen-12,8-	1.158	2.400	↑	0.001
4.233	pos	Isoleucyl-Tyrosine	1.157	3.108	↑	<0.001
7.920	pos	Diethylpropion	1.156	2.399	↑	0.012
5.039	neg	Tetranor-PGEM	1.155	1.624	↑	0.012
6.110	pos	10-Hydroxydecanoic acid	1.152	1.557	↑	0.001
4.637	pos	2-(3-Hydroxyphenyl)ethanol 1'-glucoside	1.152	2.079	↑	0.035
6.929	neg	gamma-CEHC	1.149	2.130	↑	<0.001
5.365	pos	Abscisic alcohol	1.147	1.804	↑	0.003
3.391	pos	Benzoyl L-arginine methyl ester	1.147	5.323	↑	0.007
5.039	neg	Isopentyl beta-D-glucoside	1.141	3.043	↑	0.006
11.505	pos	(3beta,23E)-3-Hydroxy-27-norcycloart-23-en-25-one	1.141	1.957	↑	0.006
5.055	neg	Halistanol sulfonic acid B	1.138	3.108	↑	0.002
9.575	pos	Kalkitoxin	1.136	2.292	↑	0.004
5.447	pos	Hydroxyprolyl-Cysteine	1.131	2.960	↑	0.024
5.808	neg	Marmesin galactoside	1.130	1.908	↑	0.017
4.785	neg	Alminoprofen	1.129	4.425	↑	0.004
4.031	pos	Nicotine glucuronide	1.128	1.909	↑	0.015
5.542	neg	5'-Amino-5'-deoxyadenosine	1.125	1.777	↑	0.016
7.305	neg	Exophilin A1	1.122	5.323	↑	<0.001
9.948	pos	1-(2-methoxy-6Z-tetradecenyl)-sn-glycero-3-phosphoserine	1.116	4.720	↑	0.006

0.644	pos	L-Asparagine	1.116	4.668	↑	0.003
6.646	neg	Nor-cocaine	1.114	2.415	↑	0.001
2.840	pos	N-(S-Nitroso-N-acetyl-D,L-penicillamine)-2-amino-2-deoxy-	1.113	4.861	↑	0.001
3.812	pos	dihydroxyphaseic acid	1.107	3.858	↑	0.012
3.915	pos	Centpropazine	1.107	2.859	↑	0.007
1.375	neg	dTDP-4-acetamido-4,6-dideoxy-D-galactose	1.105	3.725	↑	0.004
3.744	pos	Proglumide	1.097	12.37	↑	0.046
6.265	pos	(S)-3'-Hydroxy-4,4'-dimethoxydalbergione	1.096	2.637	↑	0.016
6.609	pos	5,11-dodecadiynoic acid	1.096	1.420	↑	0.004
5.966	neg	Epipodophyllotoxin derivitive	1.095	3.730	↑	0.005
9.309	neg	IL-1R Antagonist	1.094	1.345	↑	0.030
6.629	neg	Milbemycin D	1.092	2.994	↑	0.013
8.905	pos	Polygonal	1.090	1.767	↑	0.024
7.448	neg	N-Lauroyl Cysteine	1.089	5.438	↑	0.001
6.147	neg	2-Geranyl-2',3,4,4'-tetrahydroxydihydrochalcone	1.089	1.597	↑	0.013
5.177	neg	Risbitin	1.086	2.401	↑	0.003
4.200	pos	Methotrimeprazine	1.086	4.656	↑	0.006
5.211	neg	Pubescenol	1.084	1.838	↑	0.030
11.366	neg	(25R)-12alpha-hydroxy-24R,26R-dimethyl-26,27-cyclo-	1.080	2.211	↑	0.031
6.180	pos	Pactimibe	1.076	3.308	↑	0.026
5.789	neg	Non-5-enoylcarnitine	1.076	2.422	↑	0.006
9.557	neg	(9R,10S)-dihydroxystearate	1.075	2.413	↑	0.025
4.416	neg	trans-O-Methylgrandmarin	1.074	2.421	↑	<0.001
6.042	pos	Phytocassane D	1.074	1.599	↑	0.004
5.718	pos	13,14-dihydro-15-keto-tetranor Prostaglandin D2	1.071	1.898	↑	0.001
9.981	pos	12beta, 20S,-trihydroxydammar-23-en-3,25-dione	1.069	2.682	↑	0.024
5.195	neg	CP-471474	1.068	1.892	↑	0.023
10.275	pos	3-dehydroecdysone	1.067	2.809	↑	0.010
5.187	pos	Riesling acetal	1.067	1.515	↑	0.023
7.569	neg	15-Keto-prostaglandin E2	1.065	1.187	↑	0.049
6.858	neg	LysoPI(20:0/0:0)	1.065	5.902	↑	<0.001
4.557	neg	2-Nonene-1,4-diol	1.065	2.503	↑	0.022
7.948	neg	Phytophthora mating hormone alpha1	1.064	1.380	↑	0.011
4.818	neg	18-Oxocortisol	1.062	13.62	↑	0.025
5.596	neg	Methyl clofenapate	1.059	2.692	↑	0.015
4.527	pos	N-3-oxo-hexadec-11(Z)-enoyl-L-Homoserine lactone	1.059	4.384	↑	0.014
8.344	neg	Strobilactone A; (5R,5aS,9aS)-5,9b-Dihydroxy-6,6,9a-	1.058	2.113	↑	0.020
5.966	neg	Moschamindole	1.058	4.396	↑	0.002
2.259	pos	Tyrosyl-Serine	1.057	2.768	↑	0.033
5.596	neg	7-Drimene-11,12,14-triol	1.052	1.562	↑	0.001
4.818	neg	17-phenyl-18,19,20-trinor-prostaglandin E2	1.051	2.940	↑	0.033
4.717	pos	N-Methyl-N-nitro-N'-nitrosoguanidine	1.051	2.324	↑	0.013
5.447	pos	3-[2-Carboxyprop-1-enyl(ethoxycarbonyl)amino]-2-	1.050	3.562	↑	0.003
12.540	pos	PE-Cer(d14:2(4E,6E)/16:0)	1.048	6.661	↑	0.005

4.521	neg	2-Methoxy-5-(2,4-dioxo-5-thiazolidinyl)-N-((4-	1.048	13.62	↑	0.040
5.683	pos	3,4-dimethyl-5-carboxyethyl-2-furanpentanoic acid	1.048	1.933	↑	0.011
8.400	pos	Monomenthyl succinate	1.048	2.057	↑	0.002
4.988	neg	Gibberellin A68	1.048	2.381	↑	0.006
6.823	neg	2-hydroxy-nonadecanoic acid	1.045	2.551	↑	0.007
11.120	neg	LysoPA(P-16:0/0:0)	1.042	2.819	↑	0.001
10.278	neg	22-deoxy-20,21-dihydroxyecdysone	1.042	7.433	↑	0.003
6.350	pos	hydroxytyrosyl acetate	1.041	2.424	↑	0.002
4.814	pos	N-Acetylcystathionine	1.041	4.054	↑	0.045
1.267	neg	Sulfacetamide	1.041	3.635	↑	<0.001
6.219	neg	6-exo-Hydroxyfenchone	1.041	2.091	↑	0.013
0.768	pos	N-[[3-[(2S)-2-Hydroxy-3-[[2-[4-	1.040	3.905	↑	0.012
4.382	pos	Isradipine	1.038	1.642	↑	0.008
4.345	neg	Glucosyl 6-hydroxy-2,6-dimethyl-2E,7-octadienoate	1.038	1.853	↑	0.026
4.468	neg	(1R,2R,4R,8R)-p-Menthane-2,8,9-triol	1.038	3.344	↑	0.003
5.471	neg	5Z-Tetradecenoic acid	1.035	2.785	↑	0.004
5.701	pos	Coriolic acid	1.032	2.185	↑	0.045
7.323	neg	gamma-Secretase Inhibitor II	1.030	35.34	↑	0.028
6.997	pos	3-trans-Caffeoyltormentic acid	1.029	7.675	↑	0.030
7.767	neg	(+)-12-hydroxy-9Z-hexadecenoic acid	1.028	2.252	↑	0.001
3.425	pos	TIADENOL	1.027	2.619	↑	0.037
2.367	neg	EPICATECHIN PENTAACETATE	1.026	4.916	↑	0.003
7.489	pos	4E,6E-undecenal	1.025	1.989	↑	0.019
4.493	pos	Pteroside B	1.023	2.106	↑	0.001
4.460	pos	4,5-Dihydrovomifoliol	1.022	1.485	↑	0.030
9.744	pos	LysoPE(14:0/0:0)	1.022	2.648	↑	0.003
2.113	pos	(2S)-dihydrotricetin	1.022	2.531	↑	0.003
8.093	neg	3,4,7-Trimethyl-2E,6-nonadien-1-ol	1.021	1.836	↑	<0.001
8.211	pos	Ganoderenic acid C	1.017	3.195	↑	0.004
8.687	neg	Arnicolide C; (3S,3aR,4S,4aR,7aR,8R,9aR)-3,4a,8-trimethyl-	1.016	1.573	↑	<0.001
4.588	pos	LysoPC(20:2(11Z,14Z)/0:0)	1.016	29.18	↑	0.011
6.367	pos	Probenecid	1.015	1.661	↑	0.006
6.646	neg	Oxypinnatanine	1.012	1.773	↑	0.033
13.212	pos	DG(20:3(8Z,11Z,14Z)-2OH(5,6)/i-13:0/0:0)	1.010	3.064	↑	<0.001
4.750	pos	Citrunobin	1.007	2.241	↑	0.037

Table S11 Co-enrichment pathways

ID	Metabolic pathways	*P*-value	Substances	Metabolite
hsa04979	Cholesterol metabolism	<0.001	C01921, C05122	Glycocholic acid, Taurocholic acid
hsa04270	Vascular smooth muscle contraction	<0.001	C00219, C14769	Arachidonic acid, 8,9-EET
hsa04913	Ovarian steroidogenesis	0.002	C00219, C14769	Arachidonic acid, 8,9-EET
hsa04216	Ferroptosis	0.003	C00219, C16527	Arachidonic acid, Adrenoic acid

hsa00120	Primary bile acid biosynthesis	0.007	C01921, C05122	Glycocholic Acid, Taurocholic Acid
hsa04912	GnRH signaling pathway	0.017	C00219	Arachidonic acid
hsa00590	Arachidonic acid metabolism	0.018	C00219, C14769	Arachidonic acid
hsa04666	Fc gamma R-mediated phagocytosis	0.023	C00219	Arachidonic acid
hsa04217	Necroptosis	0.028	C00219	Arachidonic acid
