# Supplementary material for: Integrated metagenomics and metabolomics analyses revealed biomarkers in β-casein A2A2-type cows
Source: Front Vet Sci. 2024 Oct 1;11:1438717. doi: 10.3389/fvets.2024.1438717 (PMC11475472; doi:10.3389/fvets.2024.1438717)
Supplement: Supplementary file 2 [file Data_Sheet_2.pdf]

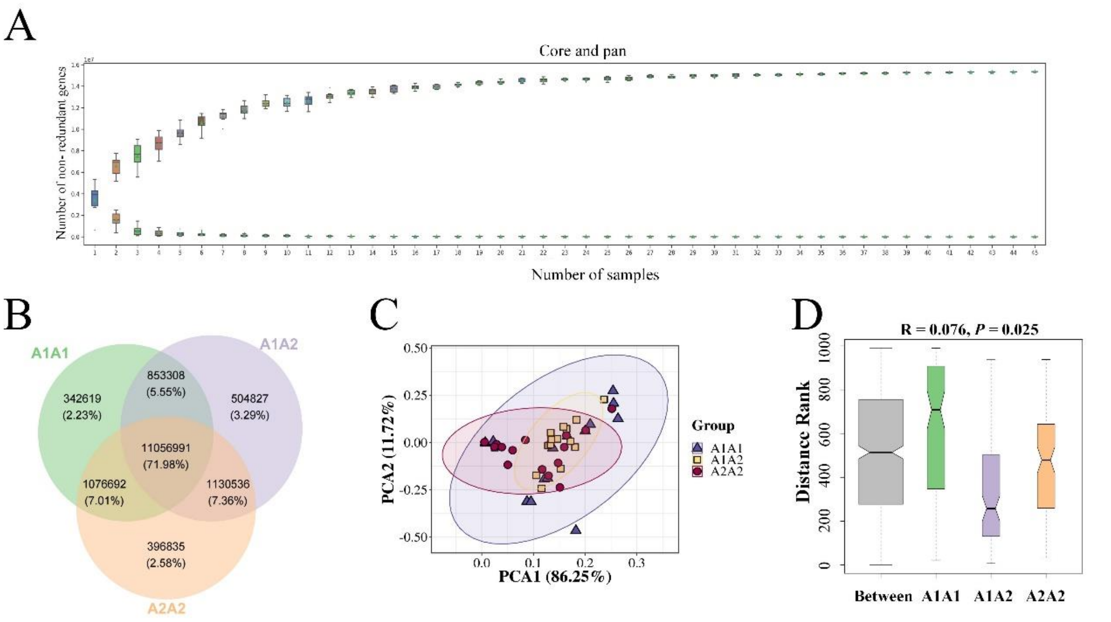

Figure S1 Structural analysis of rumen microbial communities **(A)** Core-pan gene dilution curves; **(B)** Venn diagram of genes among the three groups;**(C)** PCA analysis; **(D)** Anosim analysis graphs.

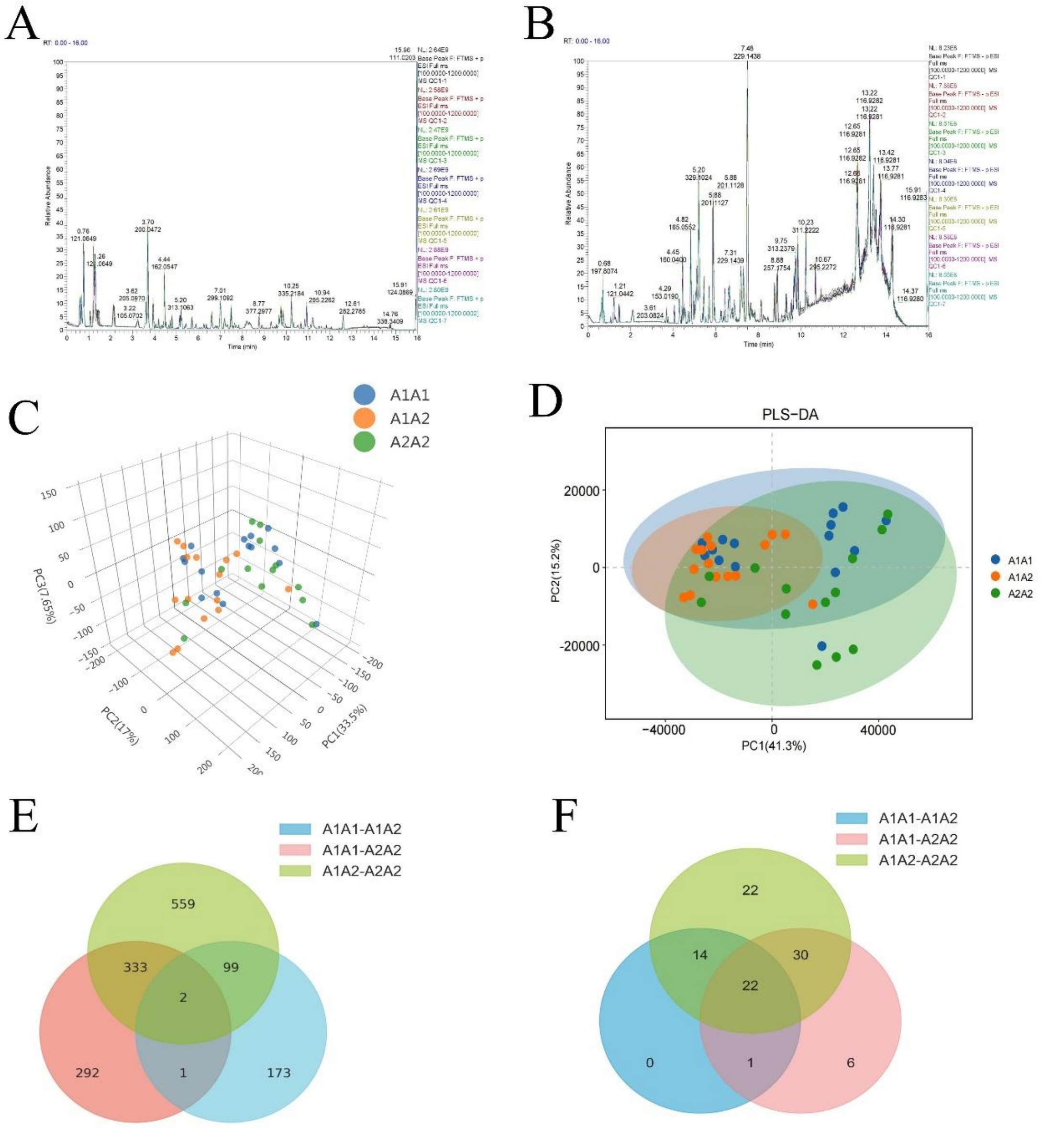

Figure S2 The TIC plot in **(A)** Positive and **(B)** Negative ion mode**(C)** PCA of the three metabolomes; **(D)** PLS-DA of the three metabolomes; **(E)** Wayne plots of the three differential metabolites; **(F)** Wayne plots of the three differential metabolic pathways.

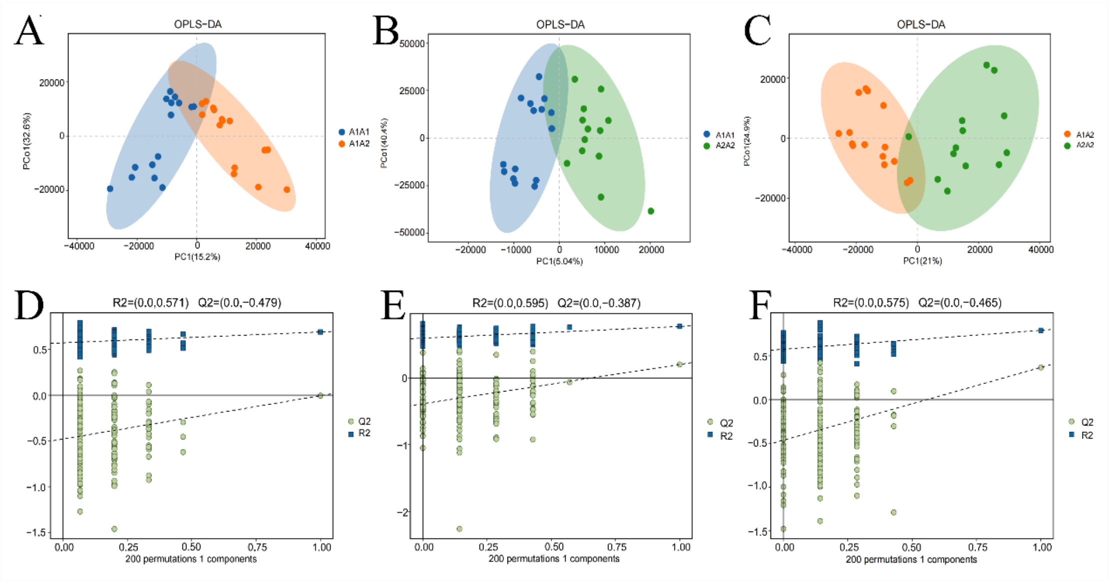

Figure S3 **(A-C)** OPLS-DA of rumen metabolites for groups A1A1, A1A2, and A2A2; **(D-F)** OPLS-DA replacement test plots **(**Further replacement tests of the OPLS-DA model for positive and negative ion pattern data showed that the model was valid for evaluation).

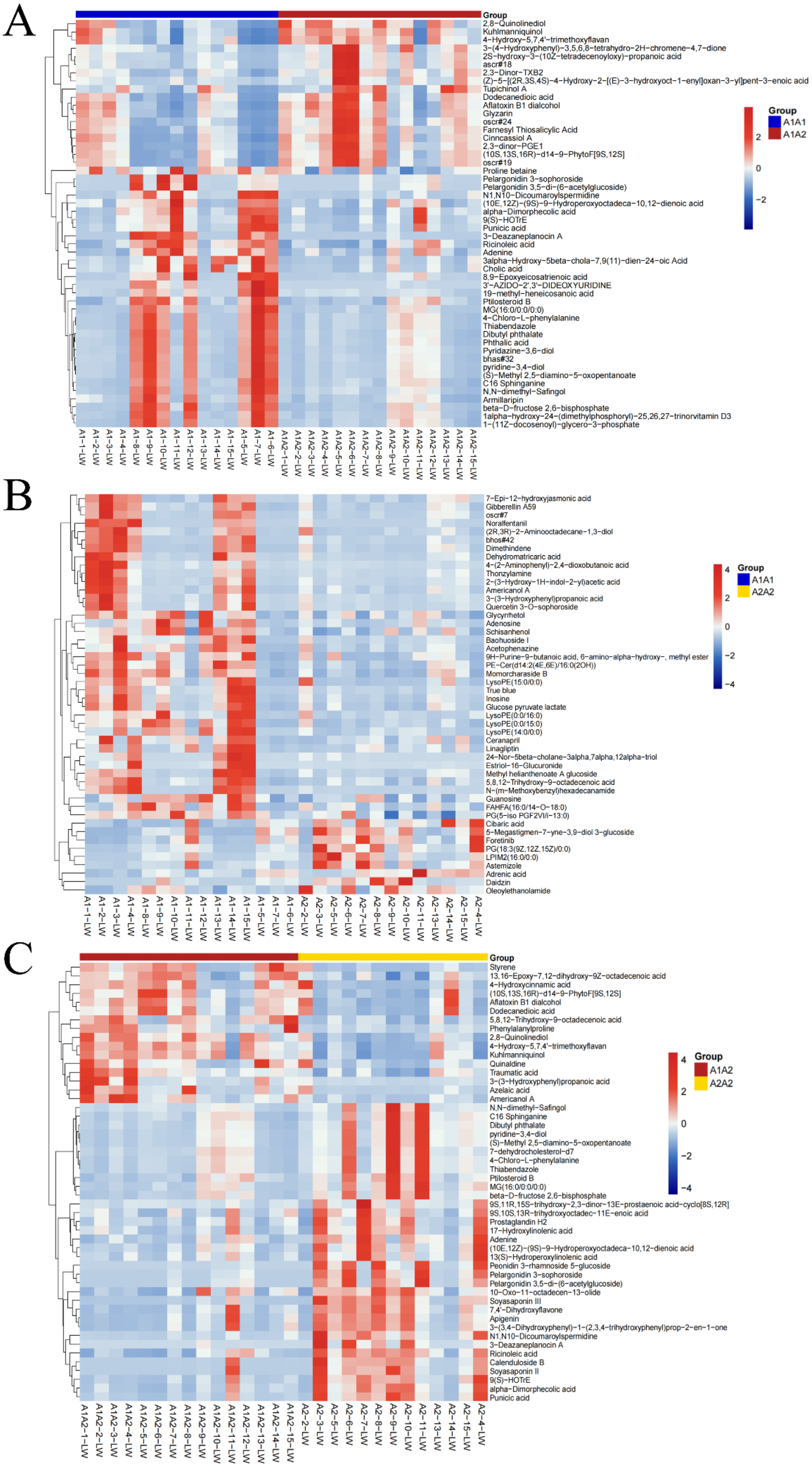
A
Group
2,8−Quinolinediol
Kuhlmanniquinol
4−Hydroxy−5,7,4'−trimethoxyflavan
3−(4−Hydroxyphenyl)−3,5,6,8−tetrahydro−2H−chromene−4,7−dione
2S−hydroxy−3−(10Z−tetradecenoyloxy)−propanoic acid
ascr#18
2,3−Dinor−TXB2
(Z)−5−[(2R,3S,4S)−4−Hydroxy−2−[(E)−3−hydroxyoct−1−enyl]oxan−3−yl]pent−3−enoic acid
Tupichinol A
Dodecanedioic acid
Aflatoxin B1 dialcohol
Glyzarin
oscr#24
Farnesyl Thiosalicylic Acid
Cinncassiol A
2,3−dinor−PGE1
(10S,13S,16R)−d14−9−PhytoF[9S,12S]
oscr#19
Proline betaine
Pelargonidin 3−sophoroside
Pelargonidin 3,5−di−(6−acetylglucoside)
N1,N10−Dicoumaroylspermidine
(10E,12Z)−(9S)−9−Hydroperoxyoctadeca−10,12−dienoic acid
alpha−Dimorphecolic acid
9(S)−HOTrE
Punicic acid
3−Deazaneplanocin A
Ricinoleic acid
Adenine
3alpha−Hydroxy−5beta−chola−7,9(11)−dien−24−oic Acid
Cholic acid
8,9−Epoxyeicosatrienoic acid
3'−AZIDO−2',3'−DIDEOXYURIDINE
19−methyl−heneicosanoic acid
Ptilosteroid B
MG(16:0/0:0/0:0)
4−Chloro−L−phenylalanine
Thiabendazole
Dibutyl phthalate
Phthalic acid
Pyridazine−3,6−diol
bhas#32
pyridine−3,4−diol
(S)−Methyl 2,5−diamino−5−oxopentanoate
C16 Sphinganine
N,N−dimethyl−Safingol
Armillaripin
beta−D−fructose 2,6−bisphosphate
1alpha−hydroxy−24−(dimethylphosphoryl)−25,26,27−trinorvitamin D3
1−(11Z−docosenoyl)−glycero−3−phosphate
A1−1−LW A1−2−LW A1−3−LW A1−4−LW A1−8−LW A1−9−LW A1−10−LW A1−11−LW A1−12−LW A1−13−LW A1−14−LW A1−15−LW A1−5−LW A1−7−LW A1−6−LW A1A2−1−LW A1A2−2−LW A1A2−3−LW A1A2−4−LW A1A2−5−LW A1A2−6−LW A1A2−7−LW A1A2−8−LW A1A2−9−LW A1A2−10−LW A1A2−11−LW A1A2−12−LW A1A2−13−LW A1A2−14−LW A1A2−15−LW
Group
A1A1
A1A2
2
0
−2
B
7−Epi−12−hydroxyjasmonic acid
Gibberellin A59
oscr#7
Noralfentanil
(2R,3R)−2−Aminooctadecane−1,3−diol
bhos#42
Dimethindene
Dehydromatricaric acid
4−(2−Aminophenyl)−2,4−dioxobutanoic acid
Thonzylamine
2−(3−Hydroxy−1H−indol−2−yl)acetic acid
Americanol A
3−(3−Hydroxyphenyl)propanoic acid
Quercetin 3−O−sophoroside
Glycyrrhetol
Adenosine
Schisanhenol
Baohuoside I
Acetophenazine
9H−Purine−9−butanoic acid, 6−amino−alpha−hydroxy−, methyl ester
PE−Cer(d14:2(4E,6E)/16:0(2OH))
Momorcharaside B
LysoPE(15:0/0:0)
True blue
Inosine
Glucose pyruvate lactate
LysoPE(0:0/16:0)
LysoPE(0:0/15:0)
LysoPE(14:0/0:0)
Ceranapril
Linagliptin
24−Nor−5beta−cholane−3alpha,7alpha,12alpha−triol
Estriol−16−Glucuronide
Methyl helianthenoate A glucoside
5,8,12−Trihydroxy−9−octadecenoic acid
N−(m−Methoxybenzyl)hexadecanamide
Guanosine
FAHFA(16:0/14−O−18:0)
PG(5−iso PGF2VI/i−13:0)
Cibaric acid
5−Megastigmen−7−yne−3,9−diol 3−glucoside
Foretinib
PG(18:3(9Z,12Z,15Z)/0:0)
LPIM2(16:0/0:0)
Astemizole
Adrenic acid
Daidzin
Oleoylethanolamide
A1−1−LW A1−2−LW A1−3−LW A1−4−LW A1−8−LW A1−9−LW A1−10−LW A1−11−LW A1−12−LW A1−13−LW A1−14−LW A1−15−LW A1−5−LW A1−7−LW A1−6−LW A2−2−LW A2−3−LW A2−5−LW A2−6−LW A2−7−LW A2−8−LW A2−9−LW A2−10−LW A2−11−LW A2−13−LW A2−14−LW A2−15−LW A2−4−LW
Group
A1A1
A2A2
4
2
0
−2
−4
C
Group
Styrene
13,16−Epoxy−7,12−dihydroxy−9Z−octadecenoic acid
4−Hydroxycinnamic acid
(10S,13S,16R)−d14−9−PhytoF[9S,12S]
Aflatoxin B1 dialcohol
Dodecanedioic acid
5,8,12−Trihydroxy−9−octadecenoic acid
Phenylalanylproline
2,8−Quinolinediol
4−Hydroxy−5,7,4'−trimethoxyflavan
Kuhlmanniquinol
Quinaldine
Traumatic acid
3−(3−Hydroxyphenyl)propanoic acid
Azelaic acid
Americanol A
N,N−dimethyl−Safingol
C16 Sphinganine
Dibutyl phthalate
pyridine−3,4−diol
(S)−Methyl 2,5−diamino−5−oxopentanoate
7−dehydrocholesterol−d7
4−Chloro−L−phenylalanine
Thiabendazole
Ptilosteroid B
MG(16:0/0:0/0:0)
beta−D−fructose 2,6−bisphosphate
9S,11R,15S−trihydroxy−2,3−dinor−13E−prostaenoic acid−cyclo[8S,12R]
9S,10S,13R−trihydroxyoctadec−11E−enoic acid
Prostaglandin H2
17−Hydroxylinolenic acid
Adenine
(10E,12Z)−(9S)−9−Hydroperoxyoctadeca−10,12−dienoic acid
13(S)−Hydroperoxylinolenic acid
Peonidin 3−rhamnoside 5−glucoside
Pelargonidin 3−sophoroside
Pelargonidin 3,5−di−(6−acetylglucoside)
10−Oxo−11−octadecen−13−olide
Soyasaponin III
7,4'−Dihydroxyflavone
Apigenin
3−(3,4−Dihydroxyphenyl)−1−(2,3,4−trihydroxyphenyl)prop−2−en−1−one
N1,N10−Dicoumaroylspermidine
3−Deazaneplanocin A
Ricinoleic acid
Calenduloside B
Soyasaponin II
9(S)−HOTrE
alpha−Dimorphecolic acid
Punicic acid
A1A2−1−LW A1A2−2−LW A1A2−3−LW A1A2−4−LW A1A2−5−LW A1A2−6−LW A1A2−7−LW A1A2−8−LW A1A2−9−LW A1A2−10−LW A1A2−11−LW A1A2−12−LW A1A2−13−LW A1A2−14−LW A1A2−15−LW A2−2−LW A2−3−LW A2−5−LW A2−6−LW A2−7−LW A2−8−LW A2−9−LW A2−10−LW A2−11−LW A2−13−LW A2−14−LW A2−15−LW A2−4−LW
Group
A1A2
A2A2
4
2
0
−2
−4

Figure S4 **(G-I)** Differential hierarchical cluster analysis of the top 50 metabolites of rumen metabolites in groups A1A1, A1A2 and A2A2 (heat map).

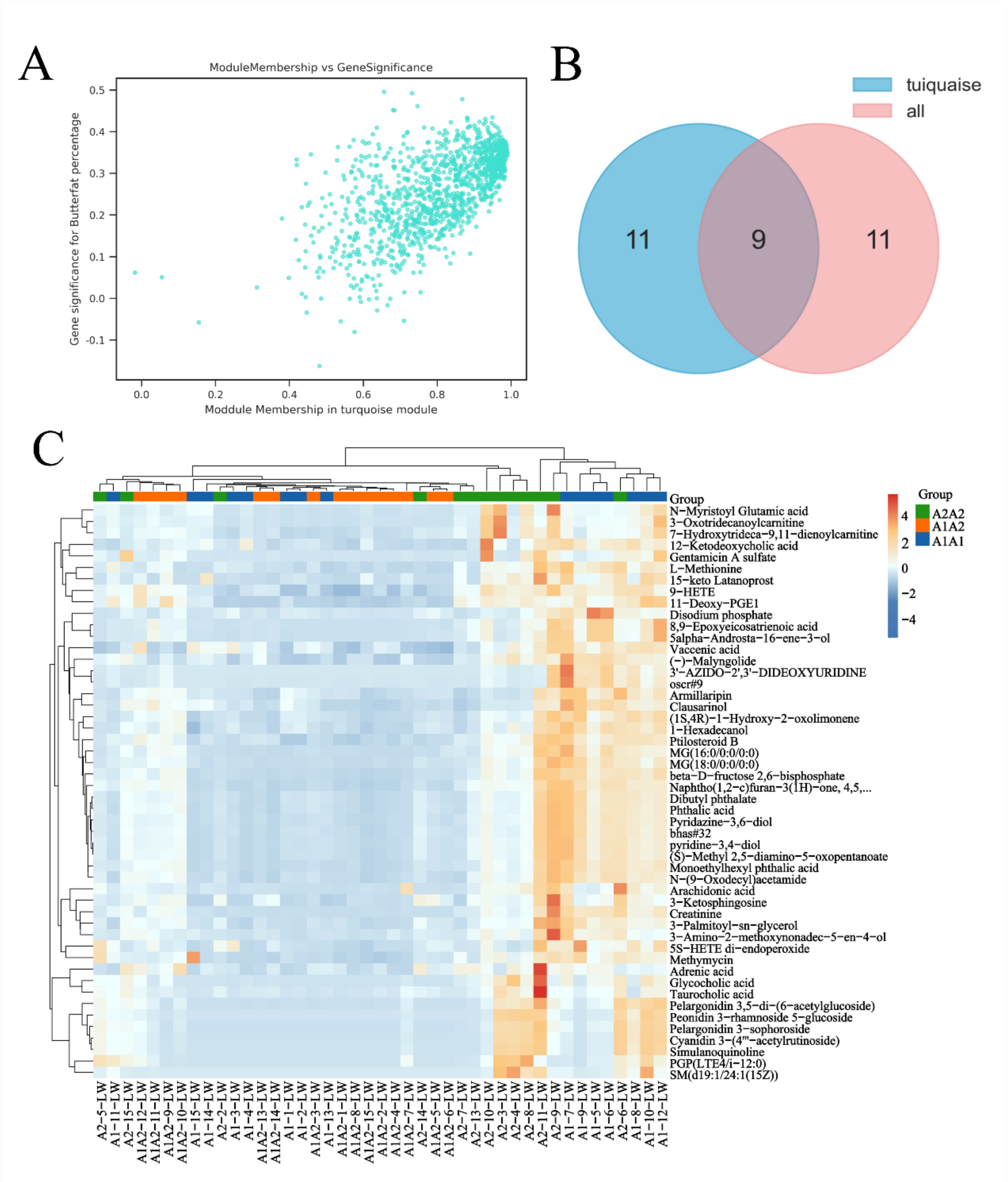

Figure S5 **(A)**Scatter plot of metabolites versus turquoise module members (MM);**(B)** MEturquoise metabolite set enrichment analysis (heatmap); **(C)** Wayne plots of MEturquoise top 20 differential metabolic pathways and top 20 differential metabolic pathways among the three groups.

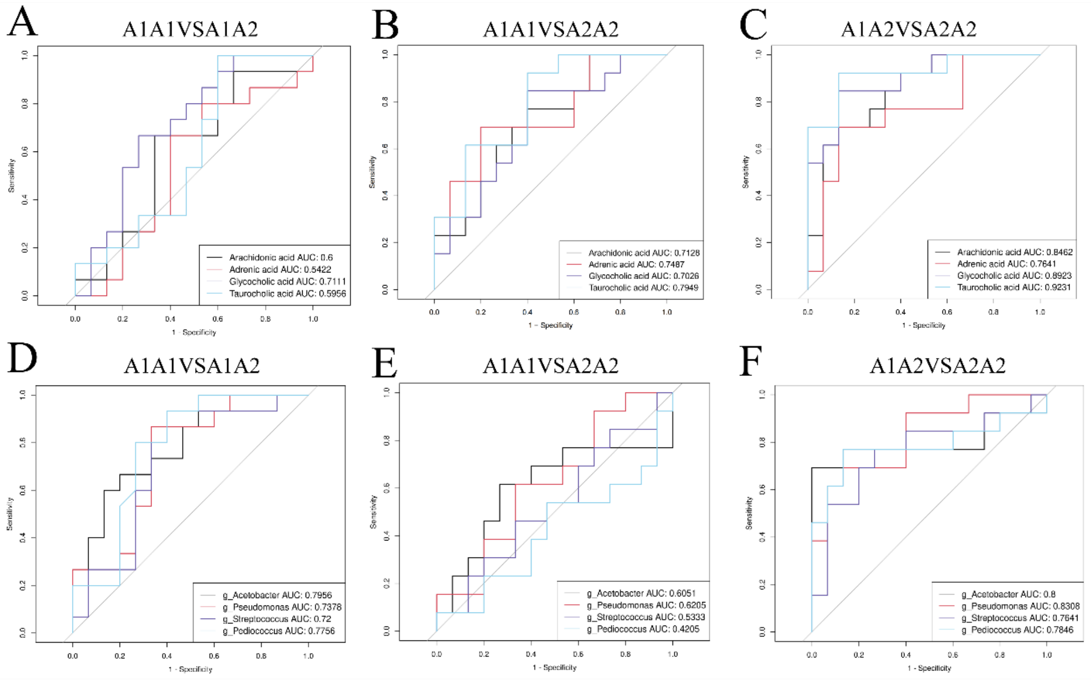

Figure S6 **(A-C)** ROC curves of AA, AdA, GCA, and TCA; (**D-F)** ROC curves of *g_Acetobacter, g_Pseudoxanthomonas, g_Streptococcus* and *g_Pediococcus*.
